# Mohs math – where the error hides

**DOI:** 10.1186/1471-5945-6-10

**Published:** 2006-12-06

**Authors:** Jeffrey I Ellis, Tatiana Khrom, Anthony Wong, Mario O Gentile, Daniel M Siegel

**Affiliations:** 1 Department of Dermatology, SUNY Downstate Medical Center, Brooklyn, New York, 11203, USA; 2 Jxnstudio.com, Philadelphia, Pennsylvania, USA

## Abstract

**Background:**

Mohs surgical technique allows a full view of surgical margins and has a reported cure rate approaching 100%.

**Method:**

A survey amongst Mohs surgeons was performed to assess operator technique. In addition, an animated clay model was constructed to identify and quantify tissue movement seen during the processing of Mohs surgical specimens.

**Results:**

There is variability in technique used in Mohs surgery in regards to the thickness of layers, and the number of blocks layers are cut into. A mathematical model is described which assesses the clinical impact of this variability.

**Conclusion:**

Our mathematical model identifies key aspects of technique that may contribute to error. To keep the inherent error rate at a minimum, we advocate minimal division and minimal physical thickness of Mohs specimens.

## Background

Over the past sixty years, Mohs micrographic surgery has become the standard of care in the management and treatment of many skin cancers. Unlike standard vertical sectioning, the horizontal sectioning utilized by Mohs technique allows a full view of surgical margins [1] and has a reported cure rate between 88 to 100% [2-7]. Differences in operator technique are already known [8,9], however their impact into the ability to fully view the surgical margins have not been defined. This paper is divided into two parts; Part I: A survey of the techniques of practicing Mohs Surgeons. Part II: A mathematical model is described which assesses the clinical impact of technique variability.

## Methods

### Survey methods

An e-mail survey was conducted utilizing several dermatology e-mail lists including **RxDERM**-L at ucdavis and the Academic Dermatologic Surgeons listserve. 28 Mohs surgeons responded, and were asked the questions seen in [Table 1].

**Table 1:** Survey Questions

1. How many years have you been performing Mohs Surgery?
2. Who cuts your excised layer into blocks?
a. You
b. Fellow
c. Tech
3. For specimens ranging from 1–4 cm, on average
a. How many blocks is the excised layer cut into when processing?
b. What is the thickness (depth) of your first Mohs layer?

### Mathematical model methods

To best appreciate the following mathematical model, it is crucial for one to be familiar with the processing of tissue in Mohs surgery. For those not involved with Mohs surgery on a daily basis, this can be challenging to visualize. As such, a clay animation of ideal Mohs tissue processing is provided to clarify the geometry of expected tissue movement during processing [see Additional file 1].

**Figure 7:**
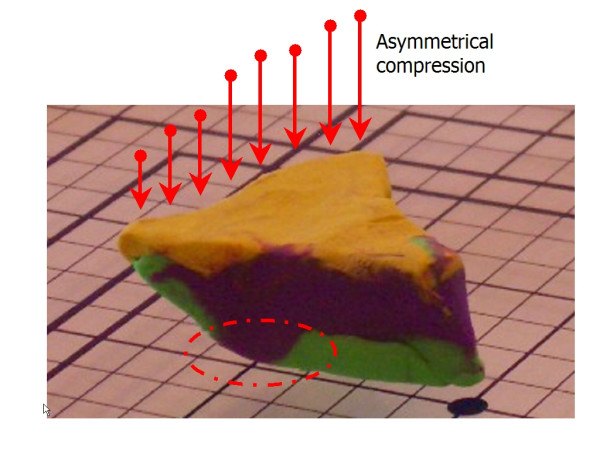
Clay model of asymmetrical compression.

**Figure 8:**
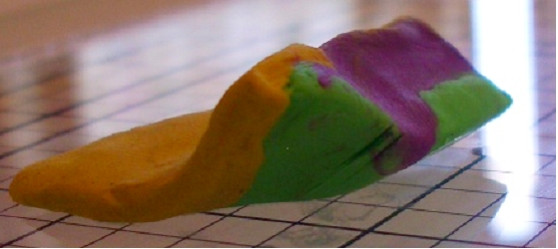
Clay model of asymmetrical compression.

**Figure 9:**
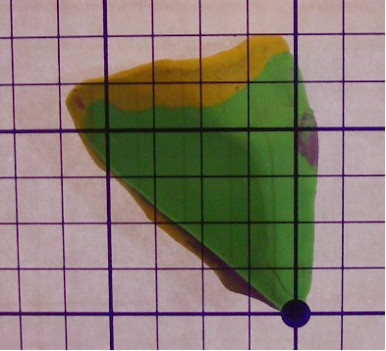
Clay model of asymmetrical compression.

Using this clay model, one can begin to imagine where errors may occur during tissue processing. The first example of processing error can we call "Edge Lift Roll". An animation of this potential processing error can be seen at [see Additional file 2]. In this case, when the tissue is processed, *asymmetrical compression *is applied to the tissue (Figure 7). This results in a rolling of the specimen while processing, and may result in one edge lifting from the plane of sectioning (Figure 8, 9). This can potentially result in a false negative, and a future recurrence of tumor. One can also easily imagine that asymetircial compression could also lead to an edge folding into the plane of sectioning thereby causing a false positive.

**Figure 10:**
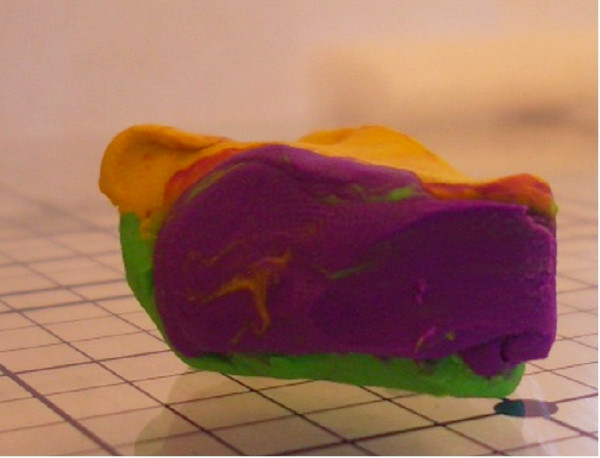
Clay model of a thick layer.

**Figure 11:**
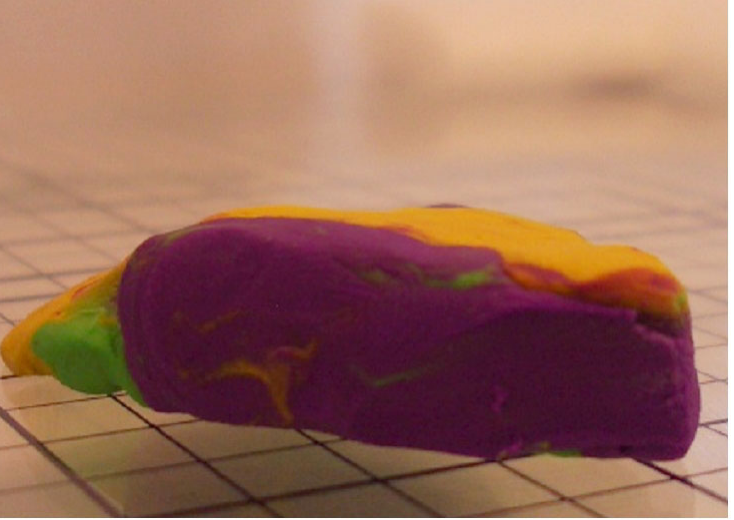
Clay model of a thick layer (squash error).

**Figure 12:**
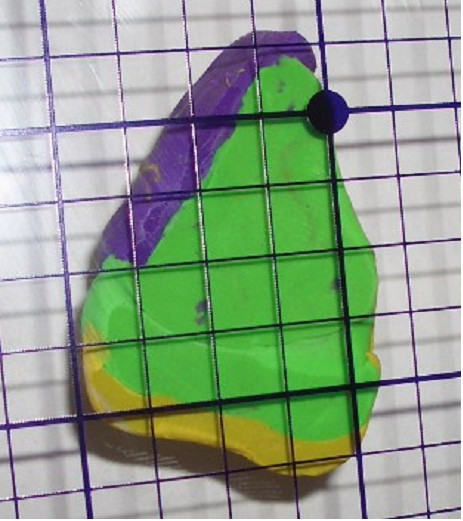
Clay model of a thick layer (squash error).

"The Squash" [see Additional file 3] can occur if a block is thick with dermis or fat bellowing from the midsection (Figure 10). If redundancy from the core of the block slides into the plane of sectioning (Figure 11, 12) – one may observe a false positive, resulting in additional and unnecessary layer harvesting.

**Figure 13:**
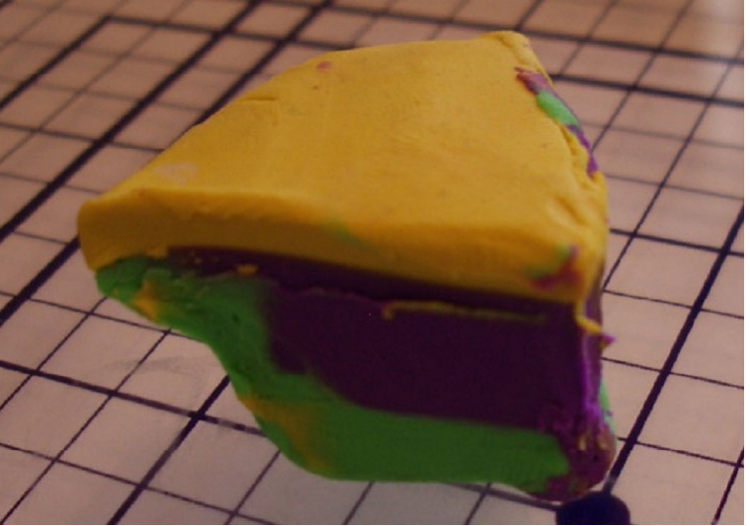
Clay model of a tip lift.

**Figure 14:**
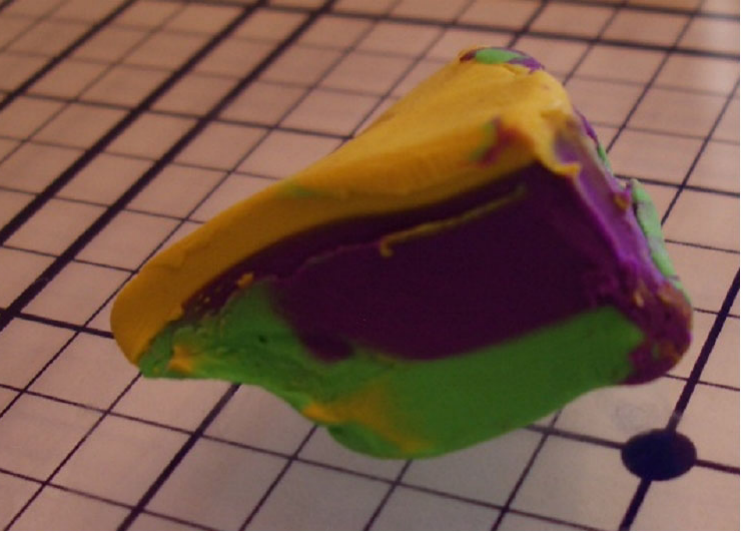
Clay model of a tip lift.

**Figure 15:**
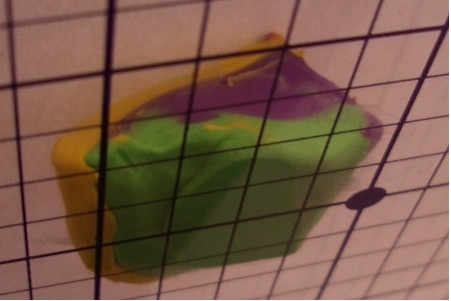
Clay model of a tip lift.

"Tip Lift" [see Additional file 4] may occur if during the attempt to flatten the outer edge of a block (Figure 13), the inner tip lifts from the plane of sectioning (Figure 14, 15). This may result in a false negative, and potentiates future recurrence.

**Figure 16:**
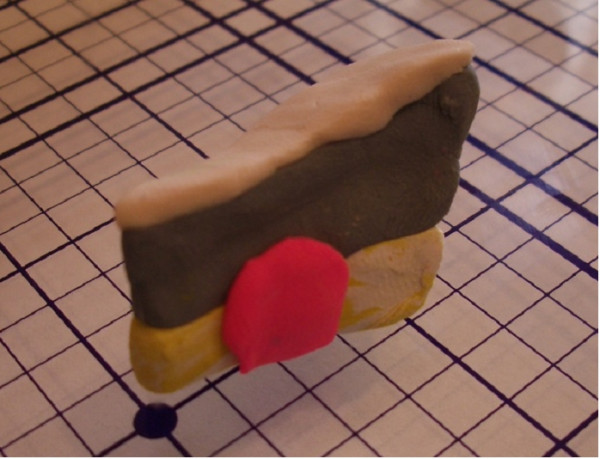
Clay model of an exaggerated thin section.

**Figure 17:**
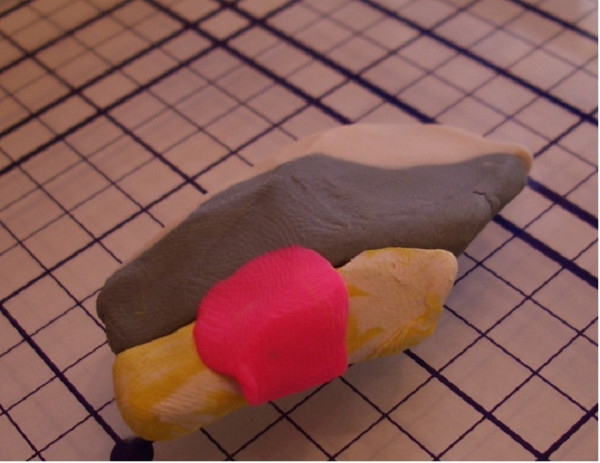
Clay model of an exaggerated thin section.

**Figure 18:**
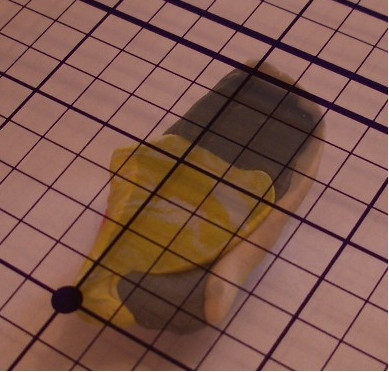
Clay model of an exaggerated thin section.

"Thin Section Collapse" [see Additional file 5] error is based on an exaggerated model where a layer is cut into thin slivers (Figure 16). We mention it here as a subtle variation may occur in clinical practice. In this case, when an attempt is made to flatten the epidermis, the tissue collapses and rolls to one side (Figure 17, 18). In this example, the tumor that was reaching the base of the specimen is lifted away from the base and is removed from the plane of sectioning. This can potentially result in either a false positive or a false negative – as a tumor can be lifted away (as demonstrated here), or brought into the plane of sectioning.

## Results

### Survey results

**Figure 1:**
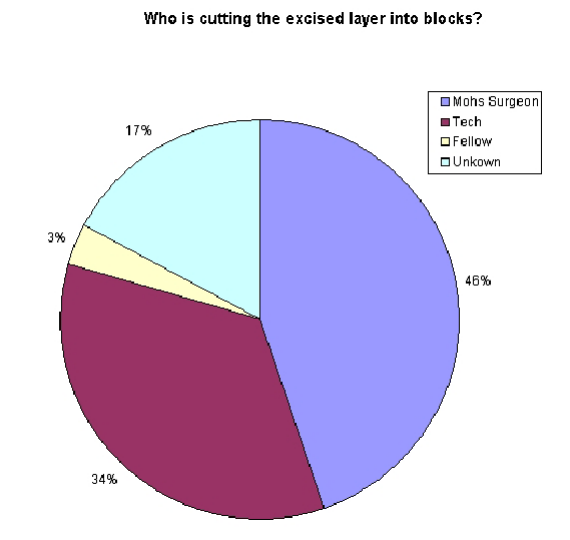
Pie chart: Who is cutting the excised layer into blocks?

**Figure 2:**
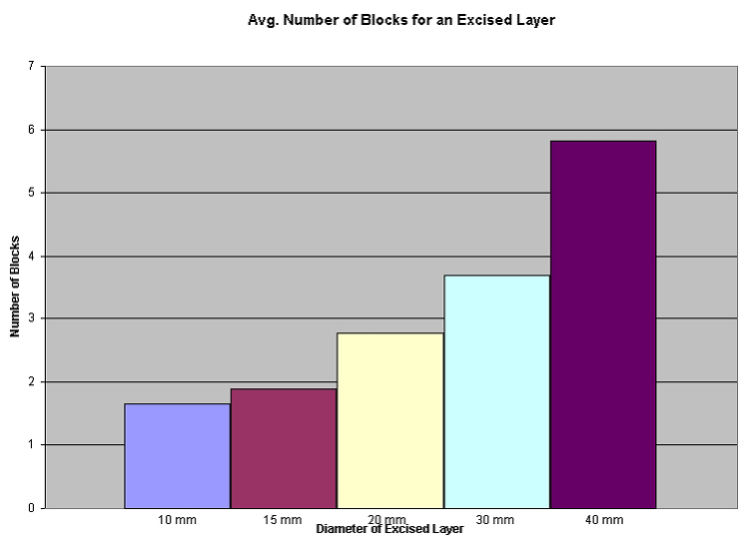
Bar graph: Average number of blocks for an excised layer.

**Figure 3:**
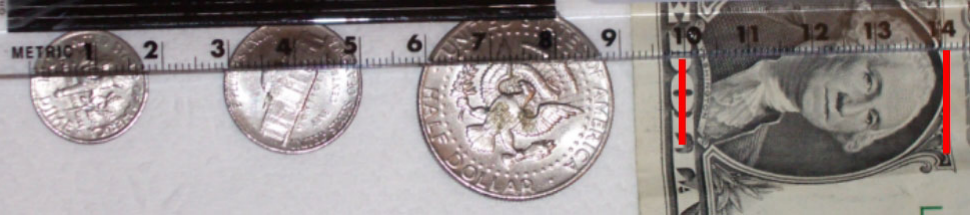
Illustration of the size of US coins.

**Figure 4:**
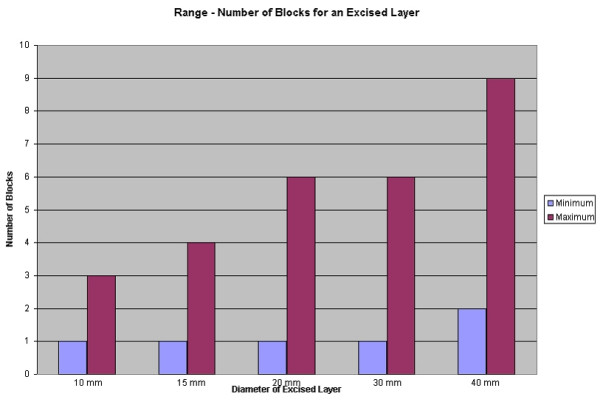
Bar graph: Range – Number of blocks for an excised layer.

Experience ranged from 2 to 29 years, with a mean of 12 years. 46% of the time, the Mohs Surgeon reported cutting the excised layer into blocks (see Figure 1). As expected, the average number of blocks needed for a given layer increased from 1 or 2 blocks for a 10 mm specimen to 6 blocks for a 40 mm specimen (see Figure 2). However, when evaluating the *range *of this data, one sees that there is great variability. To make the sizes referred to less abstract, consider (figure 3). Here we see that a dime is about 15 mm, a nickel is 20 mm, half dollar 30 mm, and portrait of George Washington 40 mm. Some surgeons reported processing a dime size layer whole, while others reported cutting it into three blocks. Similarly, some would process George Washington's portrait (40 mm) into 2 blocks – while others would process the same layer into 6 blocks (see figure 4).

**Figure 5:**
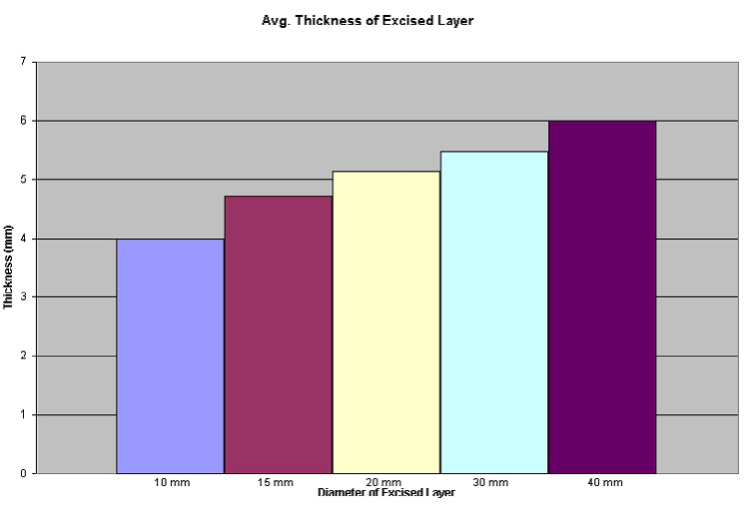
Bar graph: Average thickness of an excised layer.

**Figure 6:**
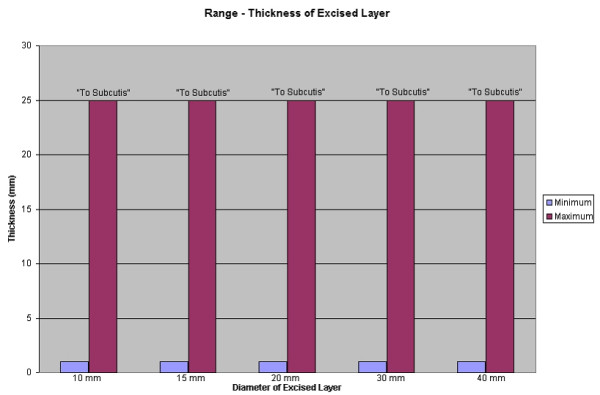
Bar graph: Range – Thickness of excised layers.

Regarding the thickness of Mohs layer's, similar variability was reported. Though the average depth showed progressive thickening, as may have been expected (see figure 5), analysis of the *range *reveals that some surgeons tend take thin layers while others tend to cut to subcutis – regardless of specimen size (see figure 6).

The question remains... does it matter? A mathematical model was created to assess the importance of these Mohs technique variables.

### Mathematical model results, derivation of a mathematical proof

Careful analysis of tissue movement in "ideal" processing, and the errors that may occur when tissue is processed allows one to derive a mathematical expression. This is a useful exercise because analysis of the expression can allow one to draw conclusions related to the specific aspects of the technique that contribute most to potentiating error.

**Figure 19:**
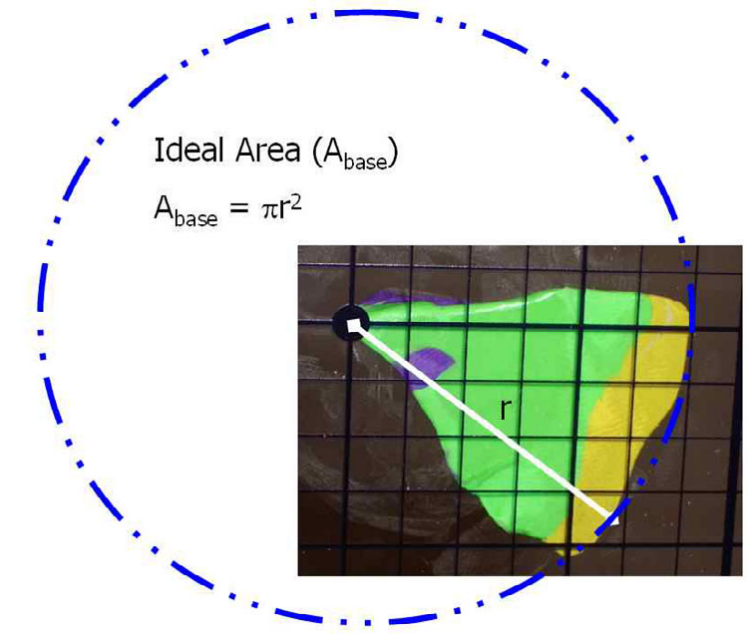
Mathematical proof, demonstrating ideal area.

**Figure 20:**
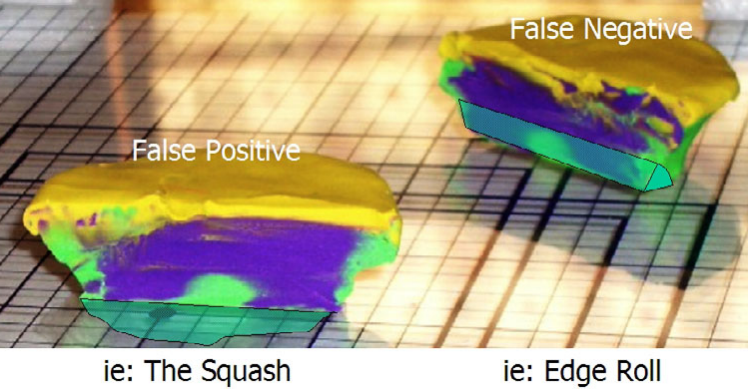
Mathematical proof, false positive and false negative.

It is clear that for any layer, there is an "ideal area" (Figure 19) that represents the perfect footprint of the tissue, allowing for 100% visualization of the surgical margins. Errors in tissue processing will result in either a loss of ideal area (false negative), or gain in area (false positive). Review of the models above identifies that the loss or gain in processed tissue is related to the area of the *sidewalls *of the block. (Figure 20) This is a part of the tissue often overlooked – as it has no significance if the tissue is processed correctly. To explain the errors identified here, it is important to precisely calculate the area of these sidewalls. While tissue has dynamic properties categorized as stress relaxation and creep, we have ignored these in this model, as their impact is minimal on the analysis we present.

**Step 1**: Calculation of ideal area (Figure 19)

A_base _= Πr^2^

**Figure 22:**
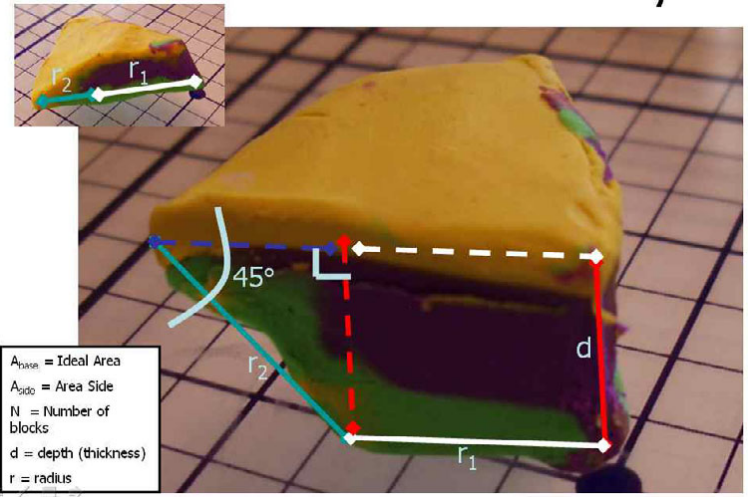
Mathematical proof, area of the side walls.

**Step 2**: Calculation of the area of the side wall of one block (Figure 22)

A_side _= (d)(r_1_) + 1/2(d)^2^

**Figure 21:**
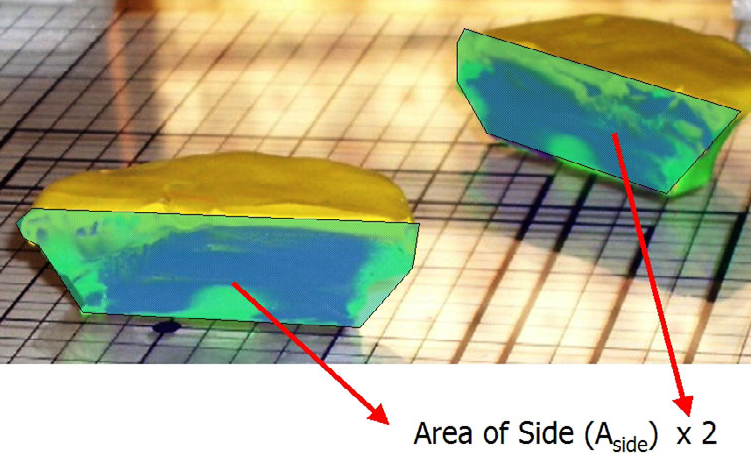
Mathematical proof, area of the side walls.

**Step 3**: Calculation of the total area of the side walls (Figure 21)

Total area of the side walls = (N) × (A_side_)

**Step 4**: A false negative is the ideal area (A_base_) minus a percentage of A_side_. And a false positive is the idea area plus a percentage of A_side_.

Let k = the percentage roll, falling between 0 and 1.

Substituting what we know, and performing some simple trigonometry, we continue our derivation as shown in [Table 2]

**Table 2:** Mathematical proof

**False (-)**	Ideal	False (+)
A_base _- k(A_side_)(N)	Abase	A_base _+ k(A_side_)(N)
Πr^2 ^- k(A_side_)(N)	Πr^2^	Πr^2 ^+ k(A_side_)(N)
Πr^2 ^- k((d)(r_1_) + 1/2(d)^2^)(N)	Πr^2^	Πr^2 ^+ k((d)(r_1_) + 1/2(d)^2^)(N)
Πr^2 ^- k((d)((r - d/0.851)) + 1/2(d)^2^)(N)	Πr^2^	Πr^2 ^+ k((d)((r - d/0.851)) + 1/2(d)^2^)(N)

We must solve for r_1 _(See Figure 22) *Note: although r1 and r2 are not collinear, they become collinear when the Mohs tissue is processed (see *Additional file 1* for review of movement during processing) *

**Figure 23:**
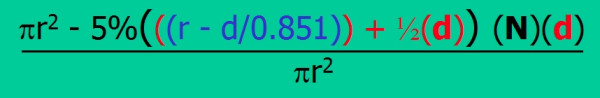
Mathematical proof, mathematical formula of predicted error.

r = r_1 _+ r_2_

r_1 _= r - r_2_

Sin (45) = d/r_2_

r_2 _= d/Sin (45) = d/0.851

r_1 _= (r - d/0.851)

**Step 5**: We can place the expression of error *over *the ideal area, to create a mathematical formula that predicts error. This formula will produce the value that is equal to the percentage of tissue that is lost from the ideal preparation of a specimen. If we assume only a 5% roll (k = 0.05), we have the following expression (see Figure 23) (Note: for simplicity, let us assume that k is the same on each side)

**Figure 33:**
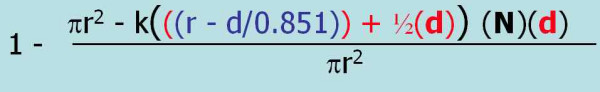
Mathematical formula of predicted viewable margin.

Alternatively, we could calculate the percentage of the tissue that *is viewed *on a prepared histological preparation of a Mohs slide. The percentage of viewable surface area would be calculated by subtracting the result of Figure 23 from 1, as shown in Figure 33.

**Figure 25:**
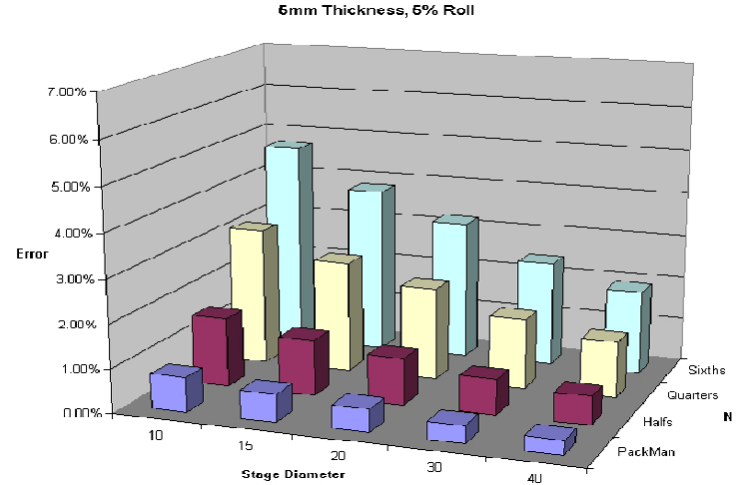
Predicted error for a 5 mm thick layer with 5% roll.

The final mathematical expression derived from the proof above can be seen in Figure 25. Of note, N (the number of blocks), and d (depth of layer) are directly related to the degree of processing error anticipated. This final expression is demonstrated with real numbers to illustrate its importance.

**Figure 24:**
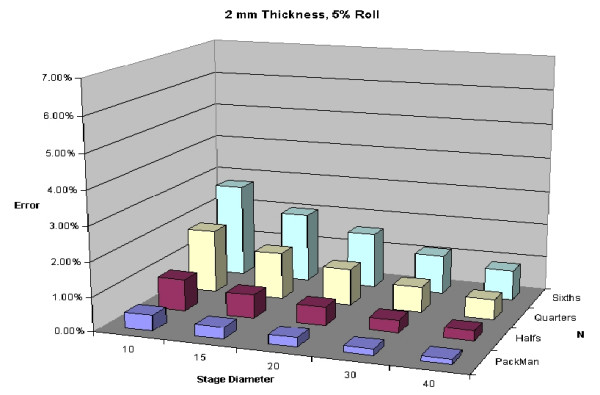
Predicted error for a 2 mm thick layer with 5% roll.

**Figure 26:**
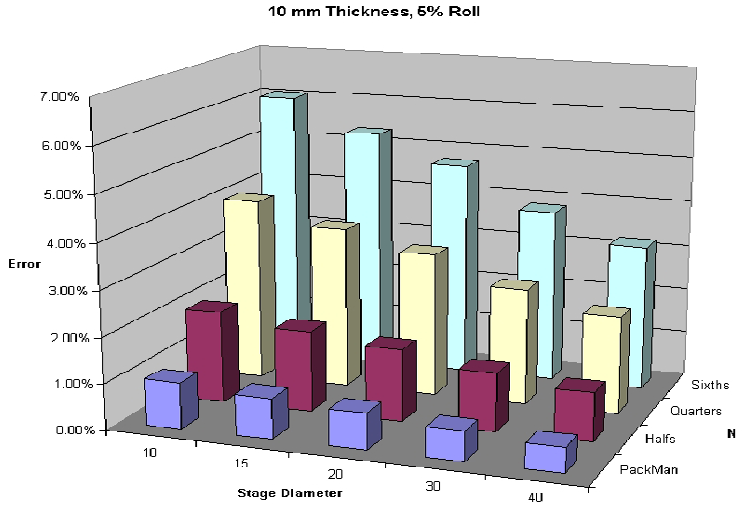
Predicted error for a 10 mm thick layer with 5% roll.

**Figure 27:**
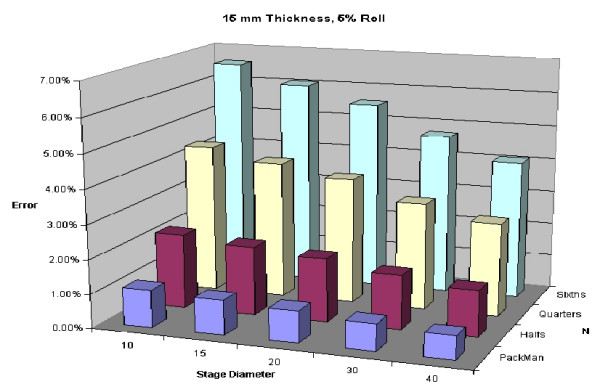
Predicted error for a 15 mm thick layer with 5% roll.

To illustrate variability of thickness of specimens, Figures 24, 25, 26, 27 are shown. All of these figures assume a 5% roll (k = 0.05). Several conclusions can be made by looking at this series of figures. First, it is clear that the greater the number of blocks (N), the higher the predicted error. Looking across the figures, one sees how anticipated error grows with thicker layers.

**Figure 28:**
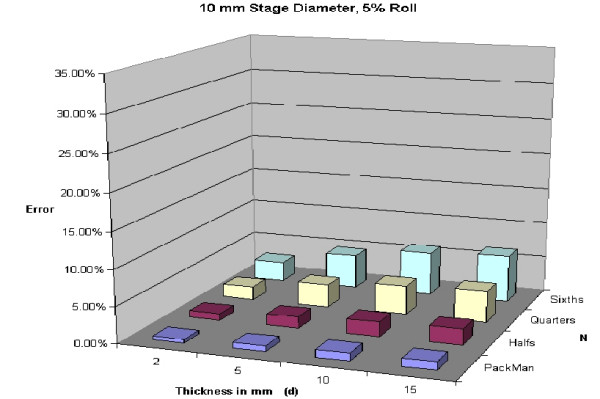
Predicted error for a 10 mm diameter layer, with 5% roll.

**Figure 29:**
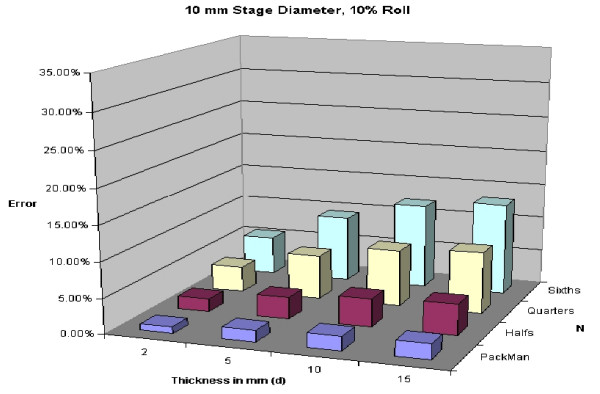
Predicted error for a 10 mm diameter layer, with a 10% roll.

**Figure 30:**
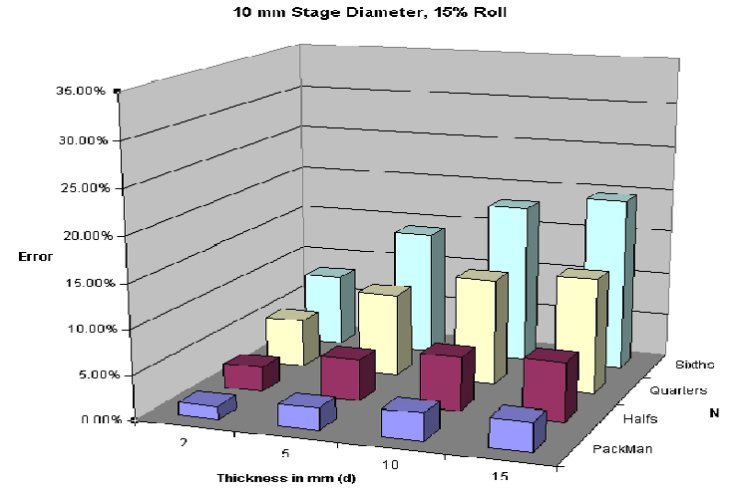
Predicted error for a 10 mm diameter layer, with a 15% roll.

**Figure 31:**
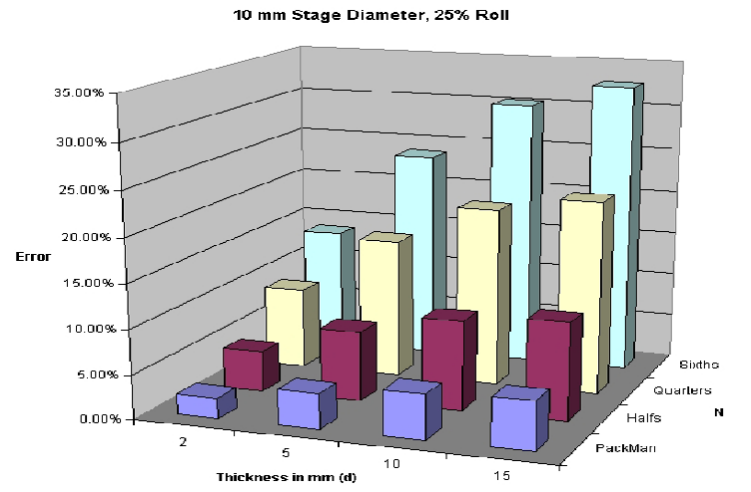
Predicted error for a 10 mm diameter layer, with a 25% roll.

A 5% roll is an estimation used, but is not based on any known data. Figures 28, 29, 30, 31 demonstrate changes in error that can be anticipated if there is greater than a 5% roll, with Figure 28 illustrating a 5% roll, and Figure 31 a 25% roll.

**Figure 32:**
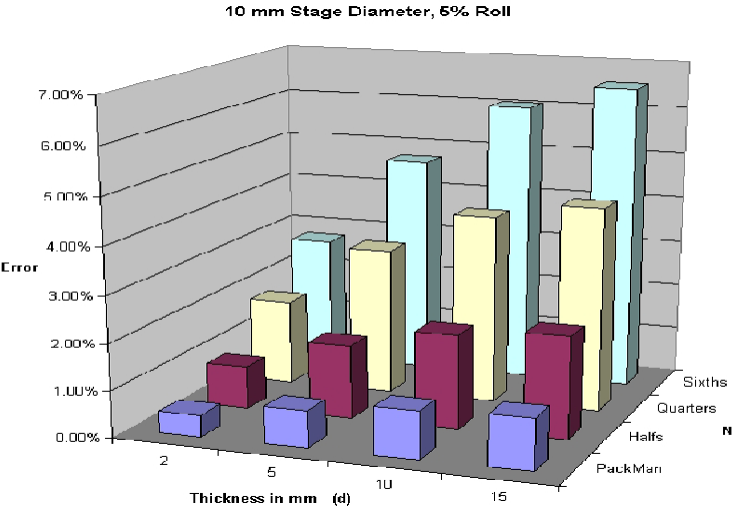
Predicted error for a 10 mm diameter layer, with a 5% roll.

Figure 32 demonstrates a proposed clinically relevant set of parameters. Illustrated is anticipated error for a 10 mm layer, with a 5% roll during processing. One sees as the thickness increases from 2 to 15 mm, anticipated error grows. It is also apparent that as N grows, so does the anticipated error. Error rates reported in this graph are between 1 and 7%, consistent with reported rates of recurrence and far below the recurrence rates seen with standard excision and breadloaf sectioning.

## Discussion

Is recurrence of a tumor after Mohs surgery always a result of error? Persistent tumor may be related to "difficulties of anatomic site[10], tumor size and histological subtype[11], as well as observer error in histological interpretation and potential tumor multifocality[12]" [13]. There are also many processing errors that may occur including inaccurate mapping, tissue staining, and tissue preparation for sectioning. It is clear that in order to maximize the value of the technique, processing of tissue must be as ideal as possible.

The importance of processing tissue in an 'ideal way' is not a new one. The benefits of processing a layer as one block have been previously described [14]. In addition, several authors have suggested techniques to facilitate obtaining quality and complete horizontal sections [15-17].

It seems prudent to anticipate some questions that this paper may raise, and provide answers at this time. One frequently asked question is "Wouldn't you notice missing tissue (i.e.: edge role)" The answer is simply no. Remember that the clay models show an exaggerated event to help illustrate a potential event. If only 5% of the tissue rolled, this would unlikely be perceivable. Even if it were perceived that this tissue seemed "smaller", it would be easy to disregard this fact as anticipated tissue shrinkage [18].

Another question often asked relates to tissue dyes. In the models presented, the clay was not marked with an orientation dye. If the edge lifted, wouldn't the marked edge be lost? The answer is that it depends. As we know, the orientation dye we use is far from precise, and often "bleeds" slightly. It is easy to imagine tissue could be removed form the plane of section, while some orientation dye remains. One must remember that the only absolute edge is an epidermal edge; it is the non-epithelial edges that are subject to the errors we have demonstrated. As tissue dyes do "bleed", they cannot be considered absolute boundary markers.

Finally, curetting or debulking a tumor may have additional benefit related to processing. Though this is controversial amongst Mohs surgeons, removing the bulk of a tumor will serve to significantly decrease the thickness of a Mohs layer. In doing so, it may serve to decrease the likelihood of the processing errors described here.

The model presented in this paper could be adapted to any layer of Mohs surgery, with or without debulking. The conclusions will always be the same. A variety of processing errors can be significantly reduced by taking thin layers, and processing tissue in the least number of blocks possible.

## Conclusion

As previously described, variability exists in the technique of Mohs Surgery. This paper represents the first known attempt to quantitate in a mathematical way the consequence of some components of this variation. Evidence is provided which suggests that minimizing the number of blocks an excised layer is cut into when processing, and minimizing the thickness or depth of an excised layer can dramatically improve the cure rate of Mohs Surgery.

## Abbreviations

Aside = Area Side

r = r_1 _+ r_2 _= radius of Abase

r_1 _= length of base

r_2 _= length of side wall

N = Number of blocks

d = depth (thickness)

## Competing interests

The author(s) declare that they have no competing interests.

## Authors' contributions

DMS conceived of the study, and participated in its design and coordination. JIE, TK, AW and DMS designed the mathematical proof. JIE created the clay animations, and statistical analysis. MOG helped in the modeling and animations. All authors read and approved the final manuscript.

## Pre-publication history

The pre-publication history for this paper can be accessed here:



## Supplementary Material

Additional File 1 Optimal tissue processing. Power point animation of optimal tissue processing Click here for file

Additional File 2 Edge lift error. Power point animation of an edge lift error Click here for file

Additional File 3 Squash error. Power point animation of a thick layer squash error Click here for file

Additional File 4 Tip lift error. Power point animation of a tip lift error Click here for file

Additional File 5 Thin section collapse error. Power point animation of a thin section collapse error Click here for file
