# Supplementary material for: Mohs math – where the error hides
Source: BMC Dermatol. 2006 Dec 6;6:10. doi: 10.1186/1471-5945-6-10 (PMC1769395; doi:10.1186/1471-5945-6-10)
Supplement: Additional File 1 — Optimal tissue processing. Power point animation of optimal tissue processing [file 1471-5945-6-10-S1.ppt]

## Slide 1
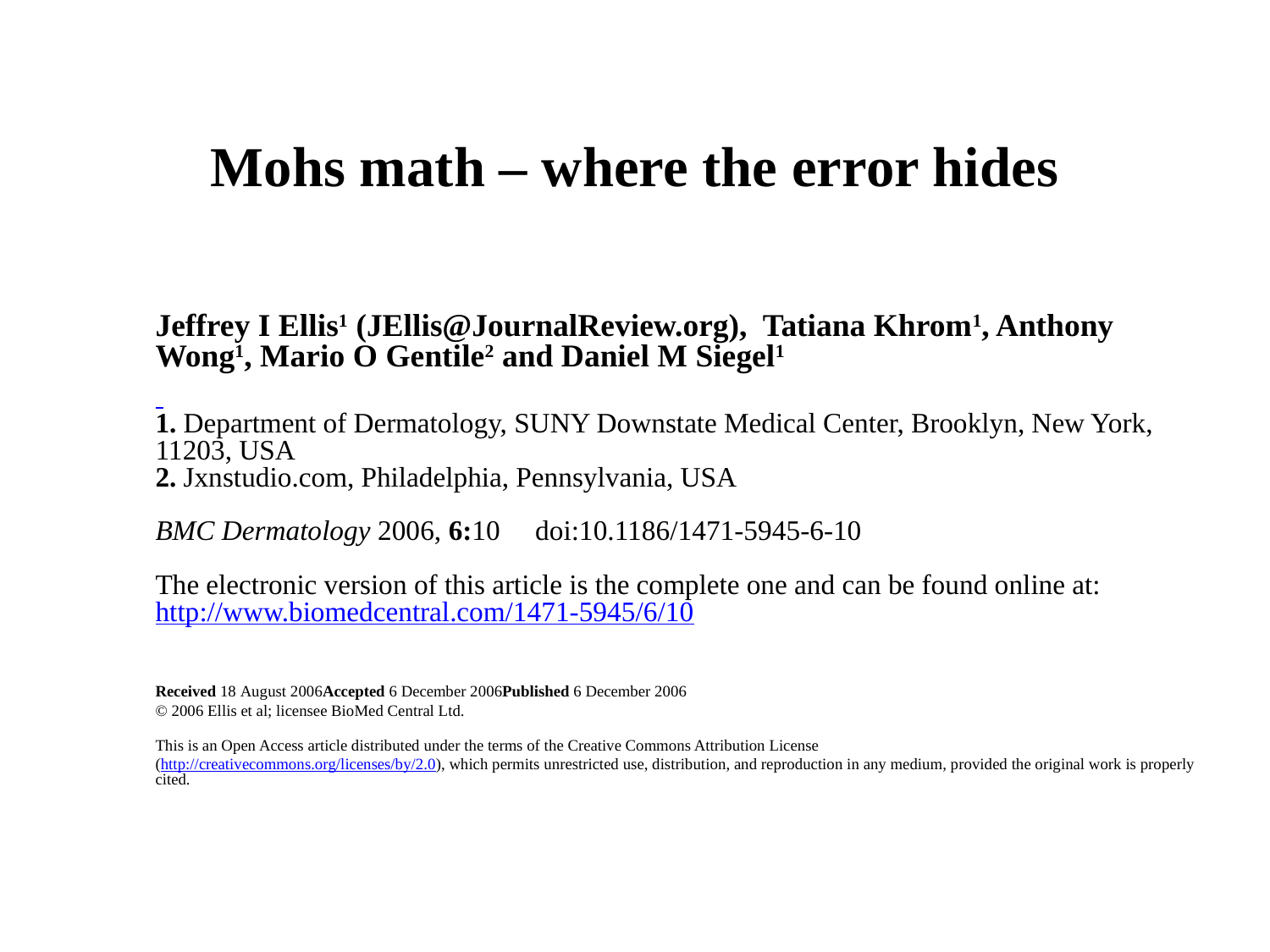

# Mohs math – where the error hides
Jeffrey I Ellis1 (JEllis@JournalReview.org), Tatiana Khrom1, Anthony Wong1, Mario O Gentile2 and Daniel M Siegel1
1. Department of Dermatology, SUNY Downstate Medical Center, Brooklyn, New York, 11203, USA
2. Jxnstudio.com, Philadelphia, Pennsylvania, USA
BMC Dermatology 2006, 6:10     doi:10.1186/1471-5945-6-10
The electronic version of this article is the complete one and can be found online at: http://www.biomedcentral.com/1471-5945/6/10
Received 18 August 2006Accepted 6 December 2006Published 6 December 2006
© 2006 Ellis et al; licensee BioMed Central Ltd.
This is an Open Access article distributed under the terms of the Creative Commons Attribution License
(http://creativecommons.org/licenses/by/2.0), which permits unrestricted use, distribution, and reproduction in any medium, provided the original work is properly cited.

## Slide 2
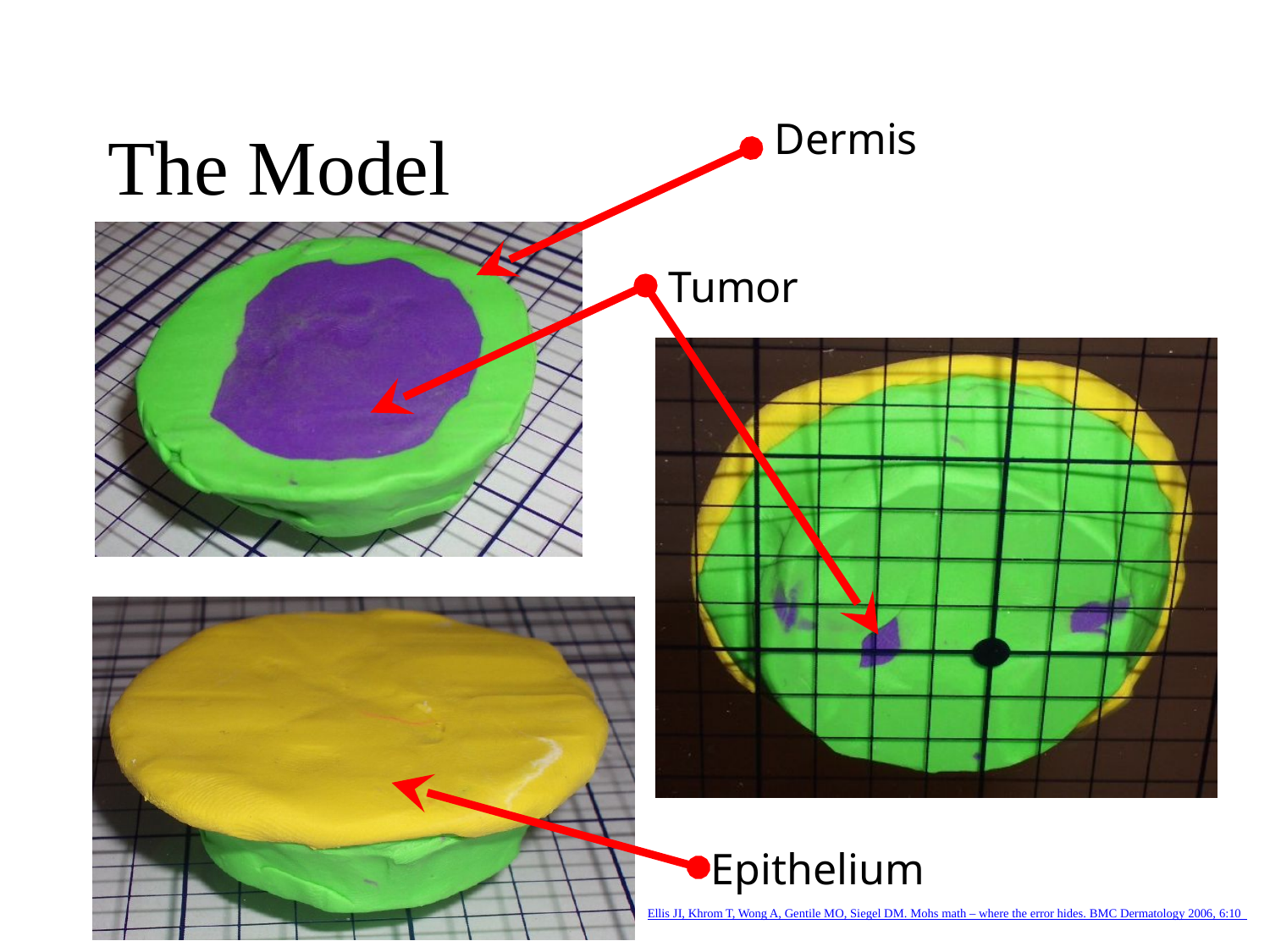

# The Model
Dermis
Tumor
Epithelium
Ellis JI, Khrom T, Wong A, Gentile MO, Siegel DM. Mohs math – where the error hides. BMC Dermatology 2006, 6:10

## Slide 3
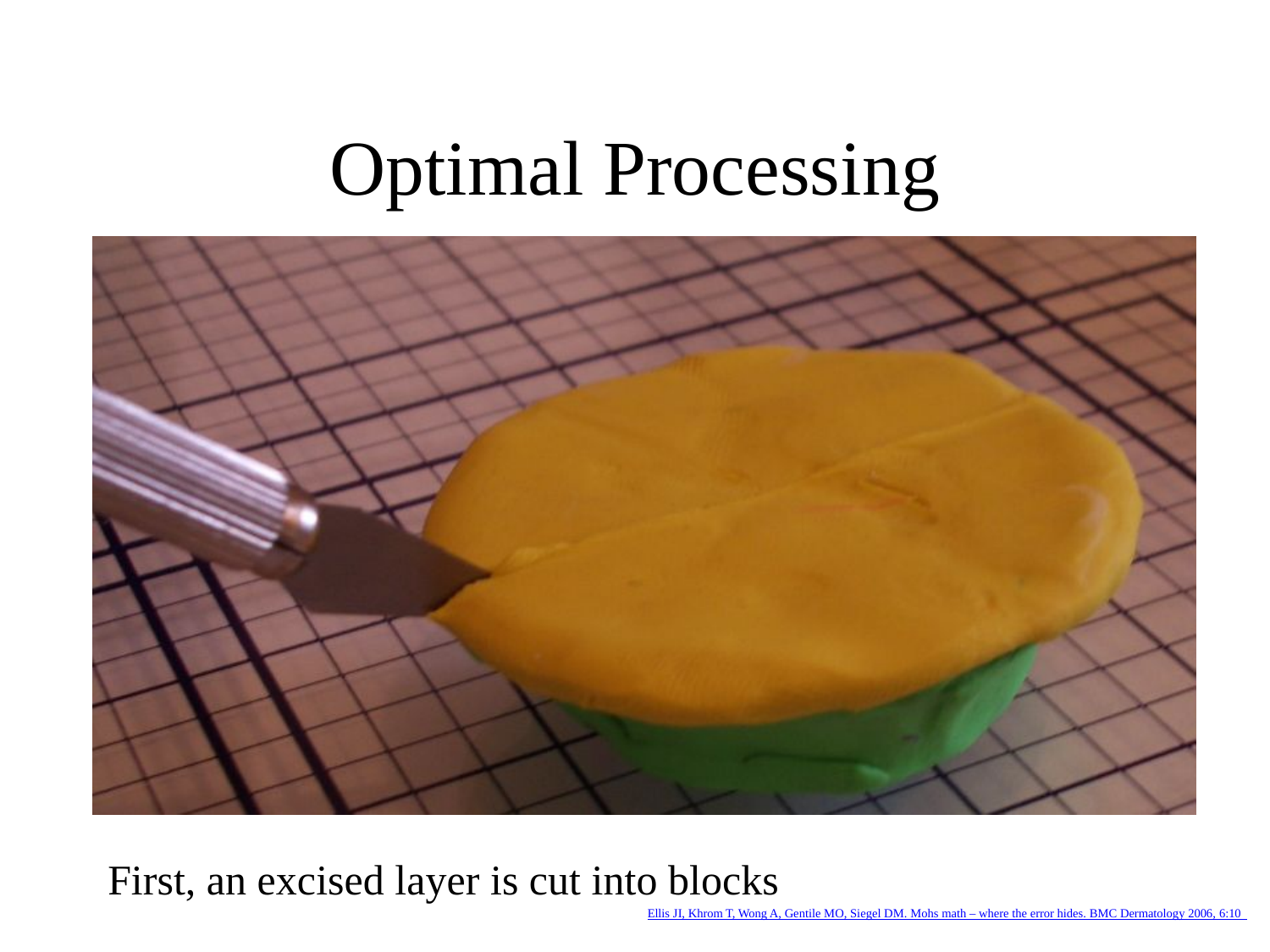

# Optimal Processing
First, an excised layer is cut into blocks
Ellis JI, Khrom T, Wong A, Gentile MO, Siegel DM. Mohs math – where the error hides. BMC Dermatology 2006, 6:10

## Slide 4
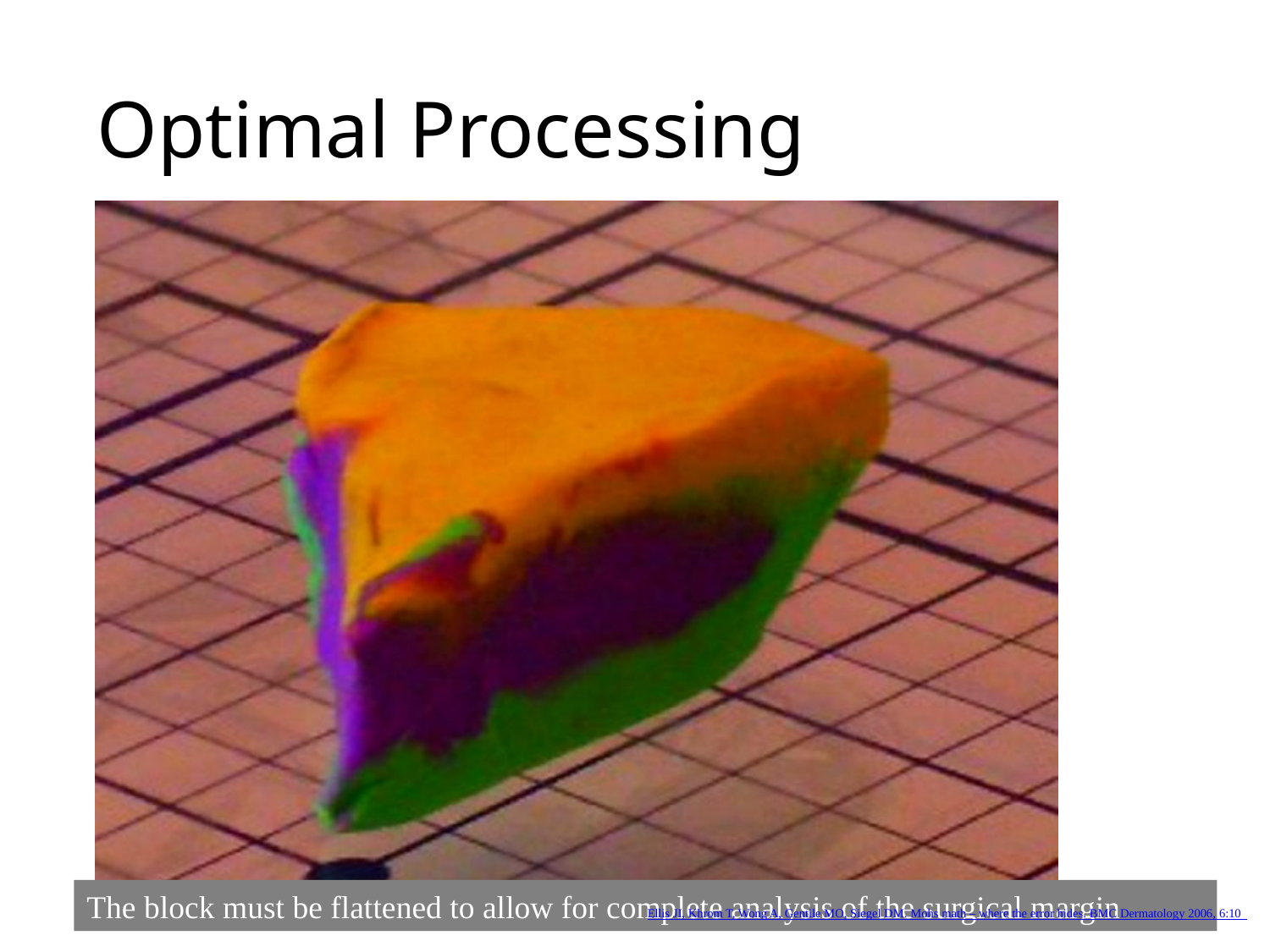

Optimal Processing
The block must be flattened to allow for complete analysis of the surgical margin
Ellis JI, Khrom T, Wong A, Gentile MO, Siegel DM. Mohs math – where the error hides. BMC Dermatology 2006, 6:10

## Slide 5
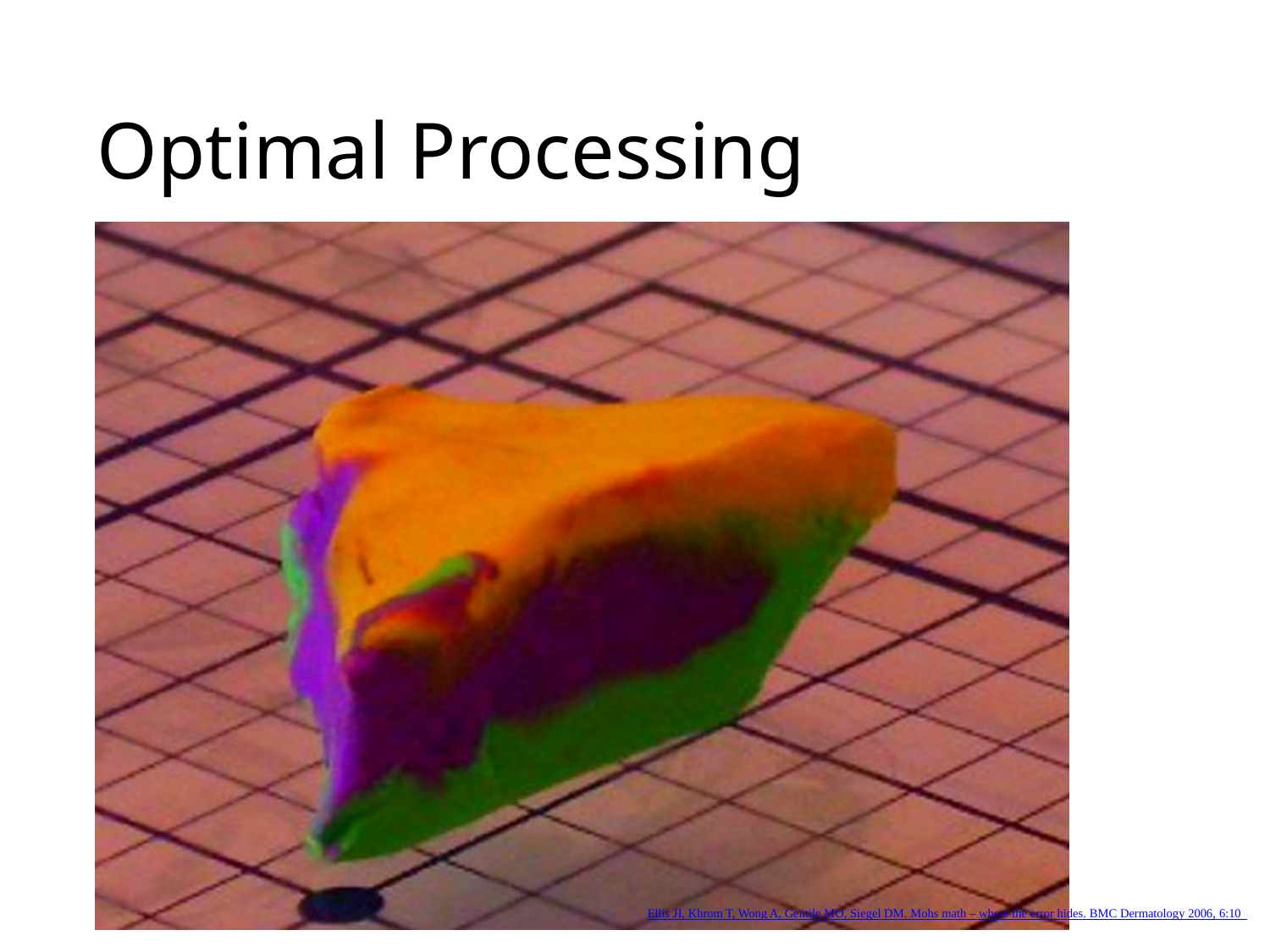

Optimal Processing
Ellis JI, Khrom T, Wong A, Gentile MO, Siegel DM. Mohs math – where the error hides. BMC Dermatology 2006, 6:10

## Slide 6
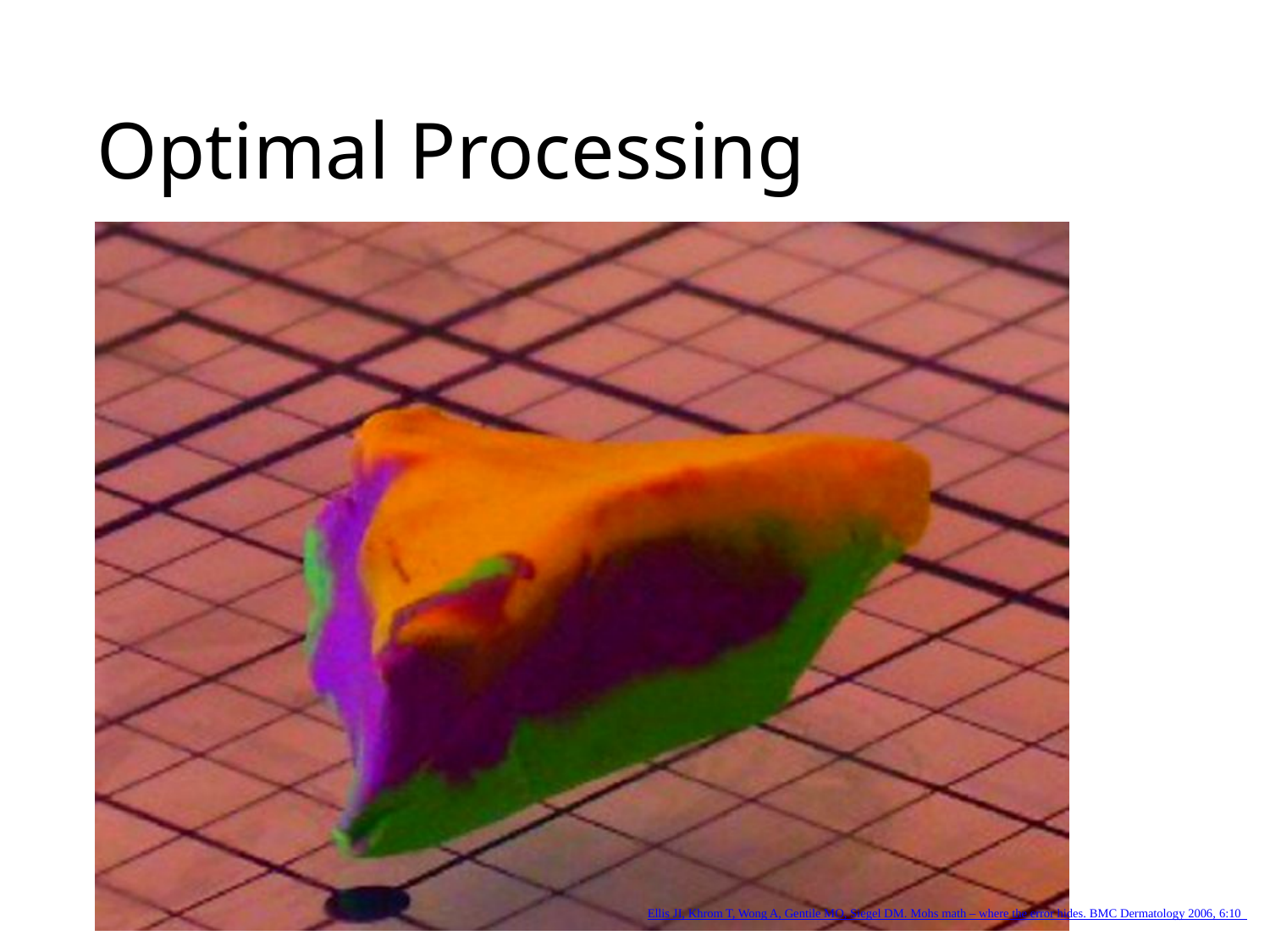

Optimal Processing
Ellis JI, Khrom T, Wong A, Gentile MO, Siegel DM. Mohs math – where the error hides. BMC Dermatology 2006, 6:10

## Slide 7
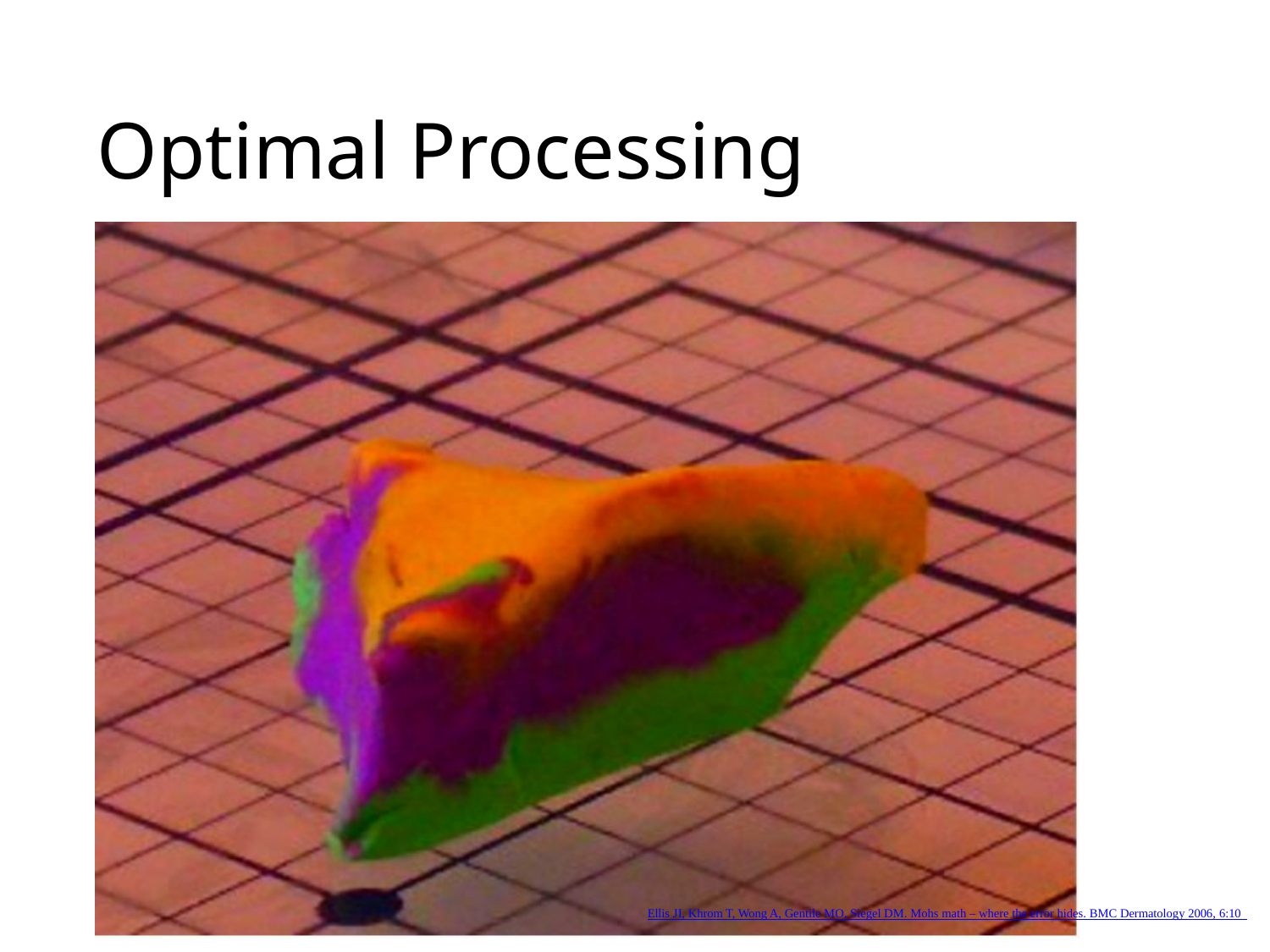

Optimal Processing
Ellis JI, Khrom T, Wong A, Gentile MO, Siegel DM. Mohs math – where the error hides. BMC Dermatology 2006, 6:10

## Slide 8
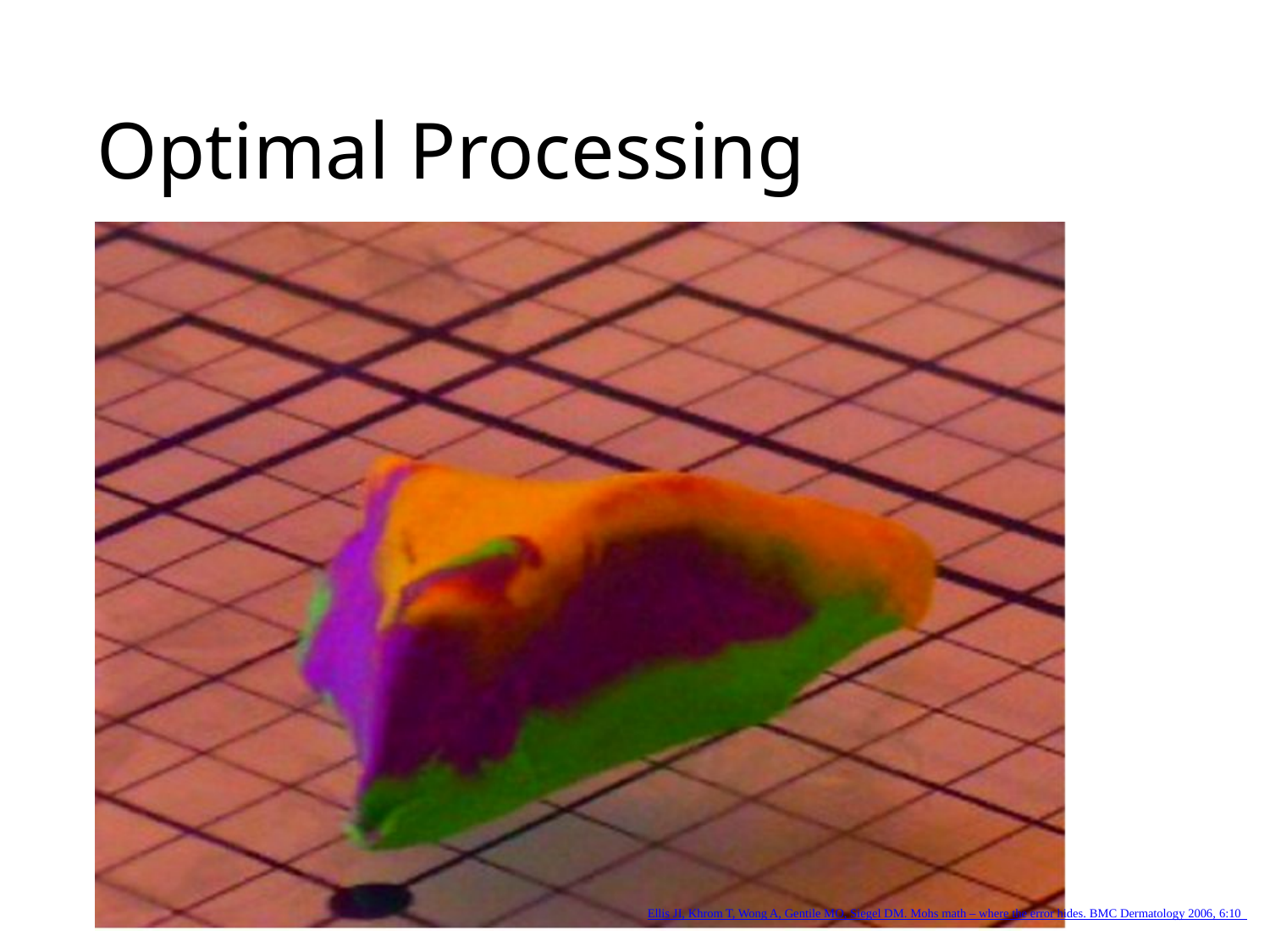

Optimal Processing
Ellis JI, Khrom T, Wong A, Gentile MO, Siegel DM. Mohs math – where the error hides. BMC Dermatology 2006, 6:10

## Slide 9
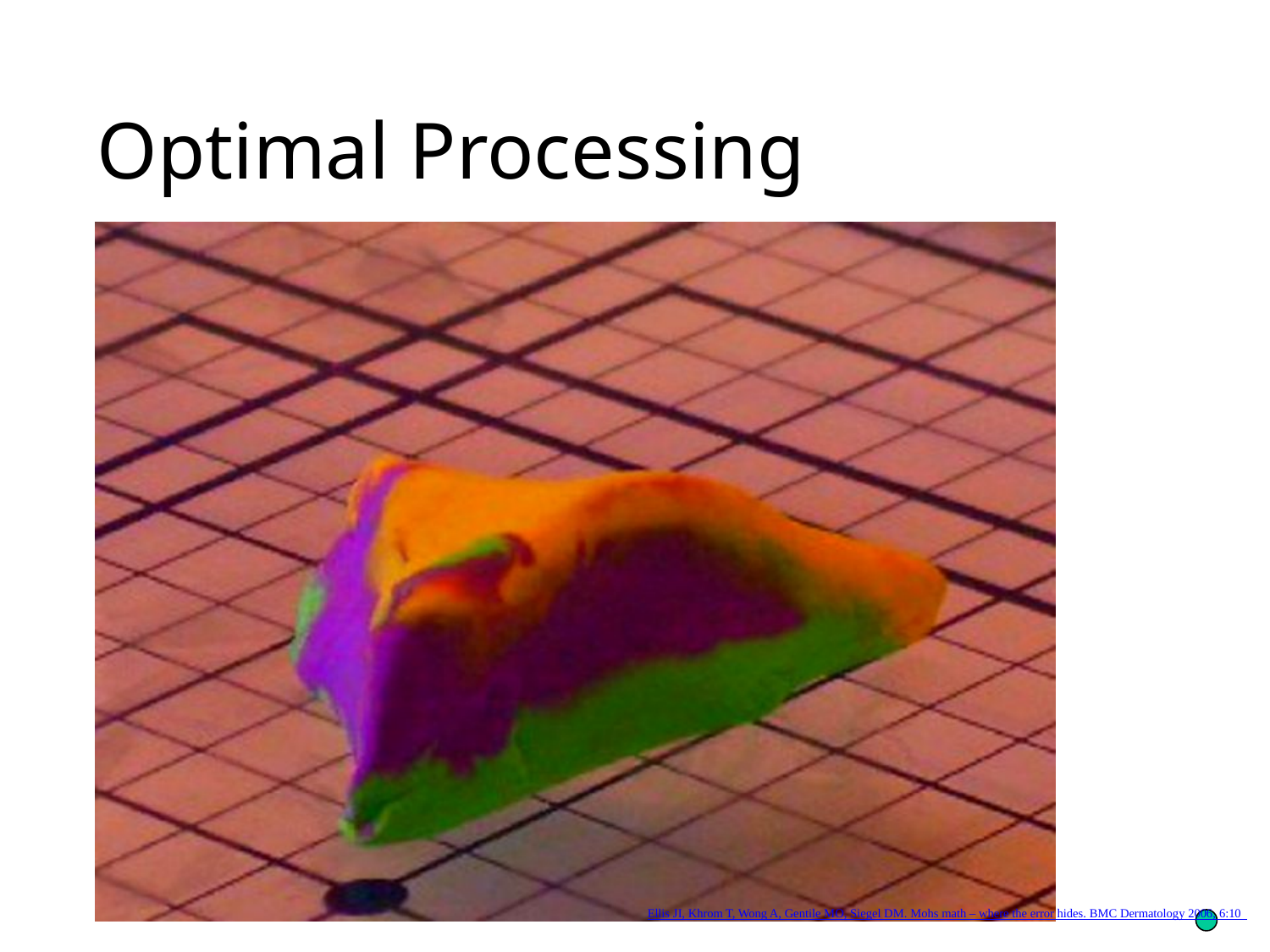

Optimal Processing
Ellis JI, Khrom T, Wong A, Gentile MO, Siegel DM. Mohs math – where the error hides. BMC Dermatology 2006, 6:10

## Slide 10
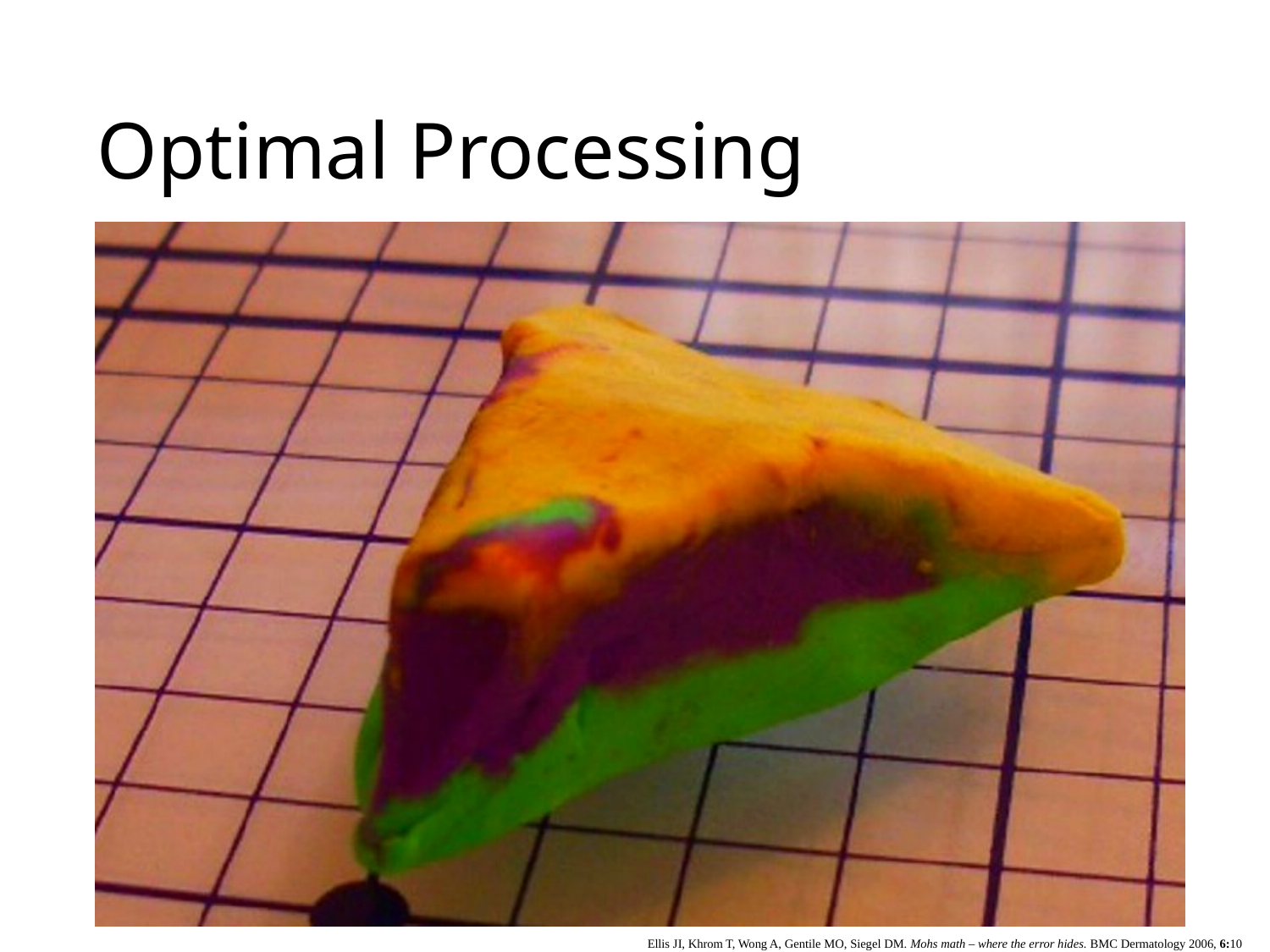

Optimal Processing
Ellis JI, Khrom T, Wong A, Gentile MO, Siegel DM. Mohs math – where the error hides. BMC Dermatology 2006, 6:10

## Slide 11
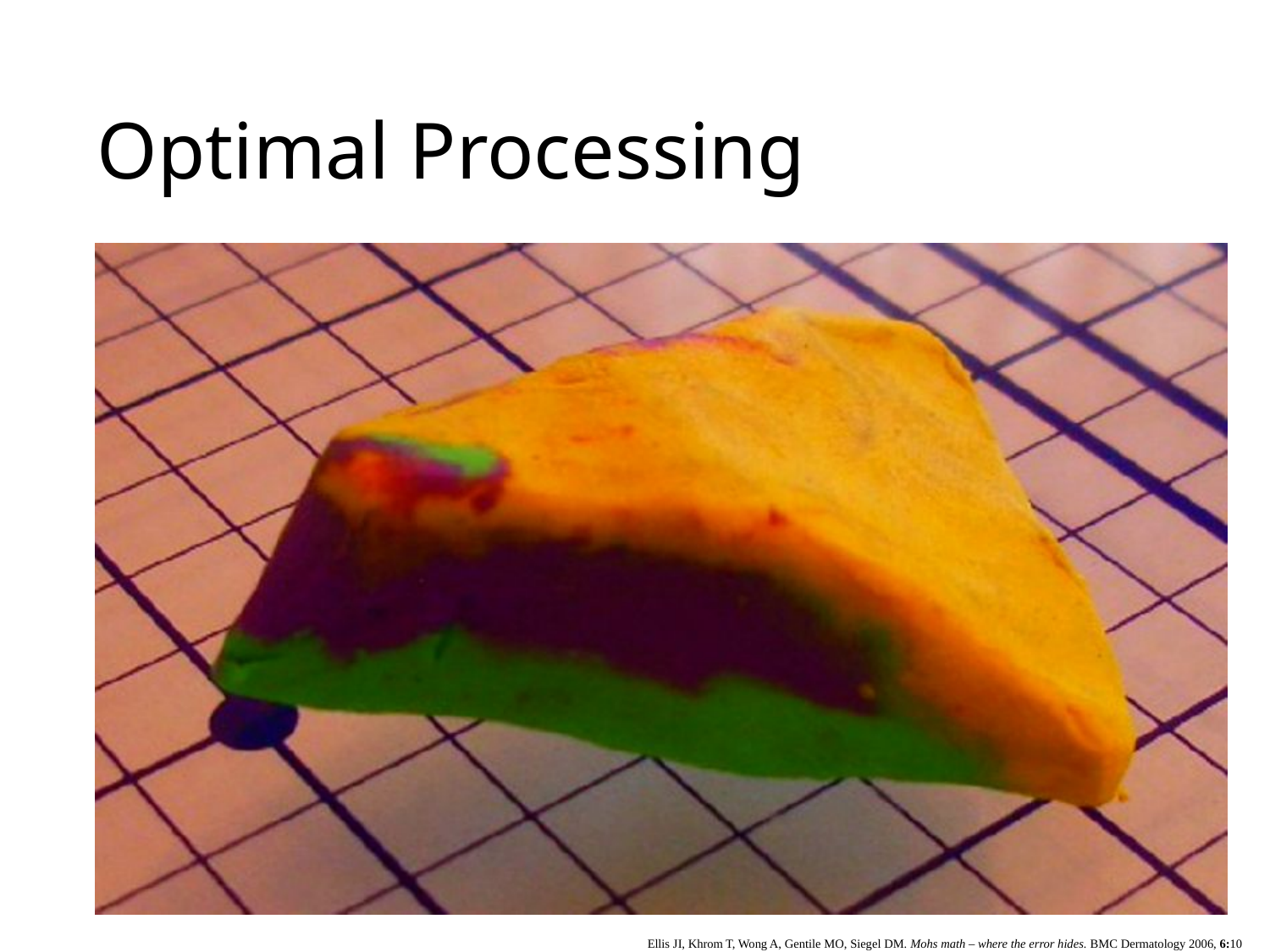

Optimal Processing
Ellis JI, Khrom T, Wong A, Gentile MO, Siegel DM. Mohs math – where the error hides. BMC Dermatology 2006, 6:10

## Slide 12
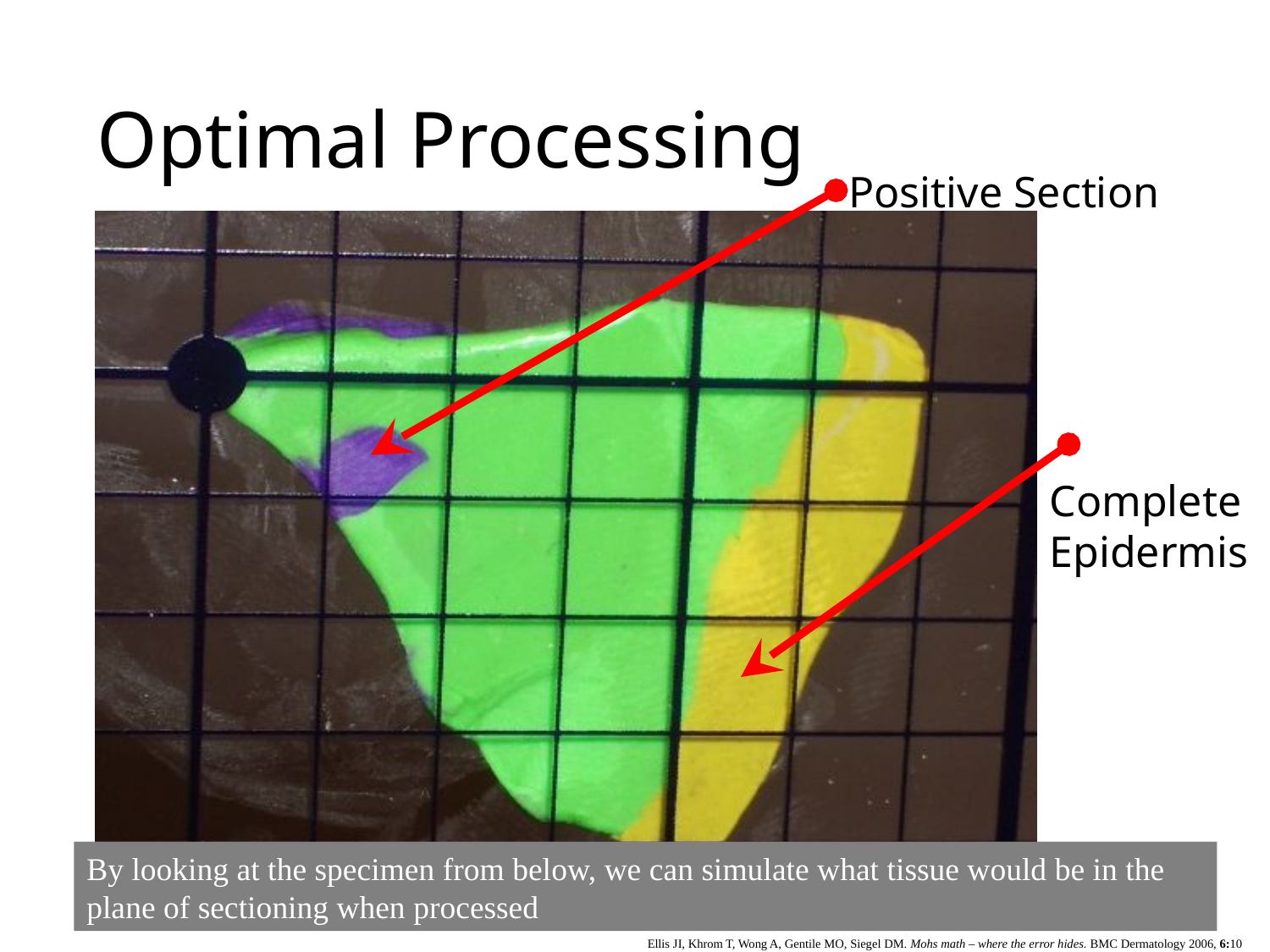

Optimal Processing
Positive Section
Complete Epidermis
By looking at the specimen from below, we can simulate what tissue would be in the plane of sectioning when processed
Ellis JI, Khrom T, Wong A, Gentile MO, Siegel DM. Mohs math – where the error hides. BMC Dermatology 2006, 6:10
