# Supplementary material for: Mohs math – where the error hides
Source: BMC Dermatol. 2006 Dec 6;6:10. doi: 10.1186/1471-5945-6-10 (PMC1769395; doi:10.1186/1471-5945-6-10)
Supplement: Additional File 2 — Edge lift error. Power point animation of an edge lift error [file 1471-5945-6-10-S2.ppt]

## Slide 1
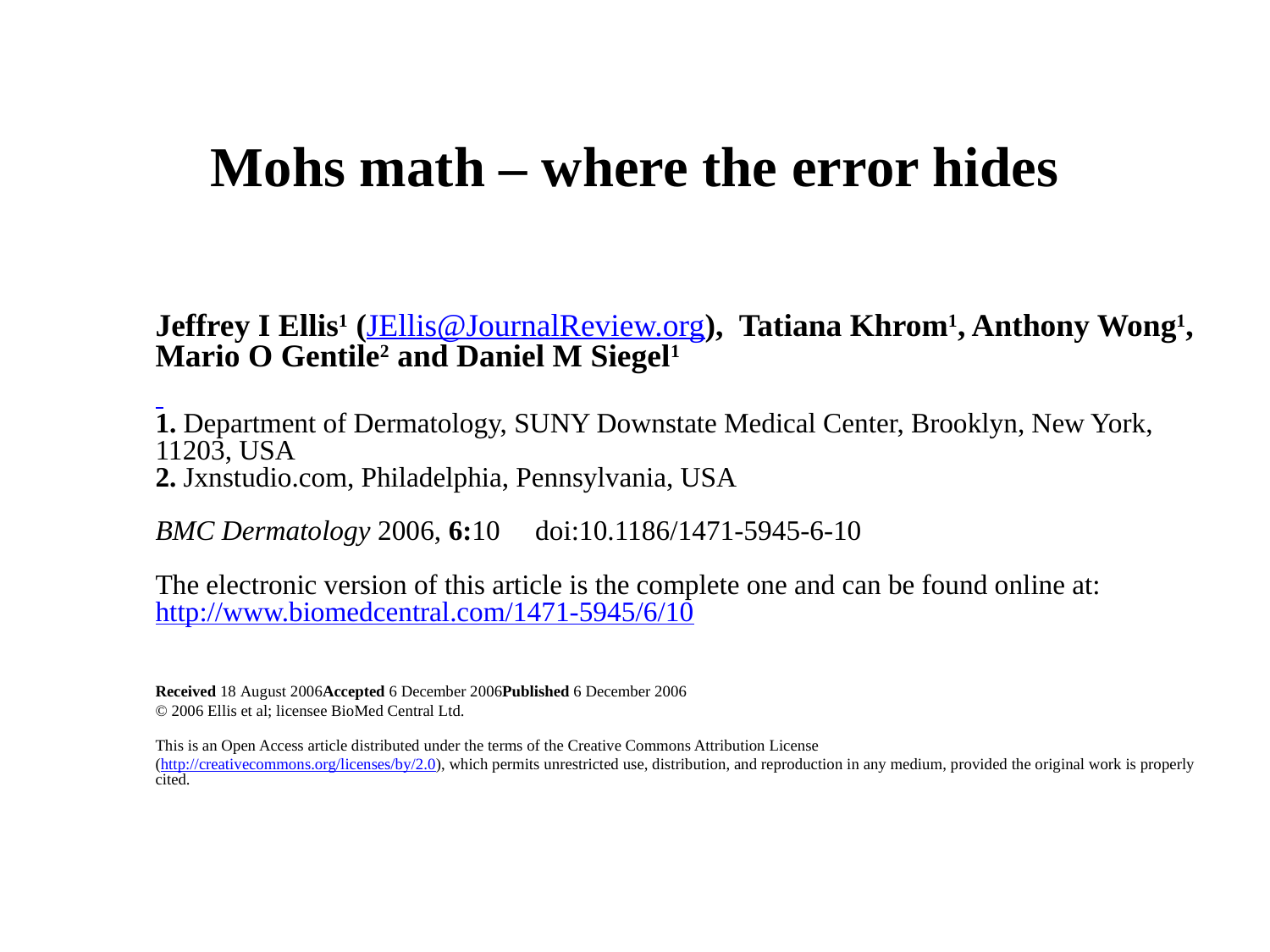

# Mohs math – where the error hides
Jeffrey I Ellis1 (JEllis@JournalReview.org), Tatiana Khrom1, Anthony Wong1, Mario O Gentile2 and Daniel M Siegel1
1. Department of Dermatology, SUNY Downstate Medical Center, Brooklyn, New York, 11203, USA
2. Jxnstudio.com, Philadelphia, Pennsylvania, USA
BMC Dermatology 2006, 6:10     doi:10.1186/1471-5945-6-10
The electronic version of this article is the complete one and can be found online at: http://www.biomedcentral.com/1471-5945/6/10
Received 18 August 2006Accepted 6 December 2006Published 6 December 2006
© 2006 Ellis et al; licensee BioMed Central Ltd.
This is an Open Access article distributed under the terms of the Creative Commons Attribution License
(http://creativecommons.org/licenses/by/2.0), which permits unrestricted use, distribution, and reproduction in any medium, provided the original work is properly cited.

## Slide 2
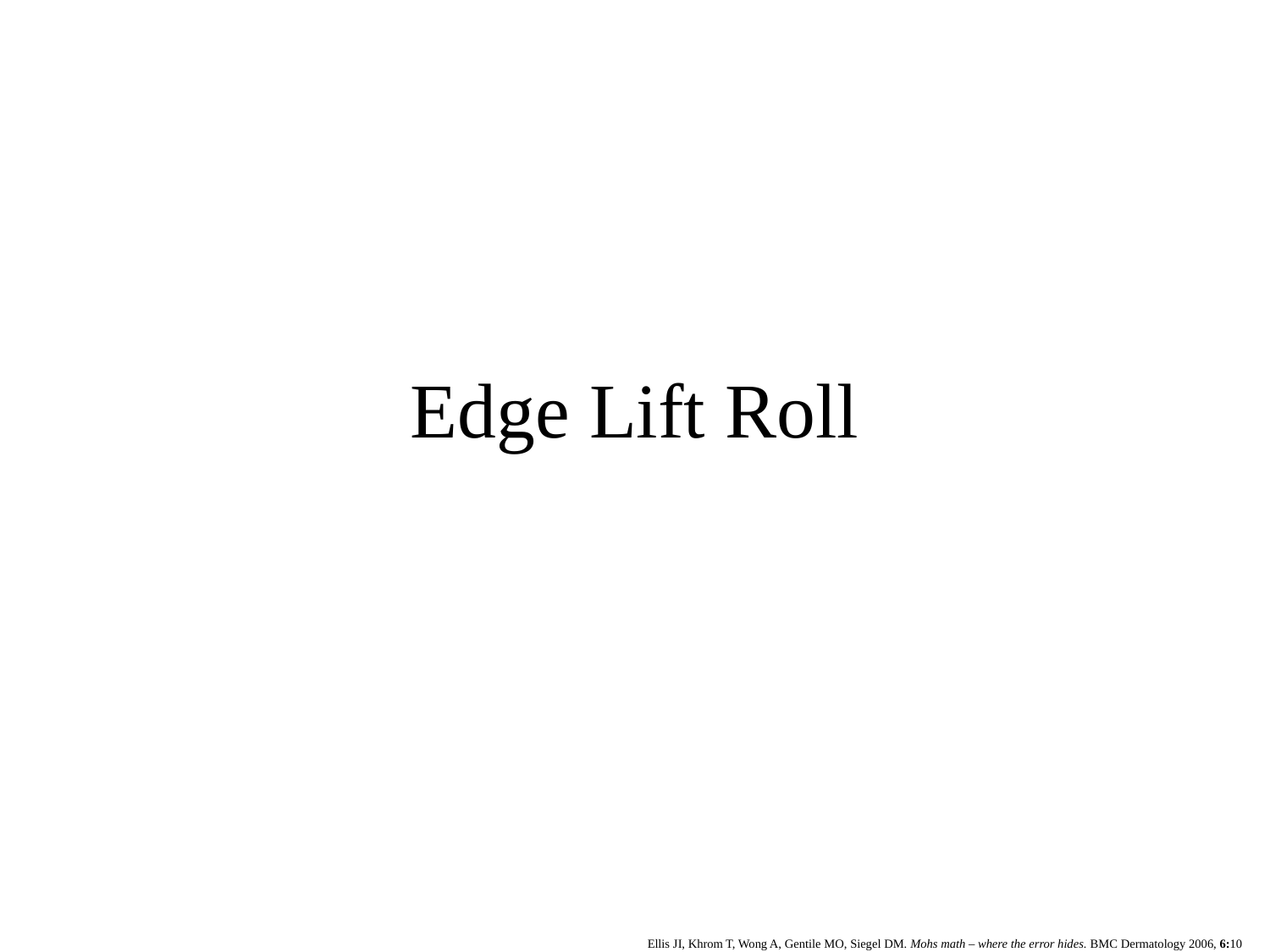

# Edge Lift Roll
Ellis JI, Khrom T, Wong A, Gentile MO, Siegel DM. Mohs math – where the error hides. BMC Dermatology 2006, 6:10

## Slide 3
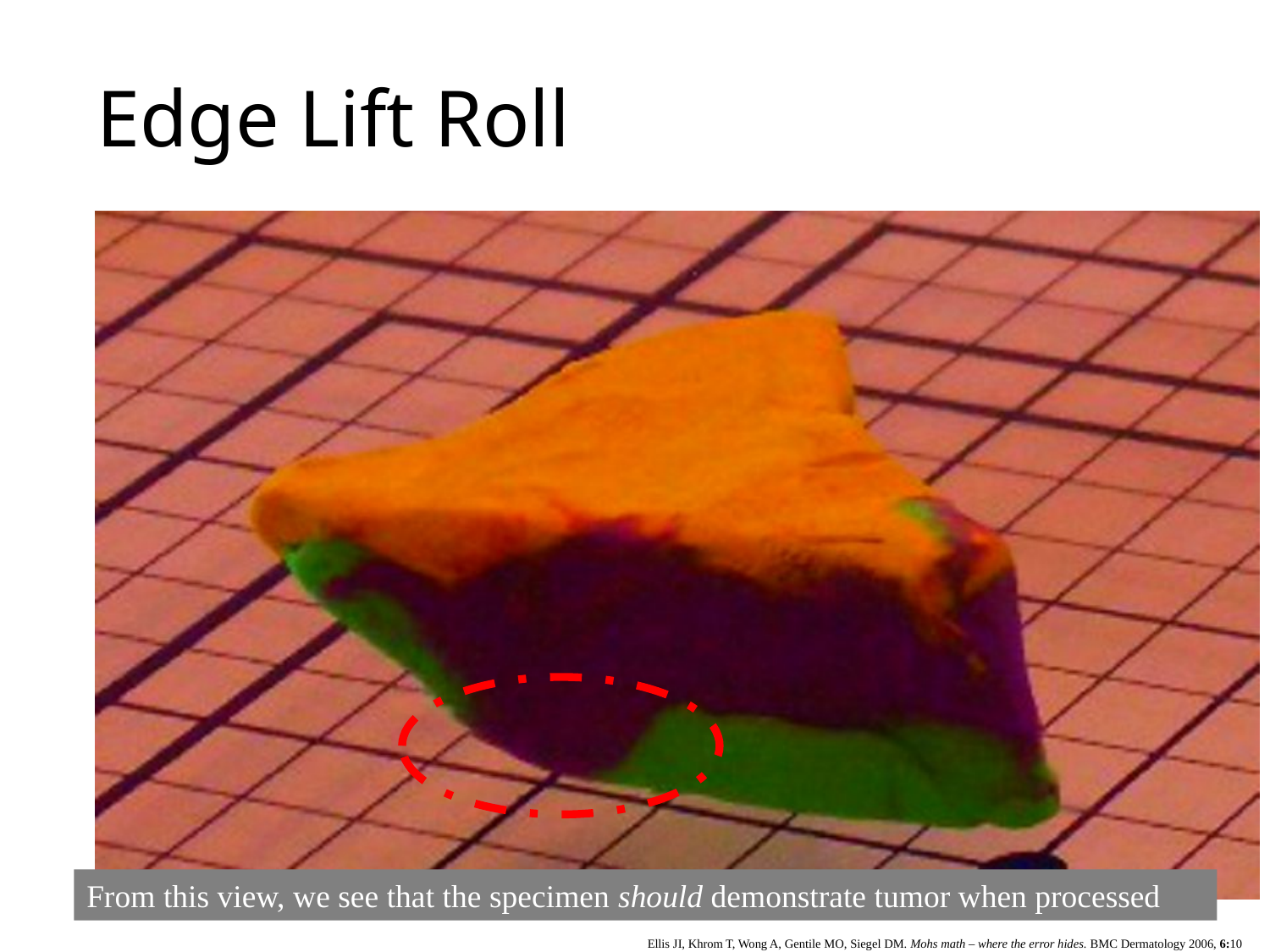

Edge Lift Roll
From this view, we see that the specimen should demonstrate tumor when processed
Ellis JI, Khrom T, Wong A, Gentile MO, Siegel DM. Mohs math – where the error hides. BMC Dermatology 2006, 6:10

## Slide 4
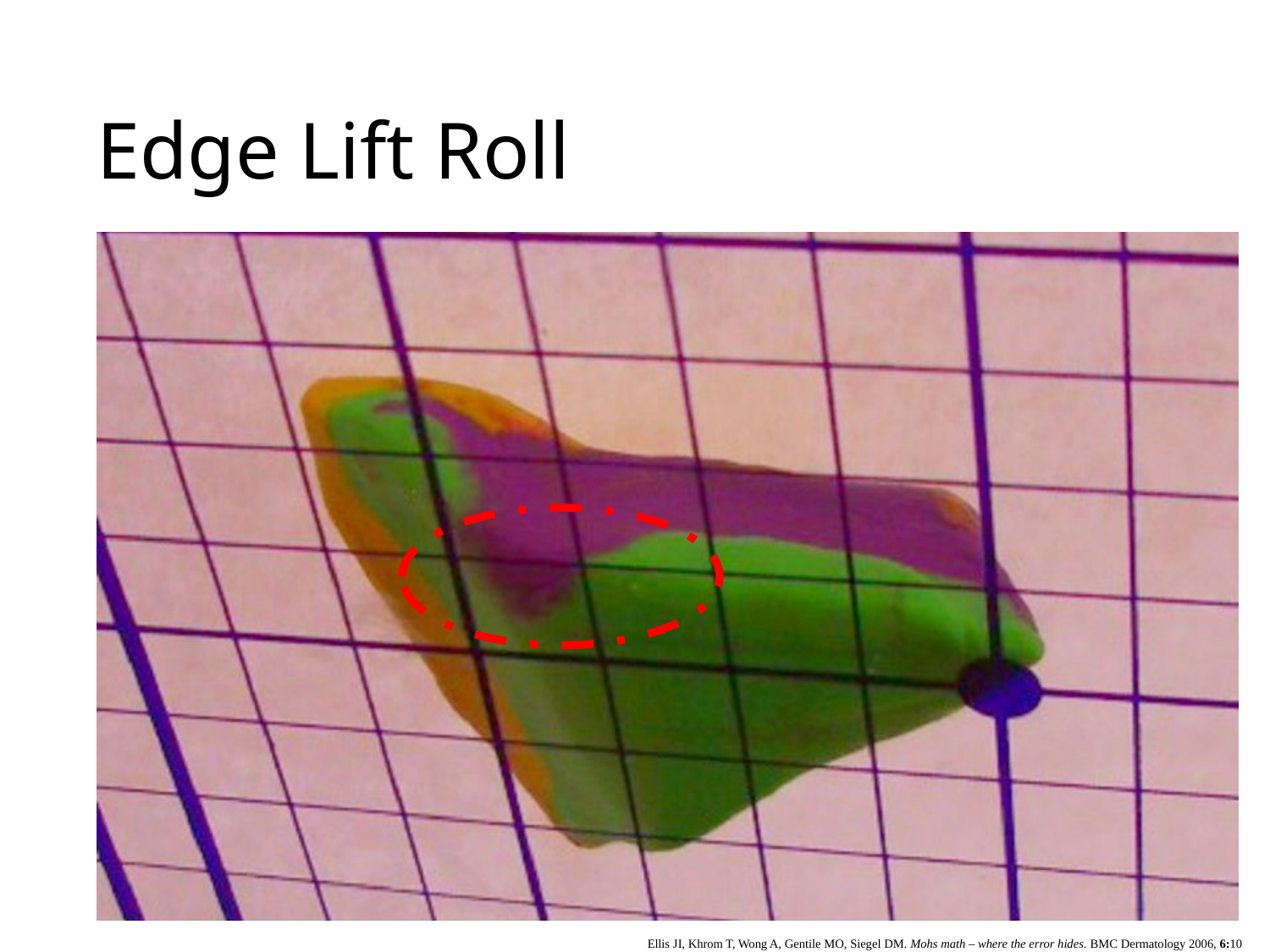

Edge Lift Roll
Ellis JI, Khrom T, Wong A, Gentile MO, Siegel DM. Mohs math – where the error hides. BMC Dermatology 2006, 6:10

## Slide 5
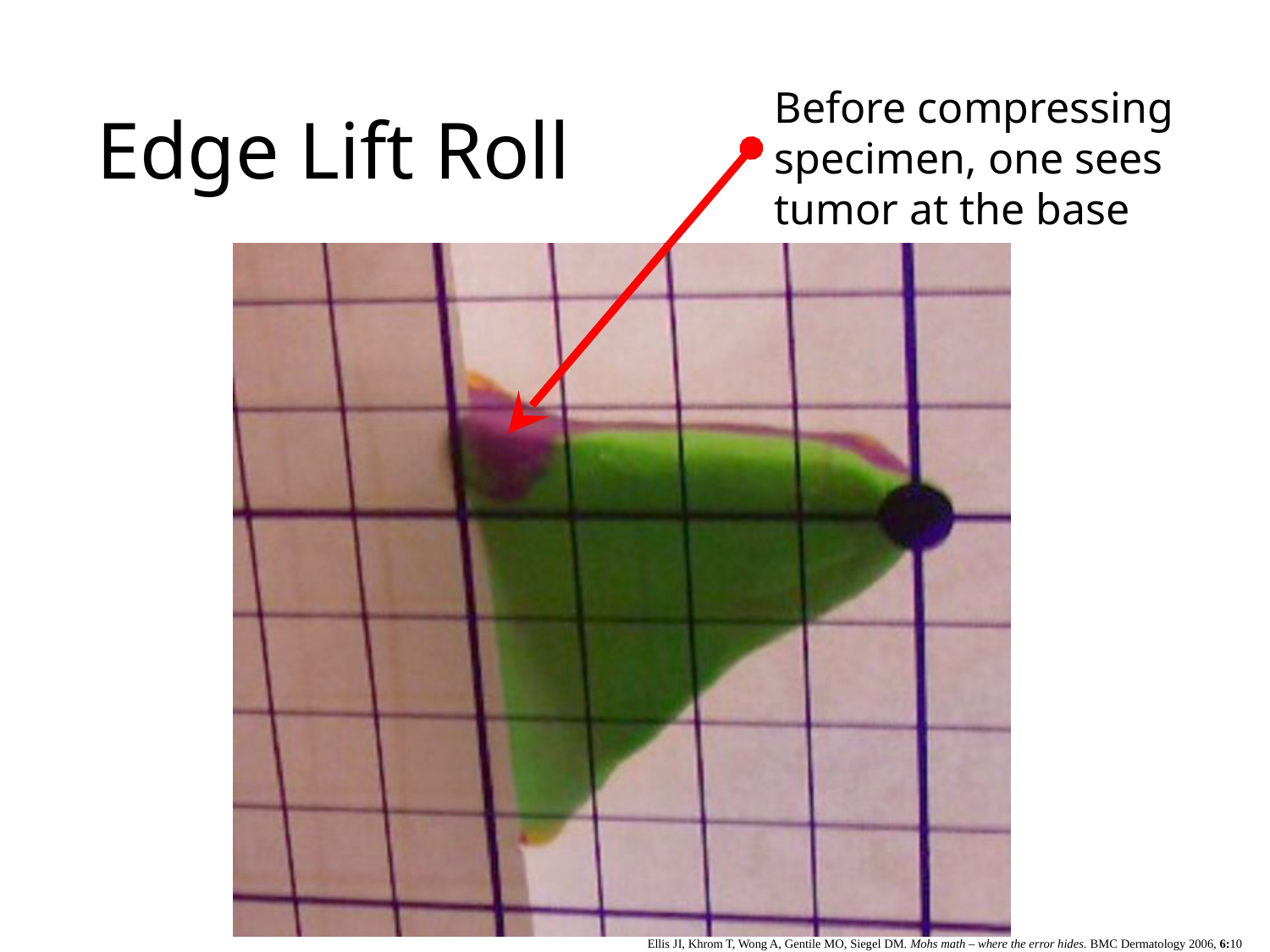

Edge Lift Roll
Before compressing specimen, one sees tumor at the base
Ellis JI, Khrom T, Wong A, Gentile MO, Siegel DM. Mohs math – where the error hides. BMC Dermatology 2006, 6:10

## Slide 6
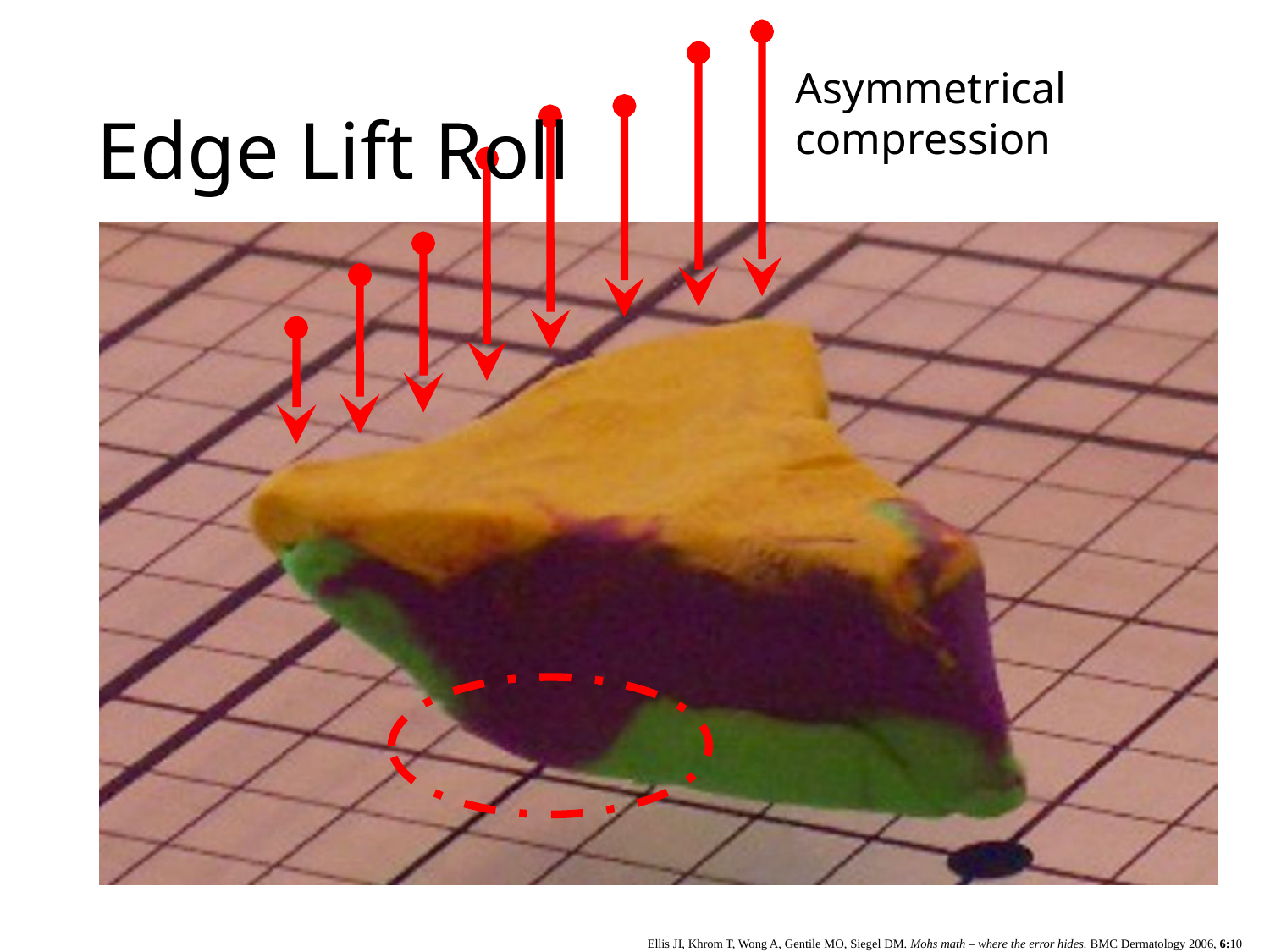

Edge Lift Roll
Asymmetrical compression
Ellis JI, Khrom T, Wong A, Gentile MO, Siegel DM. Mohs math – where the error hides. BMC Dermatology 2006, 6:10

## Slide 7
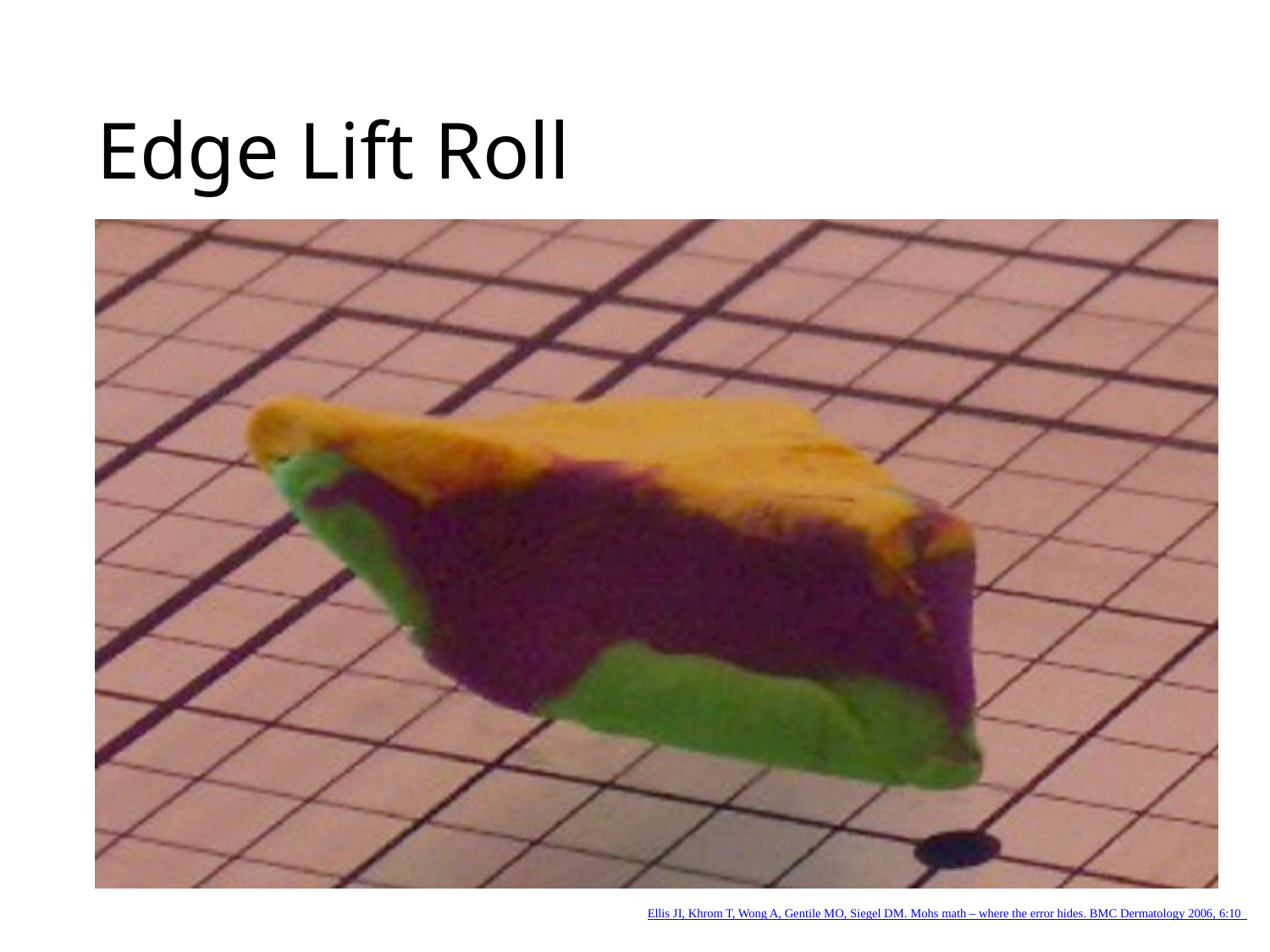

Edge Lift Roll
Ellis JI, Khrom T, Wong A, Gentile MO, Siegel DM. Mohs math – where the error hides. BMC Dermatology 2006, 6:10

## Slide 8
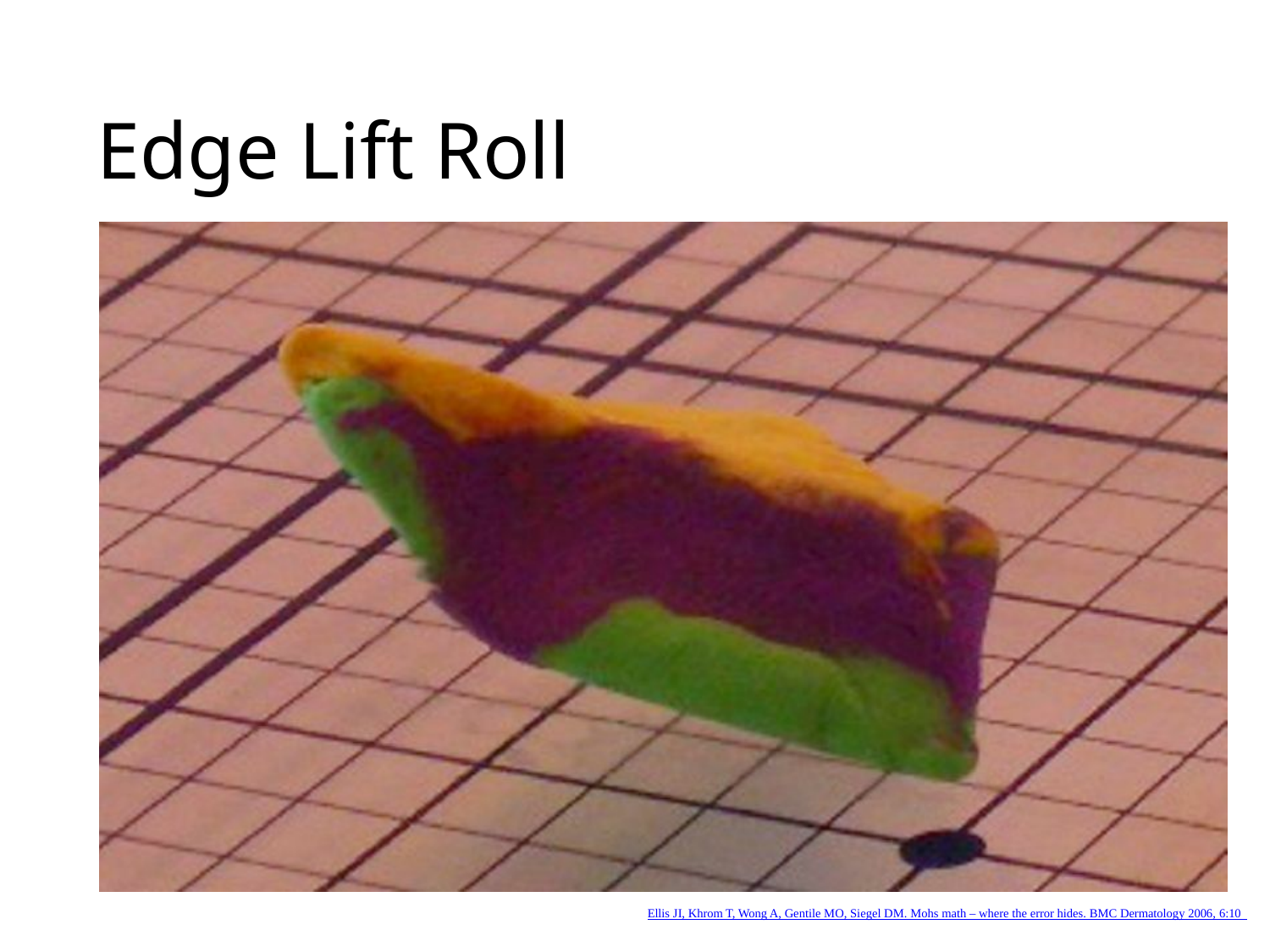

Edge Lift Roll
Ellis JI, Khrom T, Wong A, Gentile MO, Siegel DM. Mohs math – where the error hides. BMC Dermatology 2006, 6:10

## Slide 9
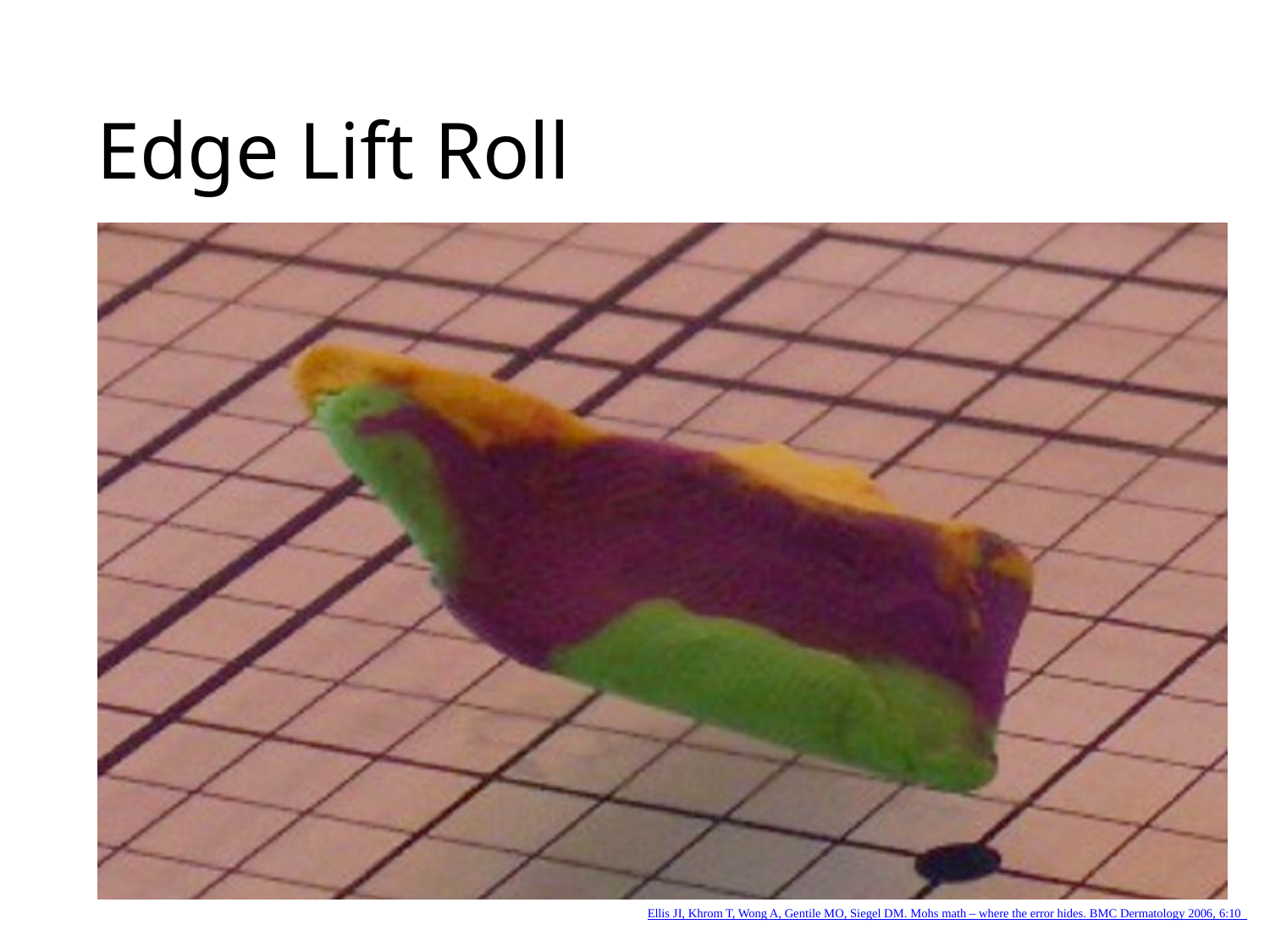

Edge Lift Roll
Ellis JI, Khrom T, Wong A, Gentile MO, Siegel DM. Mohs math – where the error hides. BMC Dermatology 2006, 6:10

## Slide 10
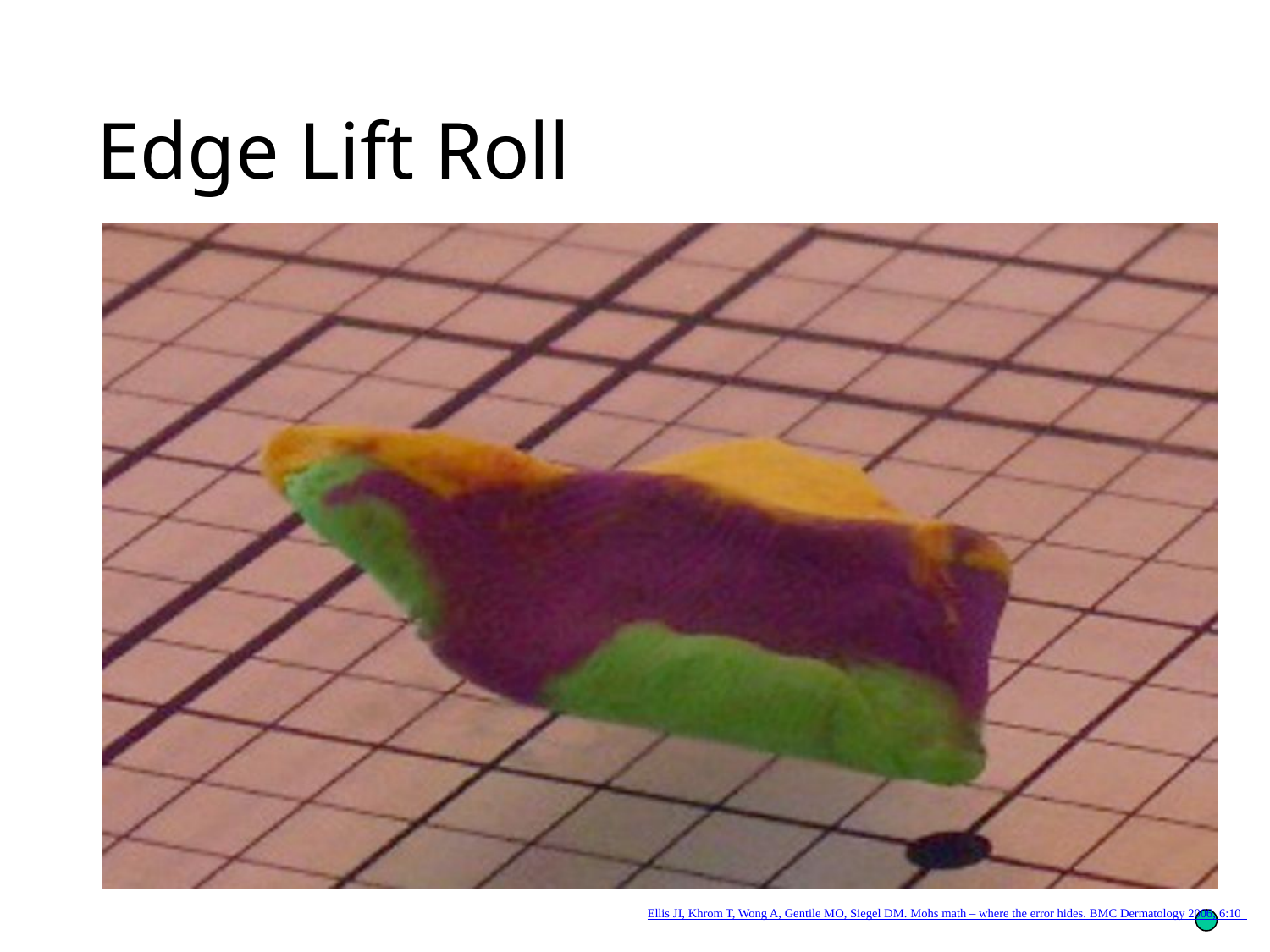

Edge Lift Roll
Ellis JI, Khrom T, Wong A, Gentile MO, Siegel DM. Mohs math – where the error hides. BMC Dermatology 2006, 6:10

## Slide 11
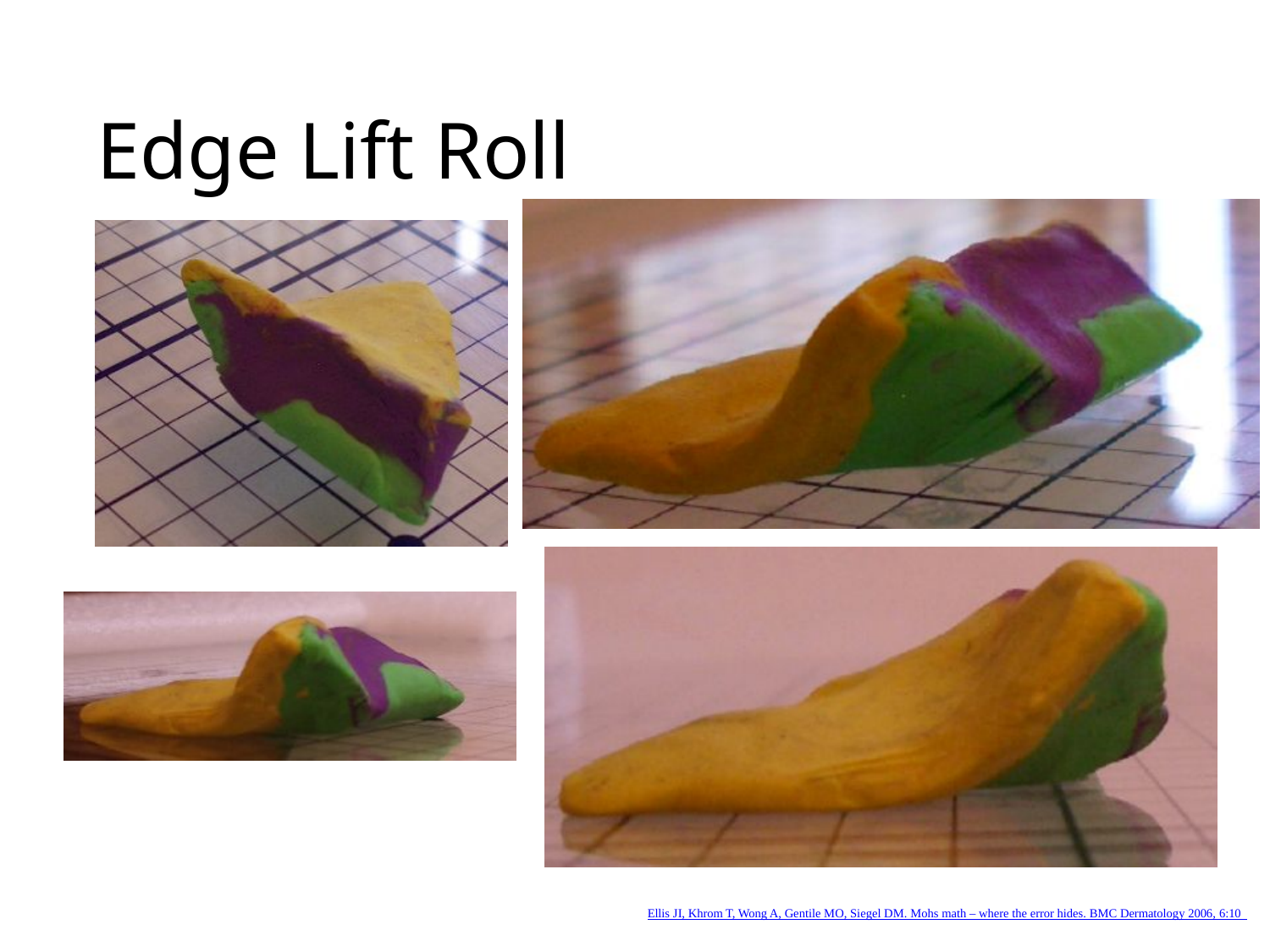

Edge Lift Roll
Ellis JI, Khrom T, Wong A, Gentile MO, Siegel DM. Mohs math – where the error hides. BMC Dermatology 2006, 6:10

## Slide 12
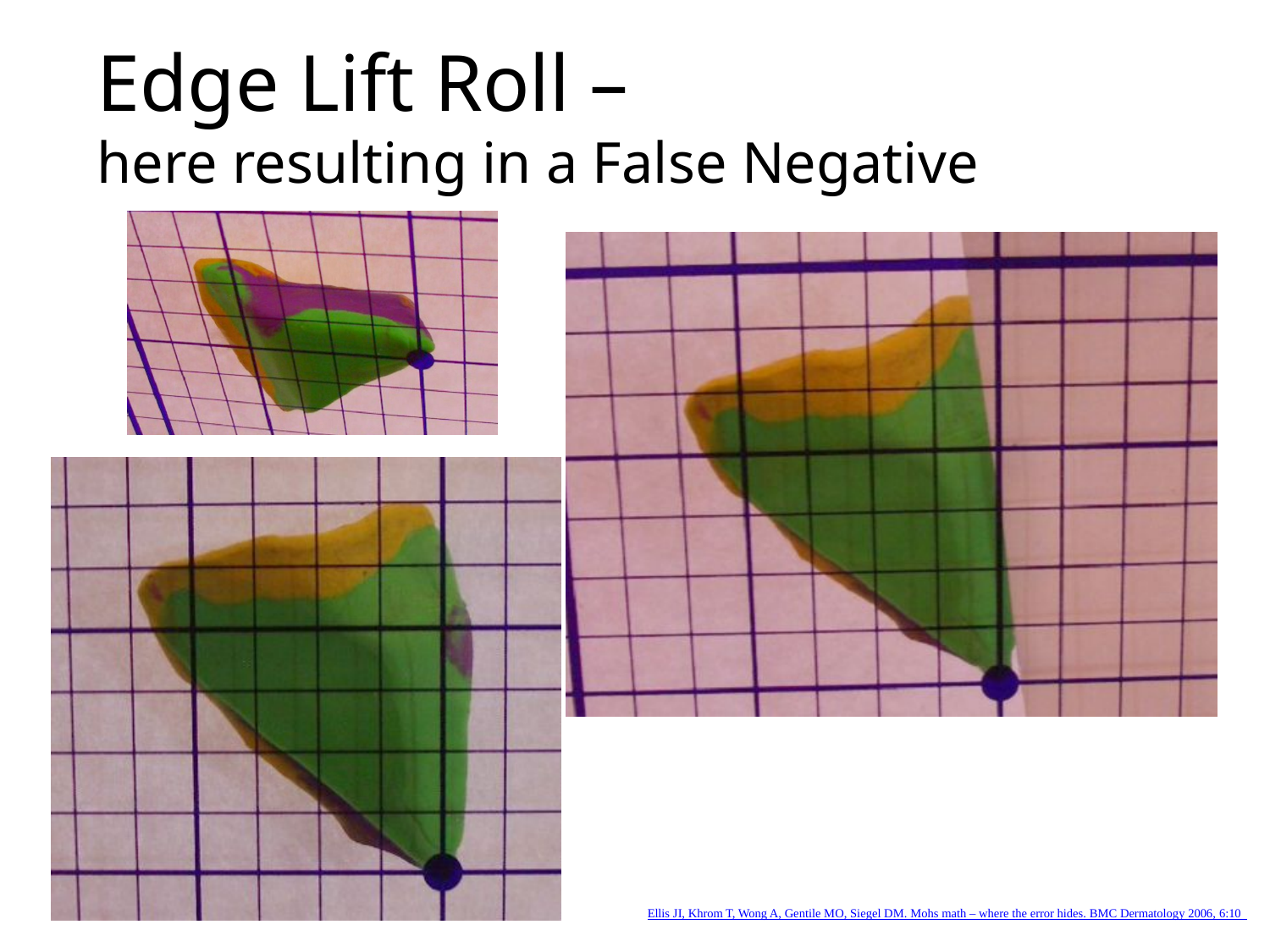

Edge Lift Roll –
here resulting in a False Negative
Ellis JI, Khrom T, Wong A, Gentile MO, Siegel DM. Mohs math – where the error hides. BMC Dermatology 2006, 6:10
