# Supplementary material for: Mohs math – where the error hides
Source: BMC Dermatol. 2006 Dec 6;6:10. doi: 10.1186/1471-5945-6-10 (PMC1769395; doi:10.1186/1471-5945-6-10)
Supplement: Additional File 3 — Squash error. Power point animation of a thick layer squash error [file 1471-5945-6-10-S3.ppt]

## Slide 1
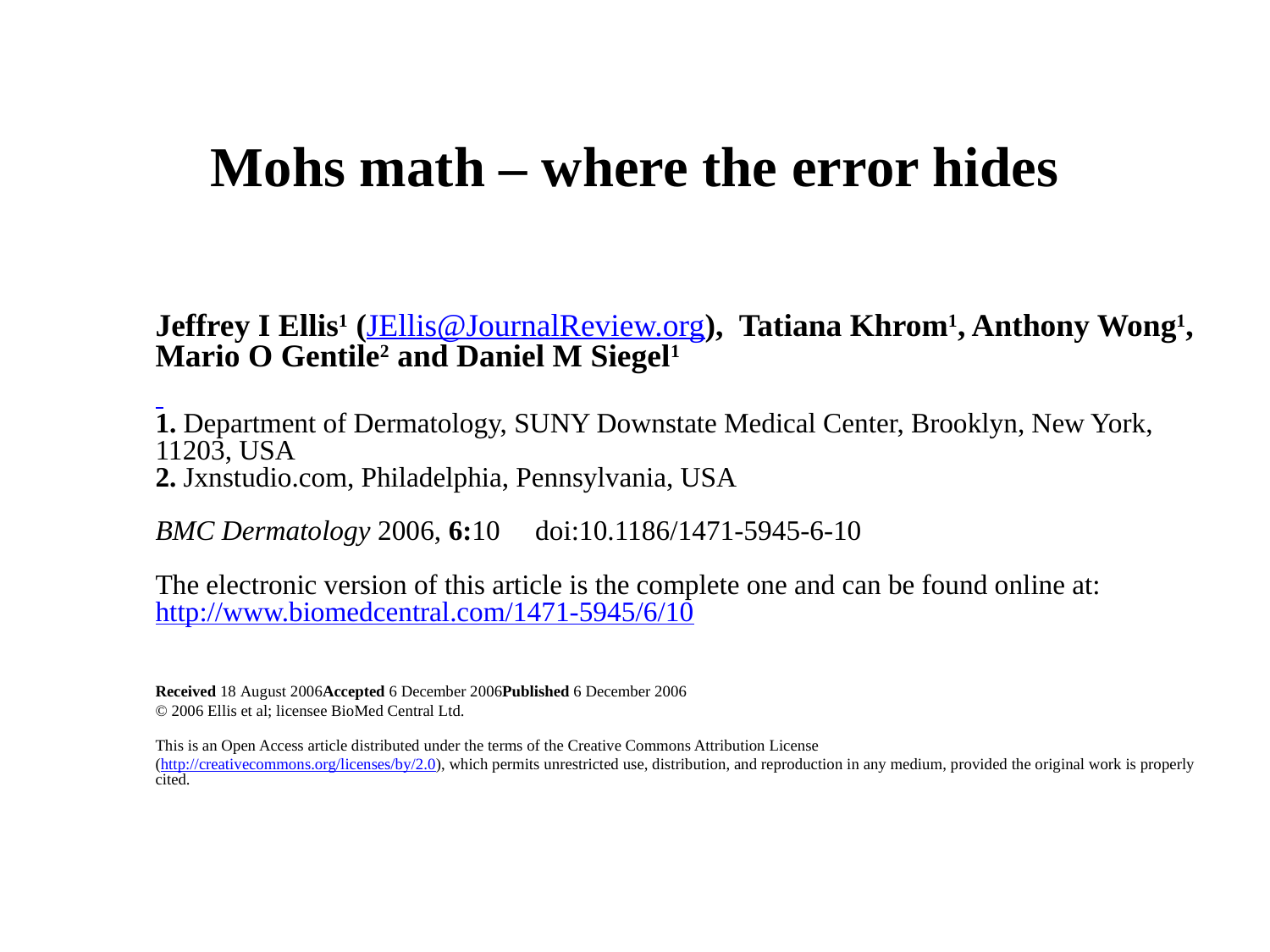

# Mohs math – where the error hides
Jeffrey I Ellis1 (JEllis@JournalReview.org), Tatiana Khrom1, Anthony Wong1, Mario O Gentile2 and Daniel M Siegel1
1. Department of Dermatology, SUNY Downstate Medical Center, Brooklyn, New York, 11203, USA
2. Jxnstudio.com, Philadelphia, Pennsylvania, USA
BMC Dermatology 2006, 6:10     doi:10.1186/1471-5945-6-10
The electronic version of this article is the complete one and can be found online at: http://www.biomedcentral.com/1471-5945/6/10
Received 18 August 2006Accepted 6 December 2006Published 6 December 2006
© 2006 Ellis et al; licensee BioMed Central Ltd.
This is an Open Access article distributed under the terms of the Creative Commons Attribution License
(http://creativecommons.org/licenses/by/2.0), which permits unrestricted use, distribution, and reproduction in any medium, provided the original work is properly cited.

## Slide 2
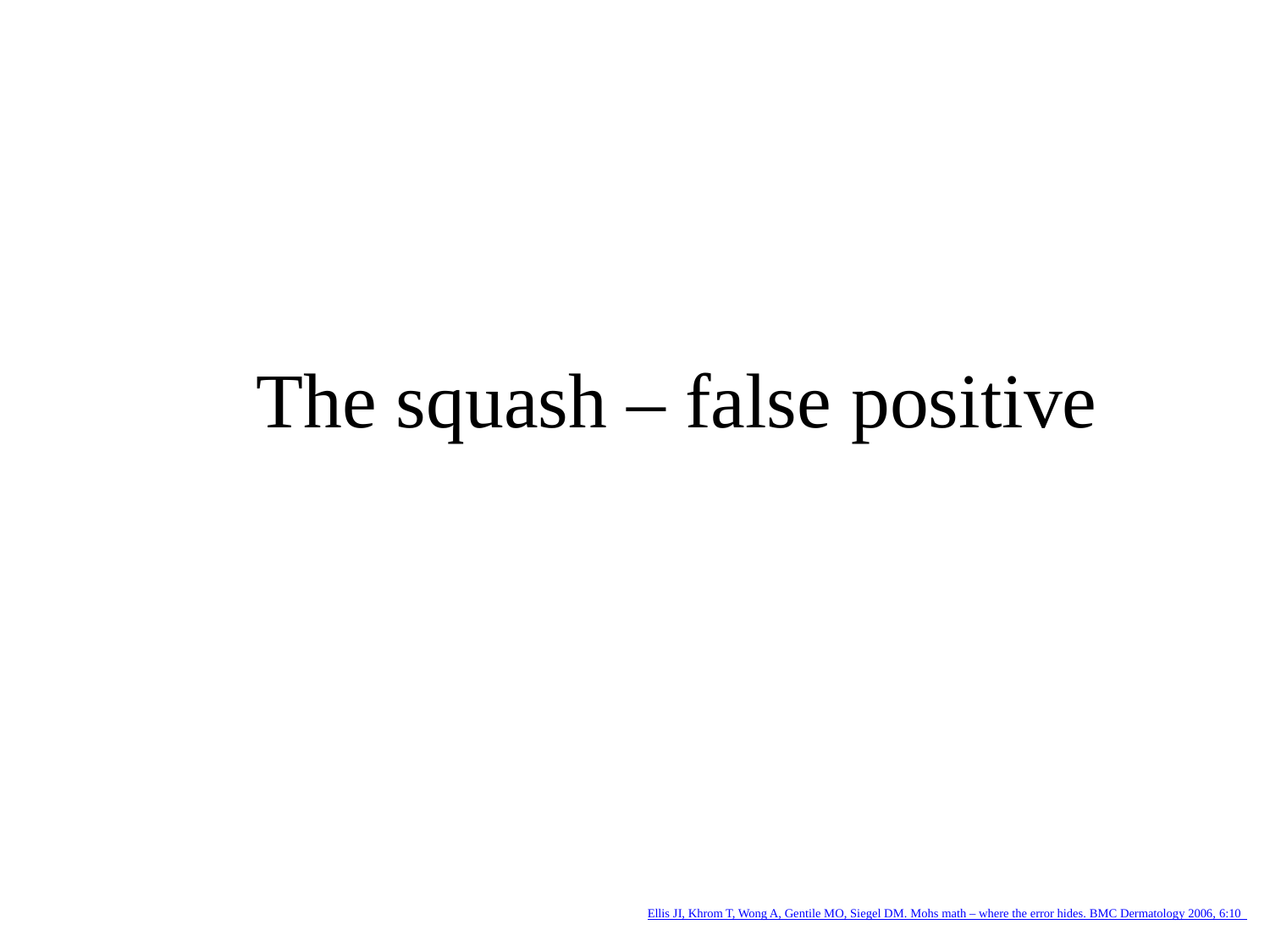

# The squash – false positive
Ellis JI, Khrom T, Wong A, Gentile MO, Siegel DM. Mohs math – where the error hides. BMC Dermatology 2006, 6:10

## Slide 3
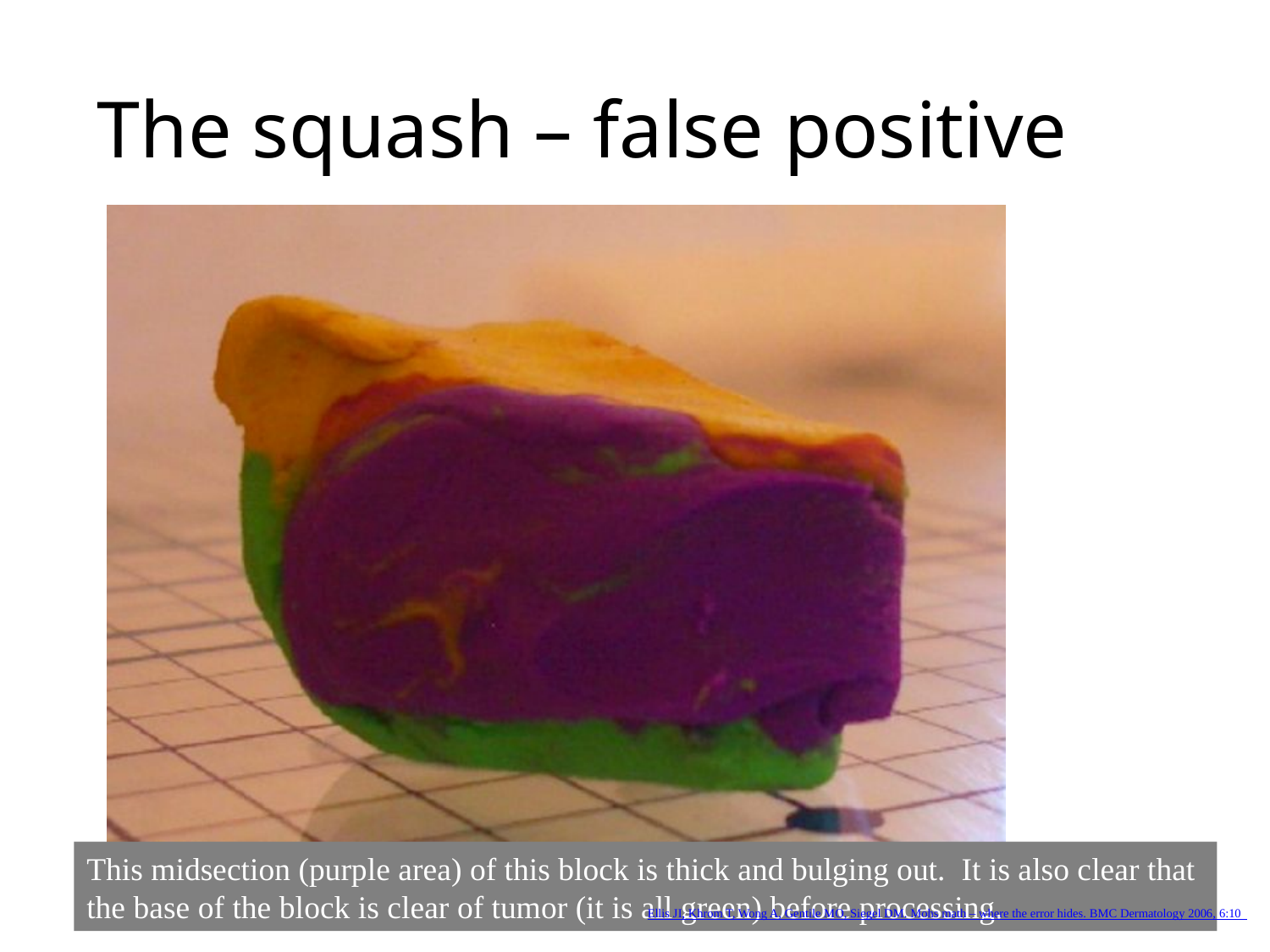

The squash – false positive
This midsection (purple area) of this block is thick and bulging out. It is also clear that the base of the block is clear of tumor (it is all green) before processing.
Ellis JI, Khrom T, Wong A, Gentile MO, Siegel DM. Mohs math – where the error hides. BMC Dermatology 2006, 6:10

## Slide 4
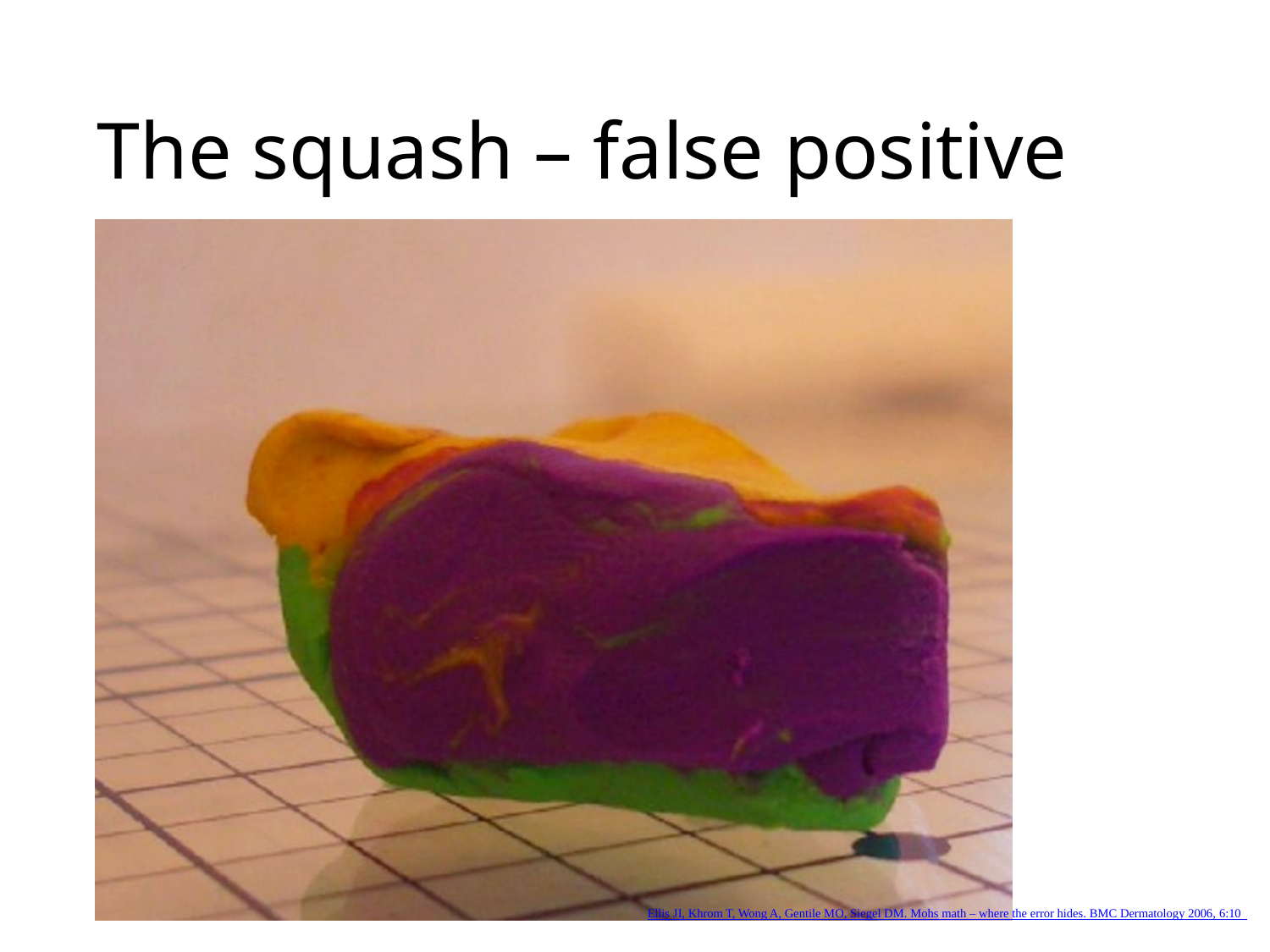

The squash – false positive
Ellis JI, Khrom T, Wong A, Gentile MO, Siegel DM. Mohs math – where the error hides. BMC Dermatology 2006, 6:10

## Slide 5
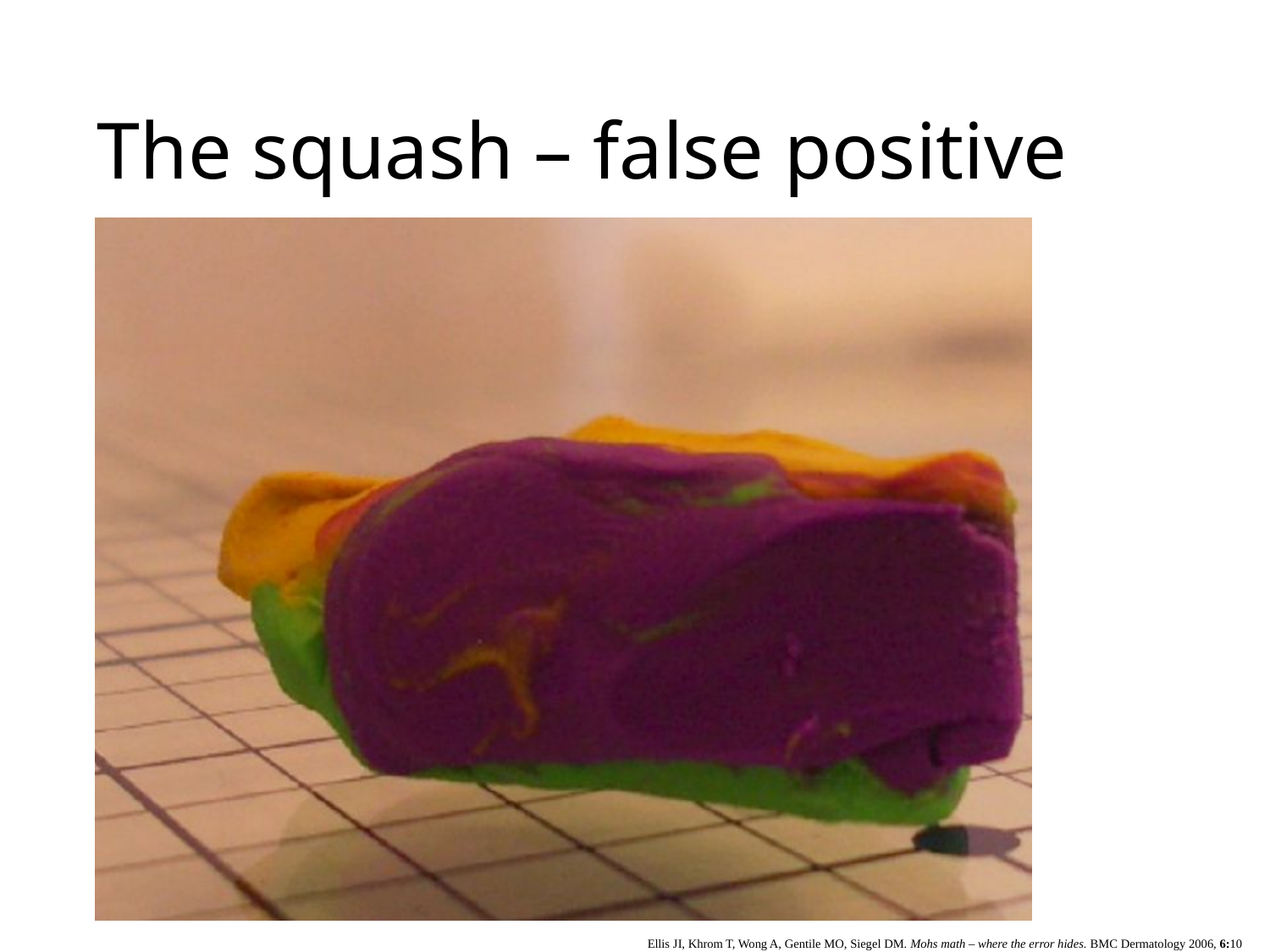

The squash – false positive
Ellis JI, Khrom T, Wong A, Gentile MO, Siegel DM. Mohs math – where the error hides. BMC Dermatology 2006, 6:10

## Slide 6
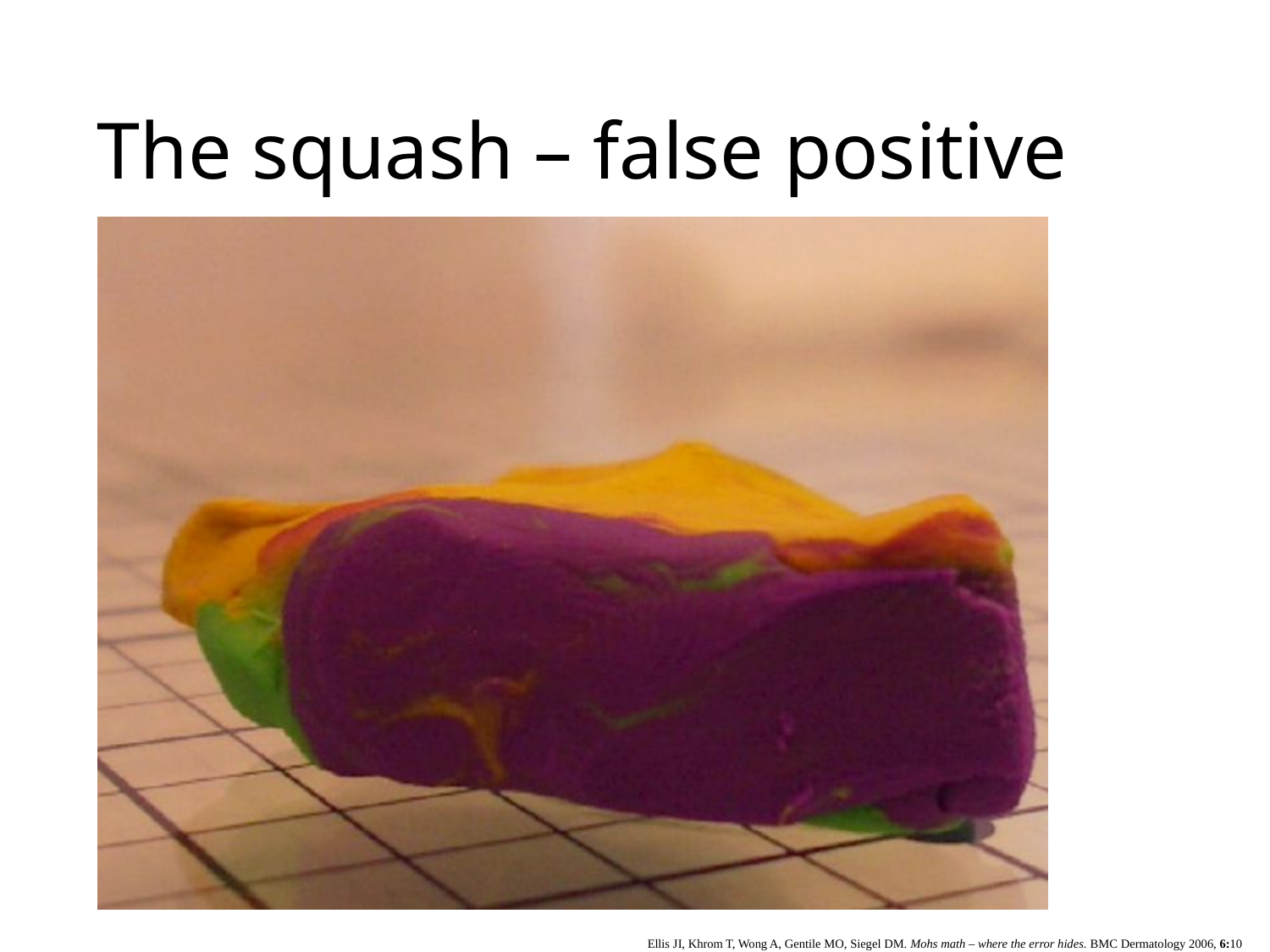

The squash – false positive
Ellis JI, Khrom T, Wong A, Gentile MO, Siegel DM. Mohs math – where the error hides. BMC Dermatology 2006, 6:10

## Slide 7
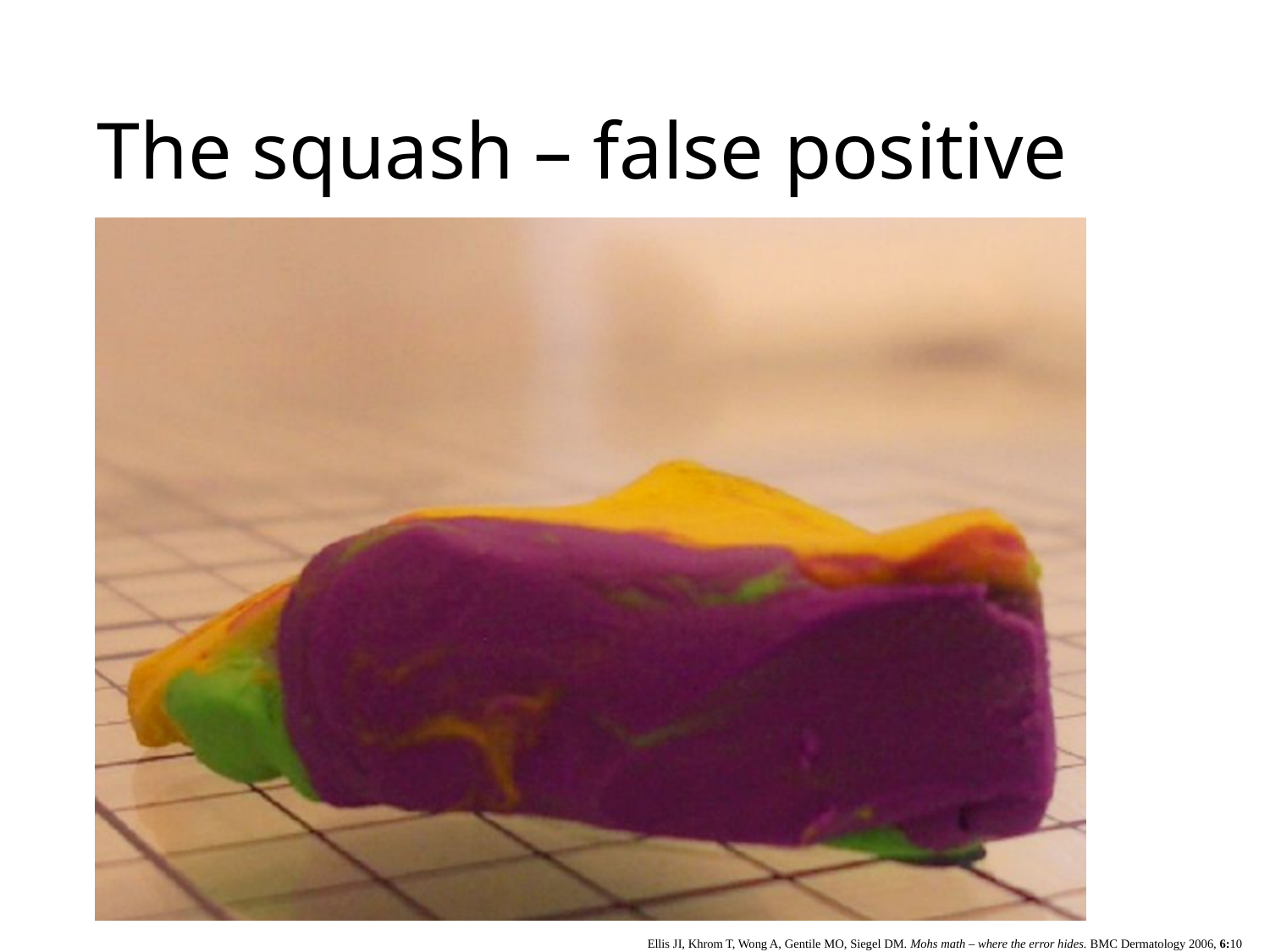

The squash – false positive
Ellis JI, Khrom T, Wong A, Gentile MO, Siegel DM. Mohs math – where the error hides. BMC Dermatology 2006, 6:10

## Slide 8
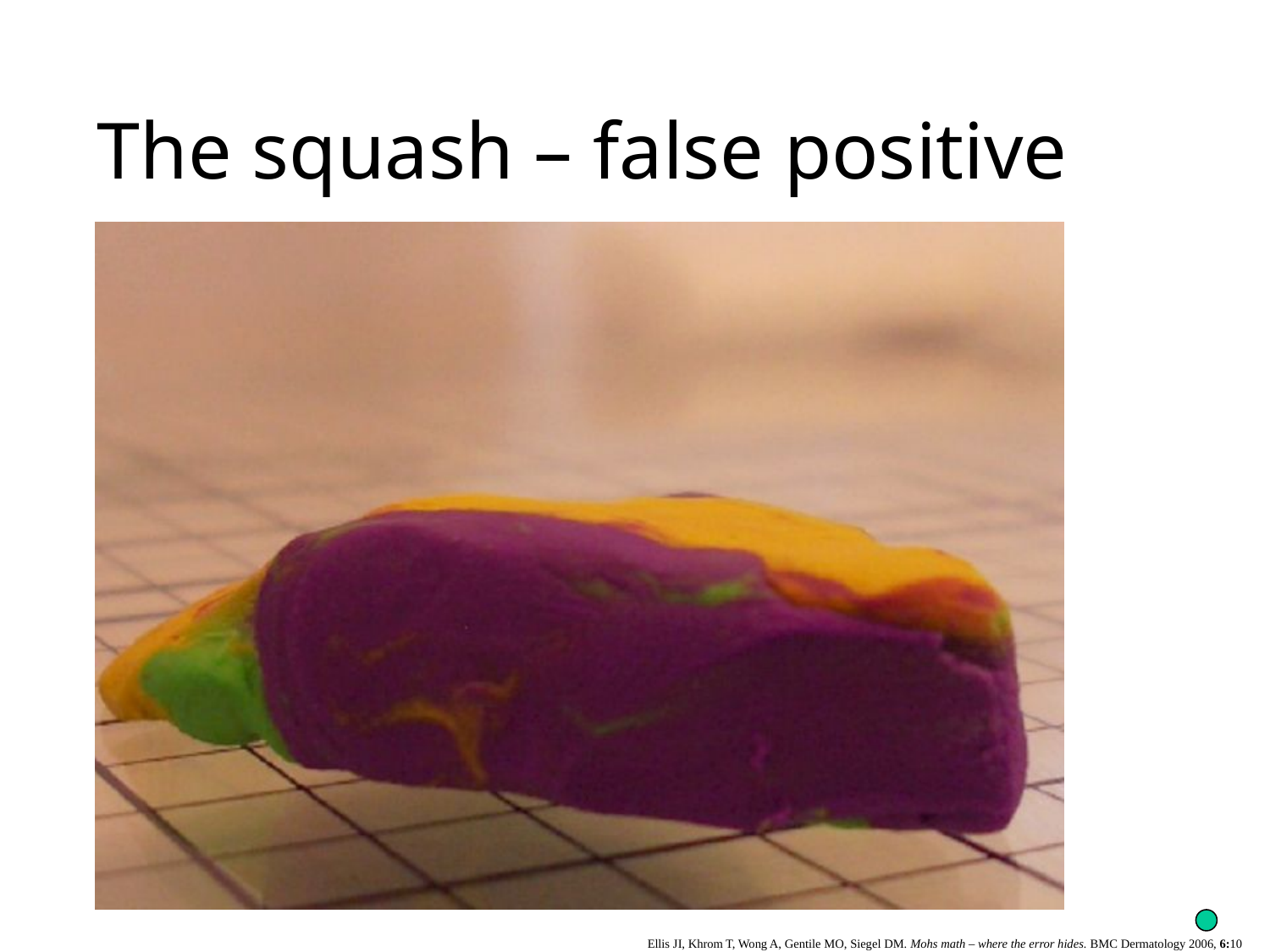

The squash – false positive
Ellis JI, Khrom T, Wong A, Gentile MO, Siegel DM. Mohs math – where the error hides. BMC Dermatology 2006, 6:10

## Slide 9
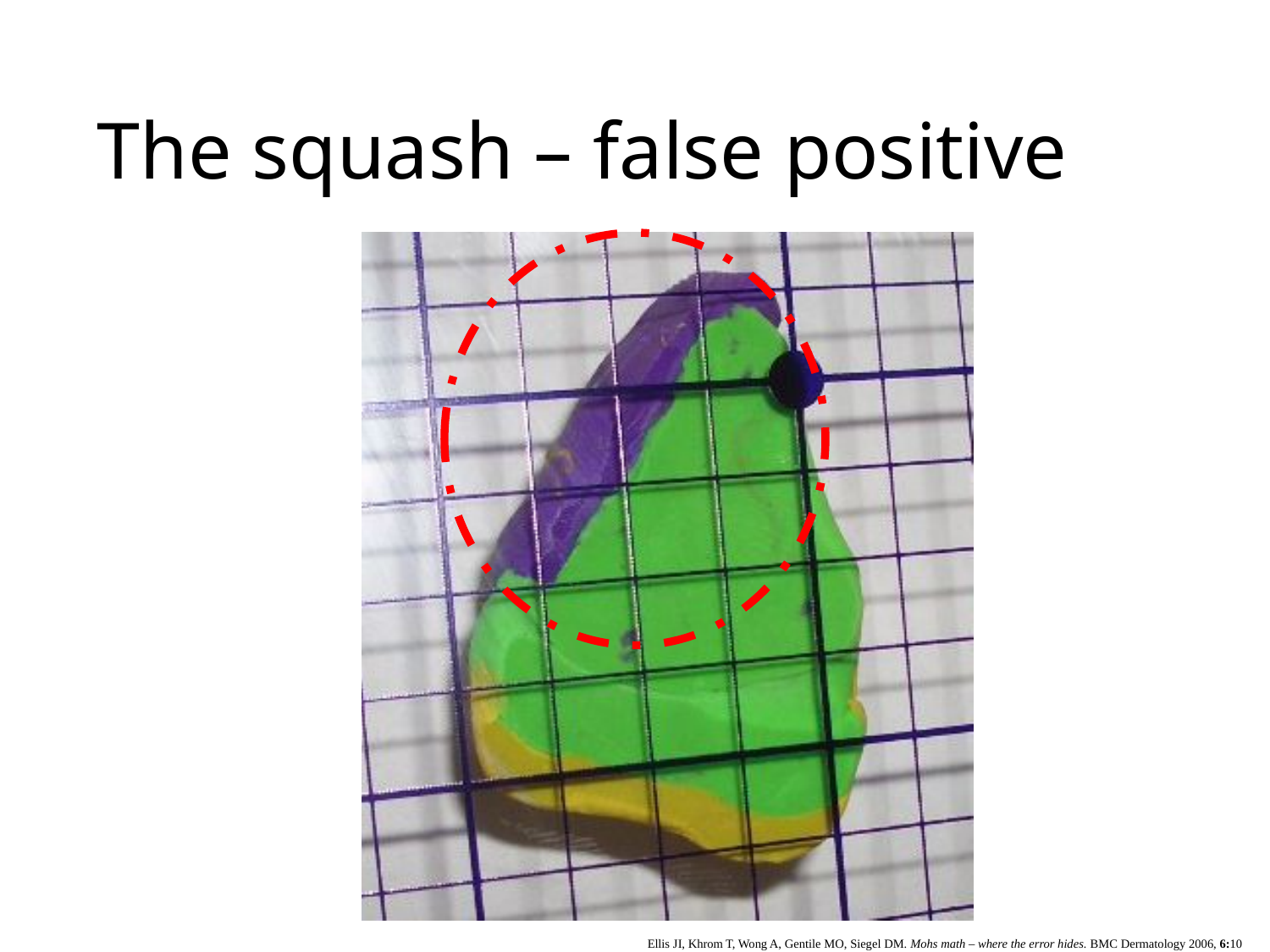

The squash – false positive
Ellis JI, Khrom T, Wong A, Gentile MO, Siegel DM. Mohs math – where the error hides. BMC Dermatology 2006, 6:10
