# Supplementary material for: Mohs math – where the error hides
Source: BMC Dermatol. 2006 Dec 6;6:10. doi: 10.1186/1471-5945-6-10 (PMC1769395; doi:10.1186/1471-5945-6-10)
Supplement: Additional File 4 — Tip lift error. Power point animation of a tip lift error [file 1471-5945-6-10-S4.ppt]

## Slide 1
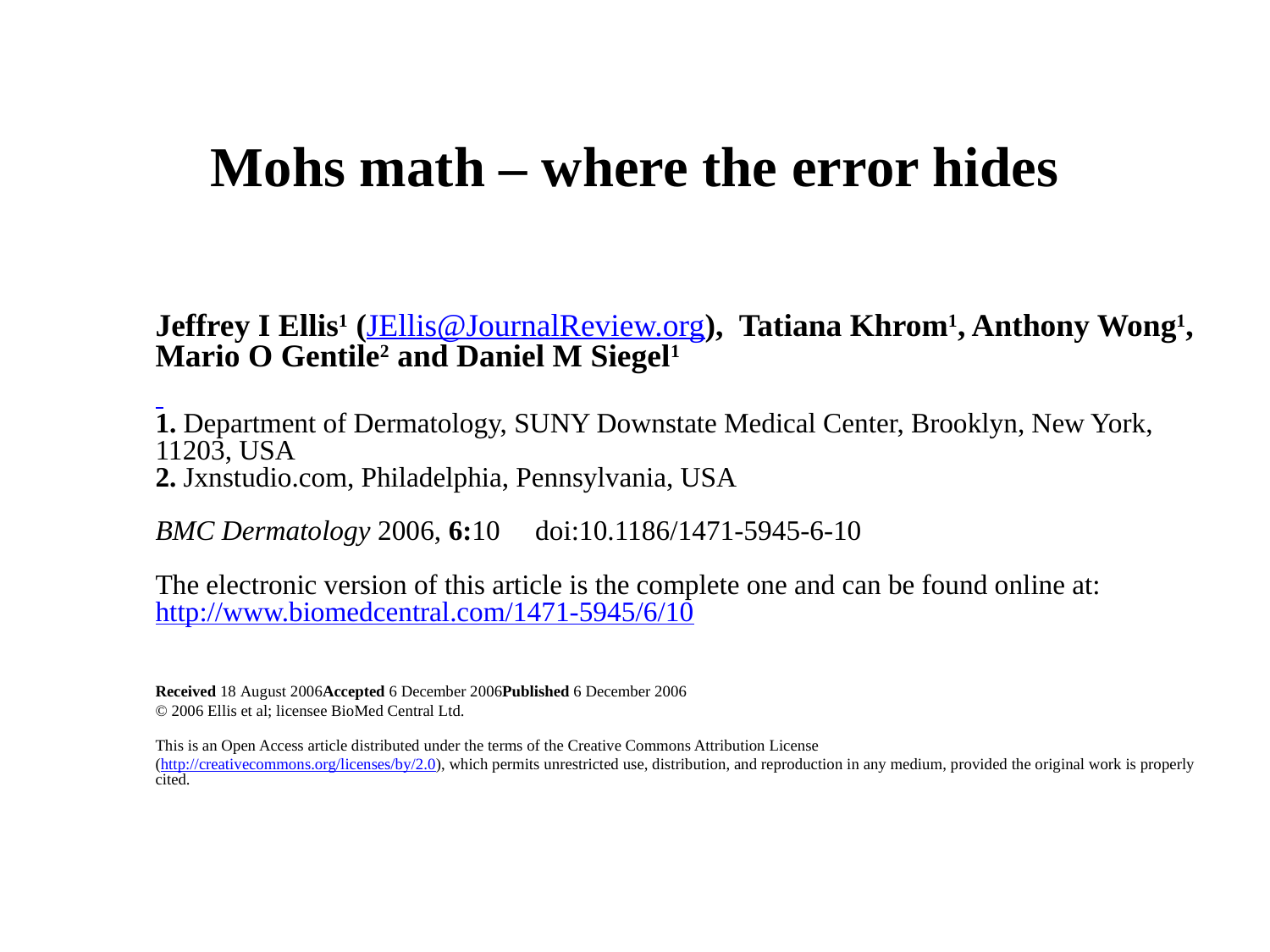

# Mohs math – where the error hides
Jeffrey I Ellis1 (JEllis@JournalReview.org), Tatiana Khrom1, Anthony Wong1, Mario O Gentile2 and Daniel M Siegel1
1. Department of Dermatology, SUNY Downstate Medical Center, Brooklyn, New York, 11203, USA
2. Jxnstudio.com, Philadelphia, Pennsylvania, USA
BMC Dermatology 2006, 6:10     doi:10.1186/1471-5945-6-10
The electronic version of this article is the complete one and can be found online at: http://www.biomedcentral.com/1471-5945/6/10
Received 18 August 2006Accepted 6 December 2006Published 6 December 2006
© 2006 Ellis et al; licensee BioMed Central Ltd.
This is an Open Access article distributed under the terms of the Creative Commons Attribution License
(http://creativecommons.org/licenses/by/2.0), which permits unrestricted use, distribution, and reproduction in any medium, provided the original work is properly cited.

## Slide 2
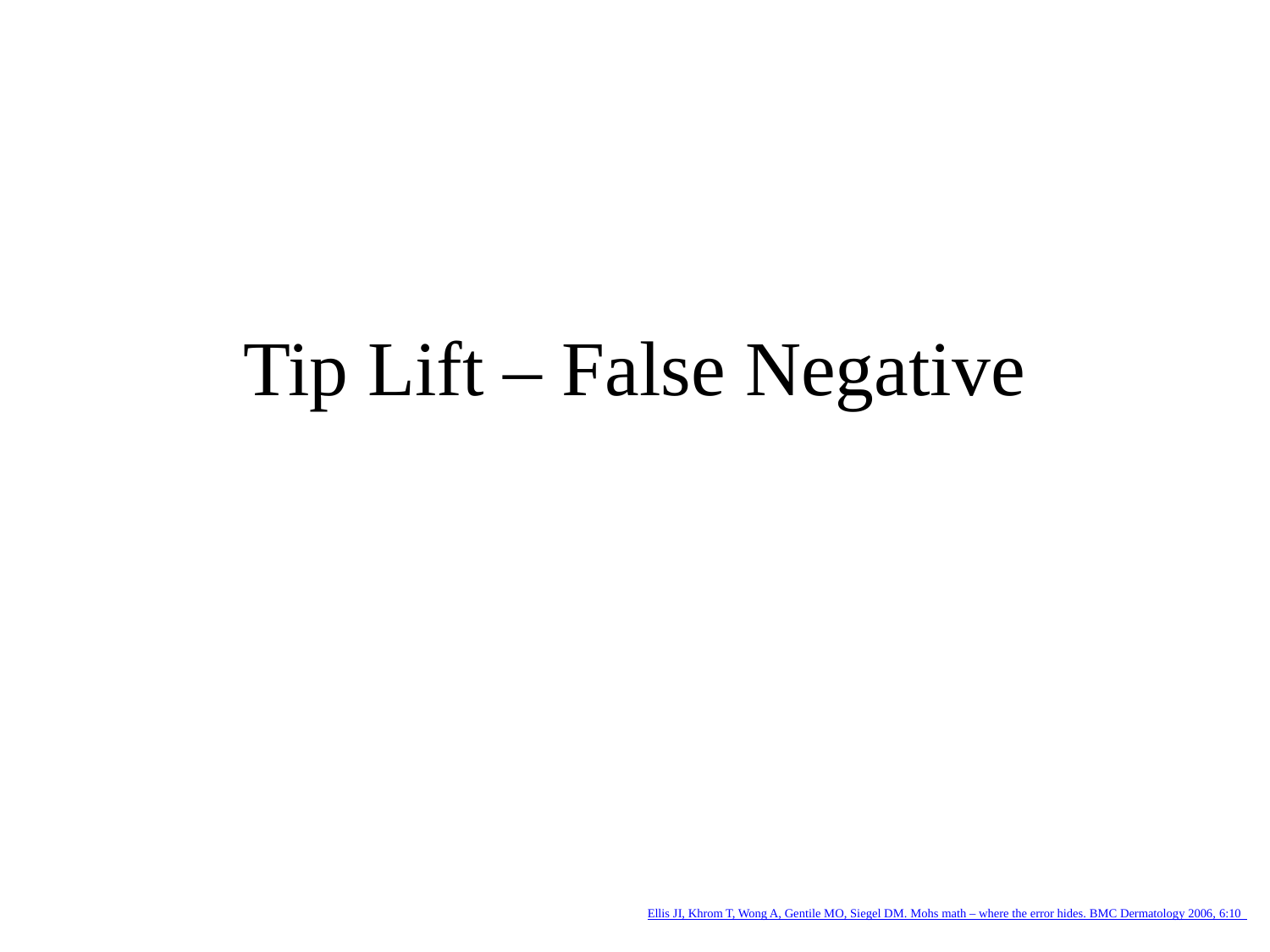

# Tip Lift – False Negative
Ellis JI, Khrom T, Wong A, Gentile MO, Siegel DM. Mohs math – where the error hides. BMC Dermatology 2006, 6:10

## Slide 3
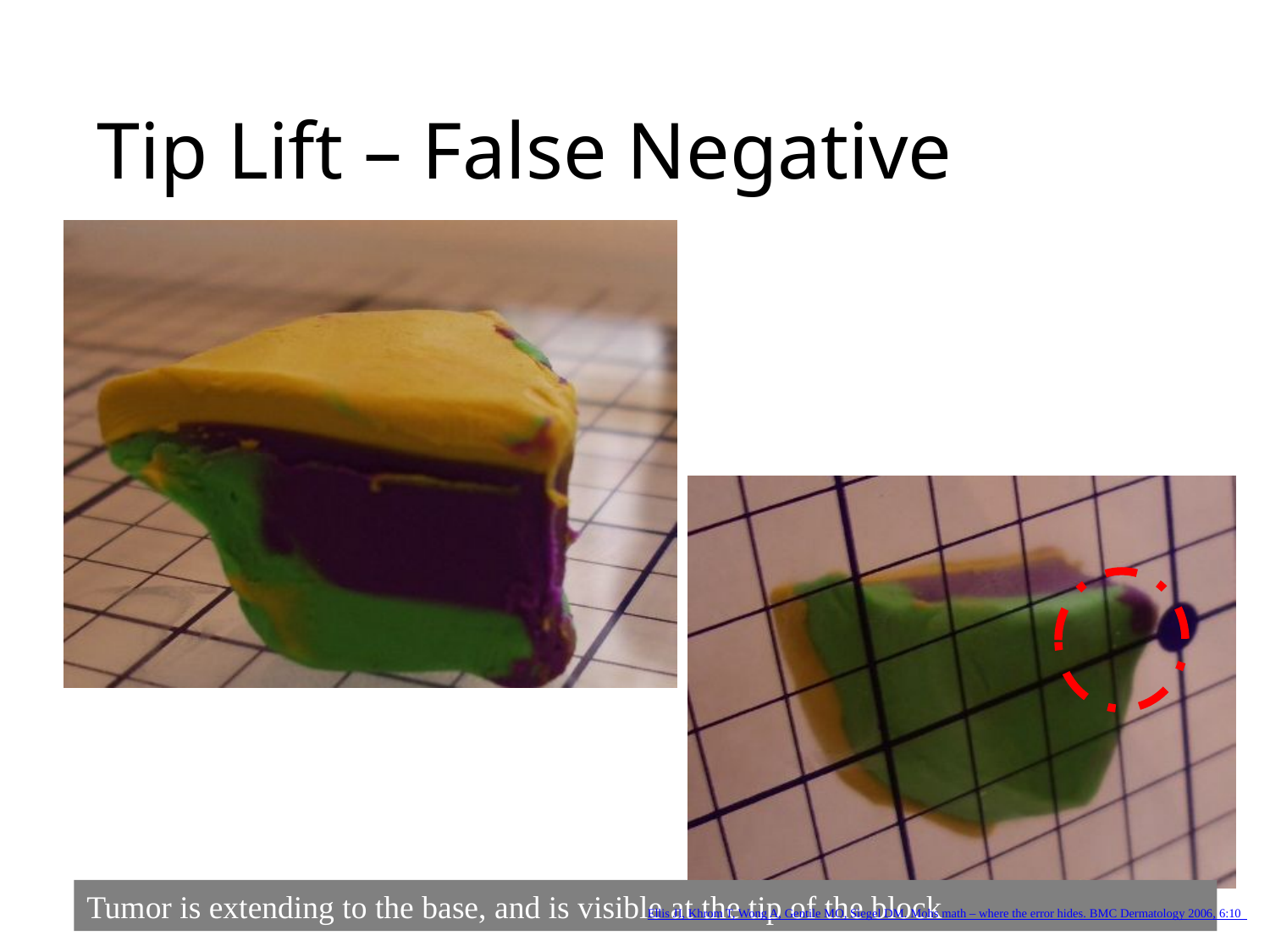

Tip Lift – False Negative
Tumor is extending to the base, and is visible at the tip of the block
Ellis JI, Khrom T, Wong A, Gentile MO, Siegel DM. Mohs math – where the error hides. BMC Dermatology 2006, 6:10

## Slide 4
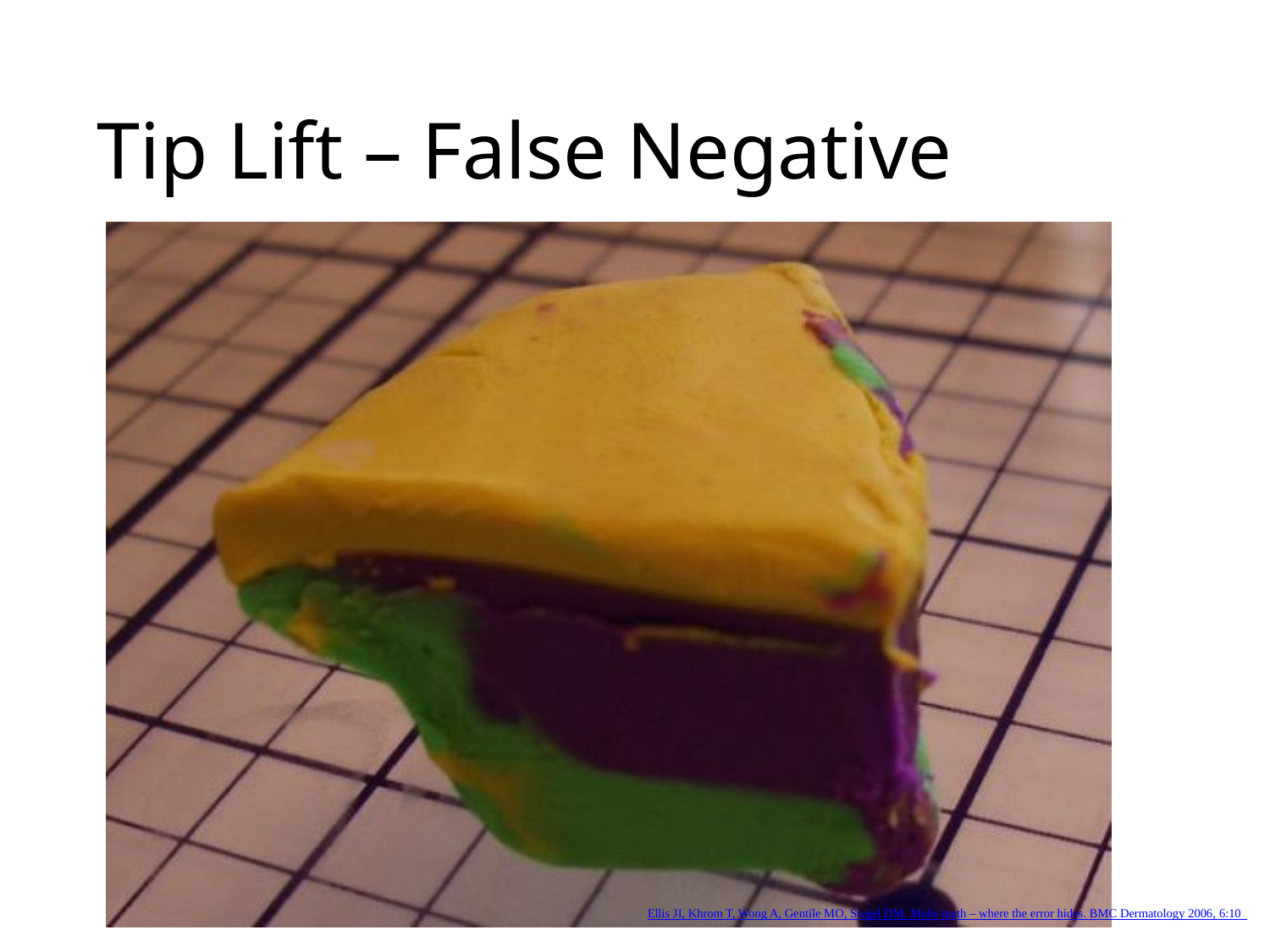

Tip Lift – False Negative
Ellis JI, Khrom T, Wong A, Gentile MO, Siegel DM. Mohs math – where the error hides. BMC Dermatology 2006, 6:10

## Slide 5
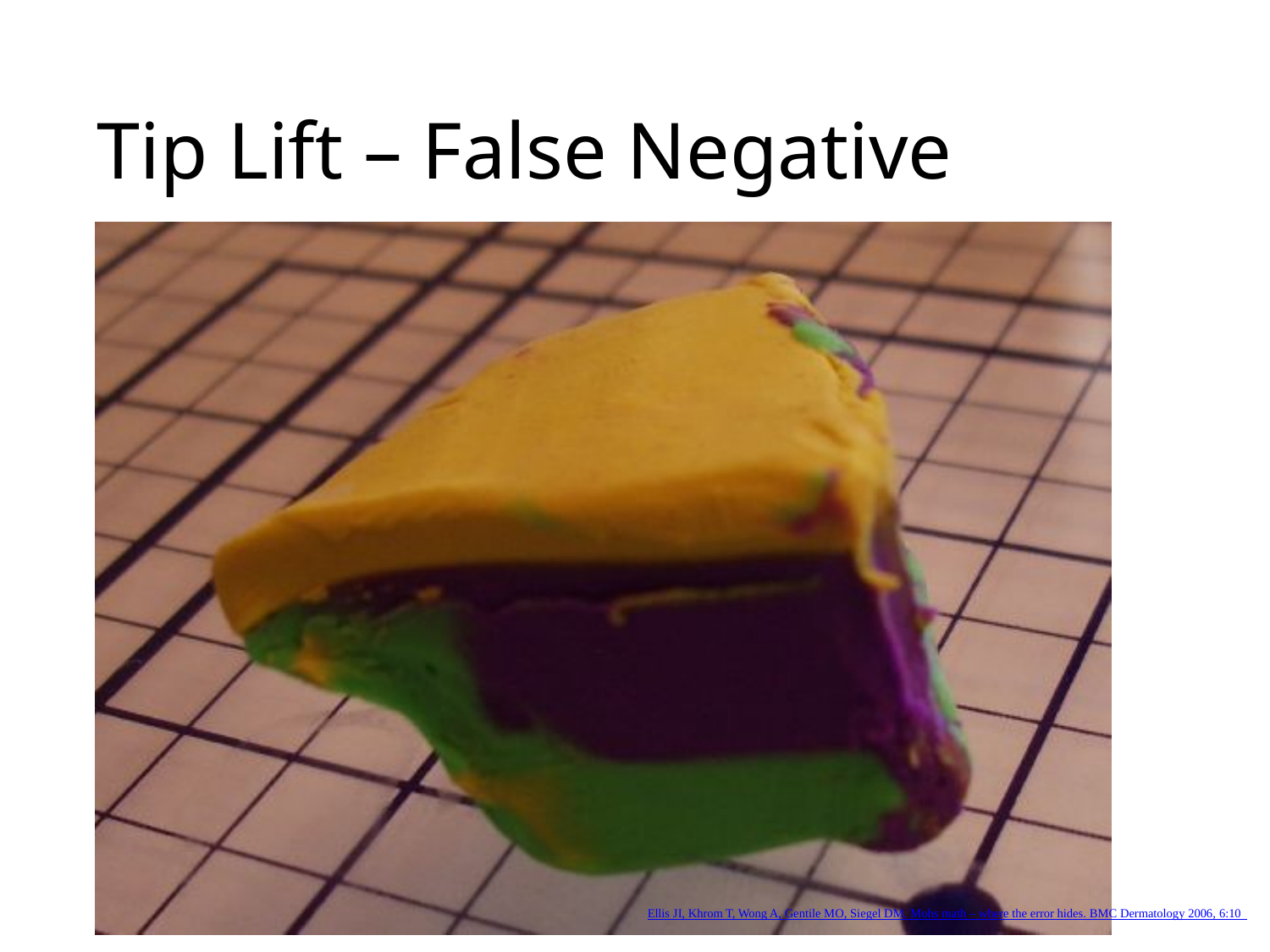

Tip Lift – False Negative
Ellis JI, Khrom T, Wong A, Gentile MO, Siegel DM. Mohs math – where the error hides. BMC Dermatology 2006, 6:10

## Slide 6
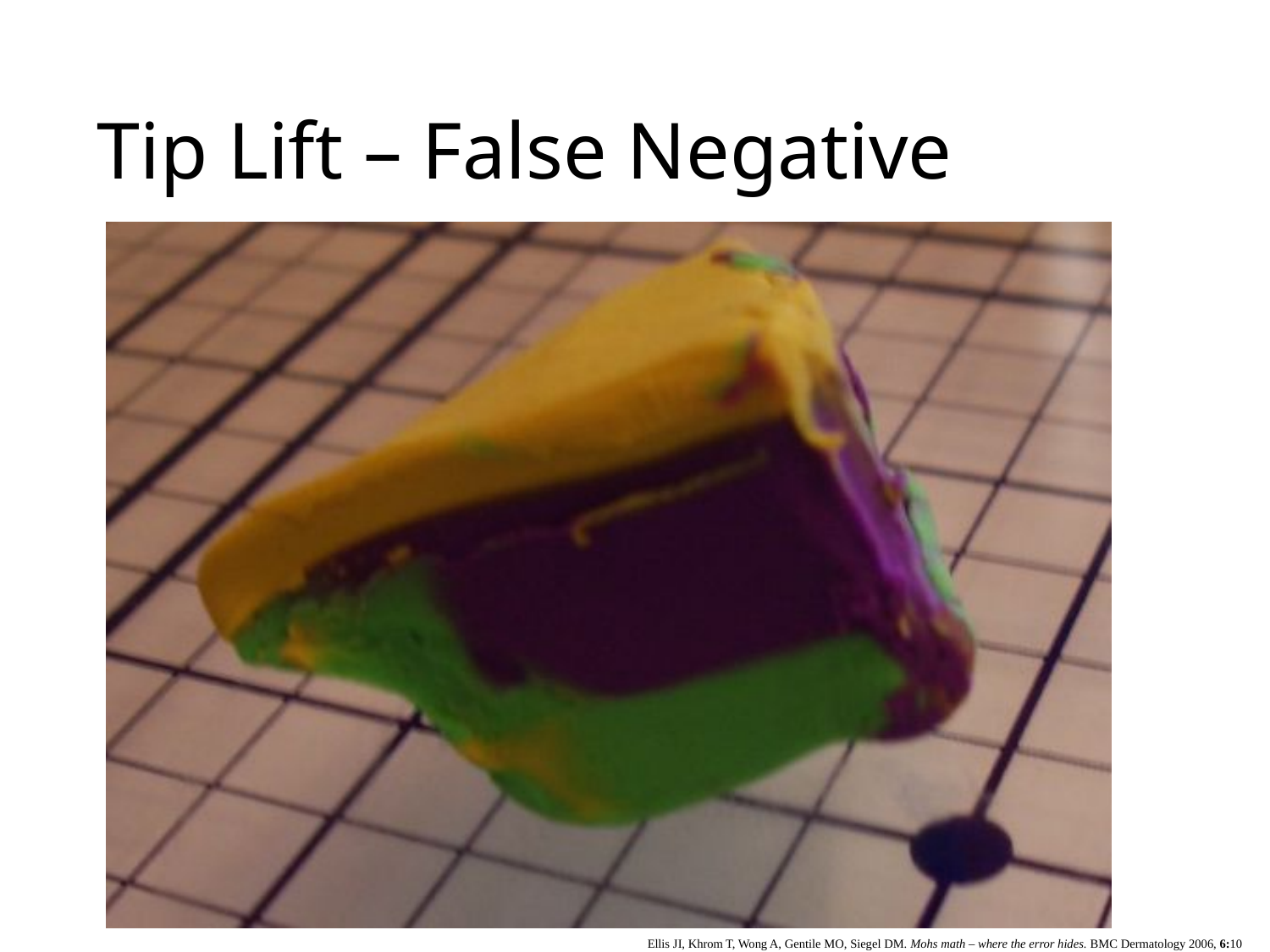

Tip Lift – False Negative
Ellis JI, Khrom T, Wong A, Gentile MO, Siegel DM. Mohs math – where the error hides. BMC Dermatology 2006, 6:10

## Slide 7
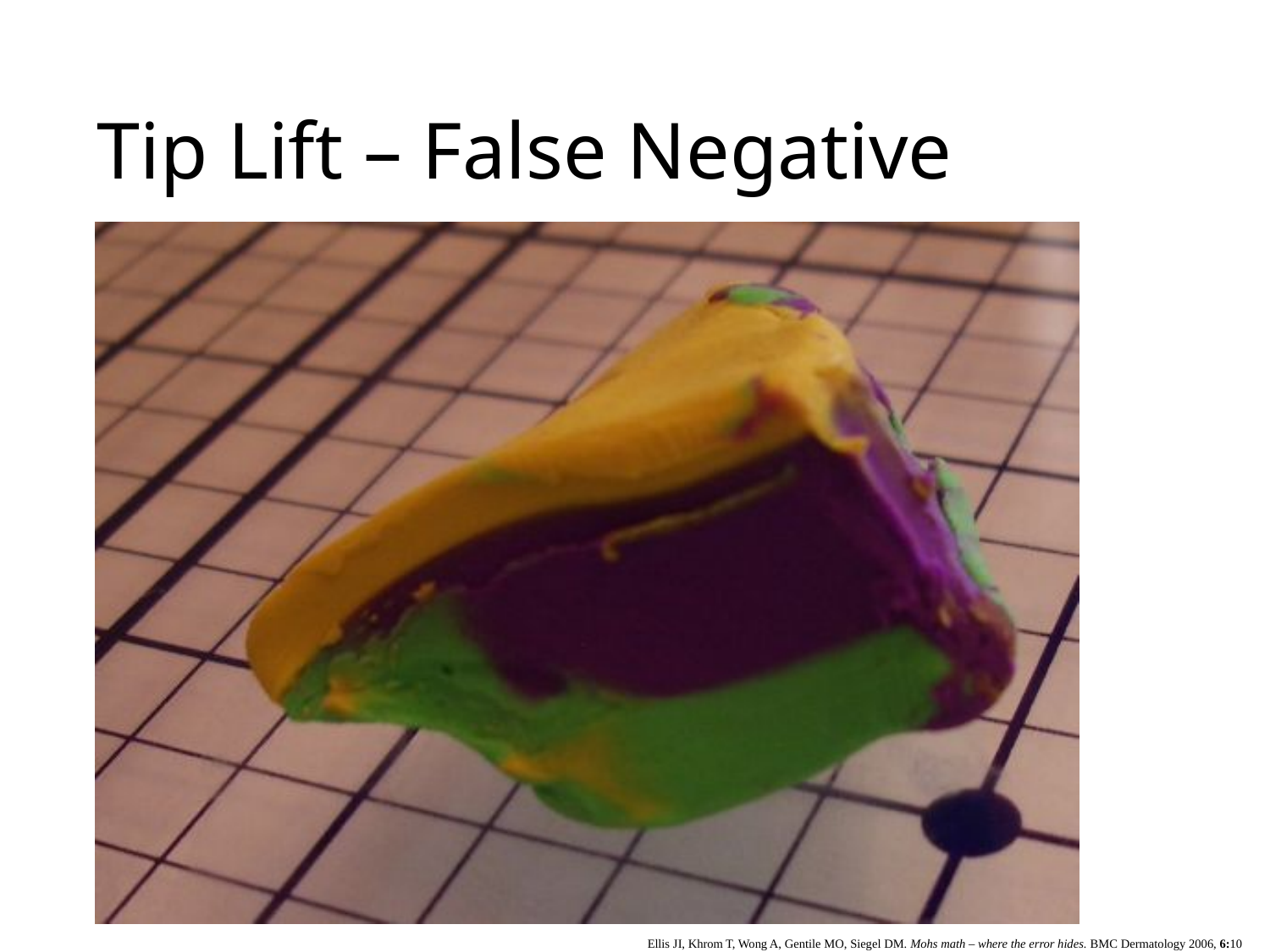

Tip Lift – False Negative
Ellis JI, Khrom T, Wong A, Gentile MO, Siegel DM. Mohs math – where the error hides. BMC Dermatology 2006, 6:10

## Slide 8
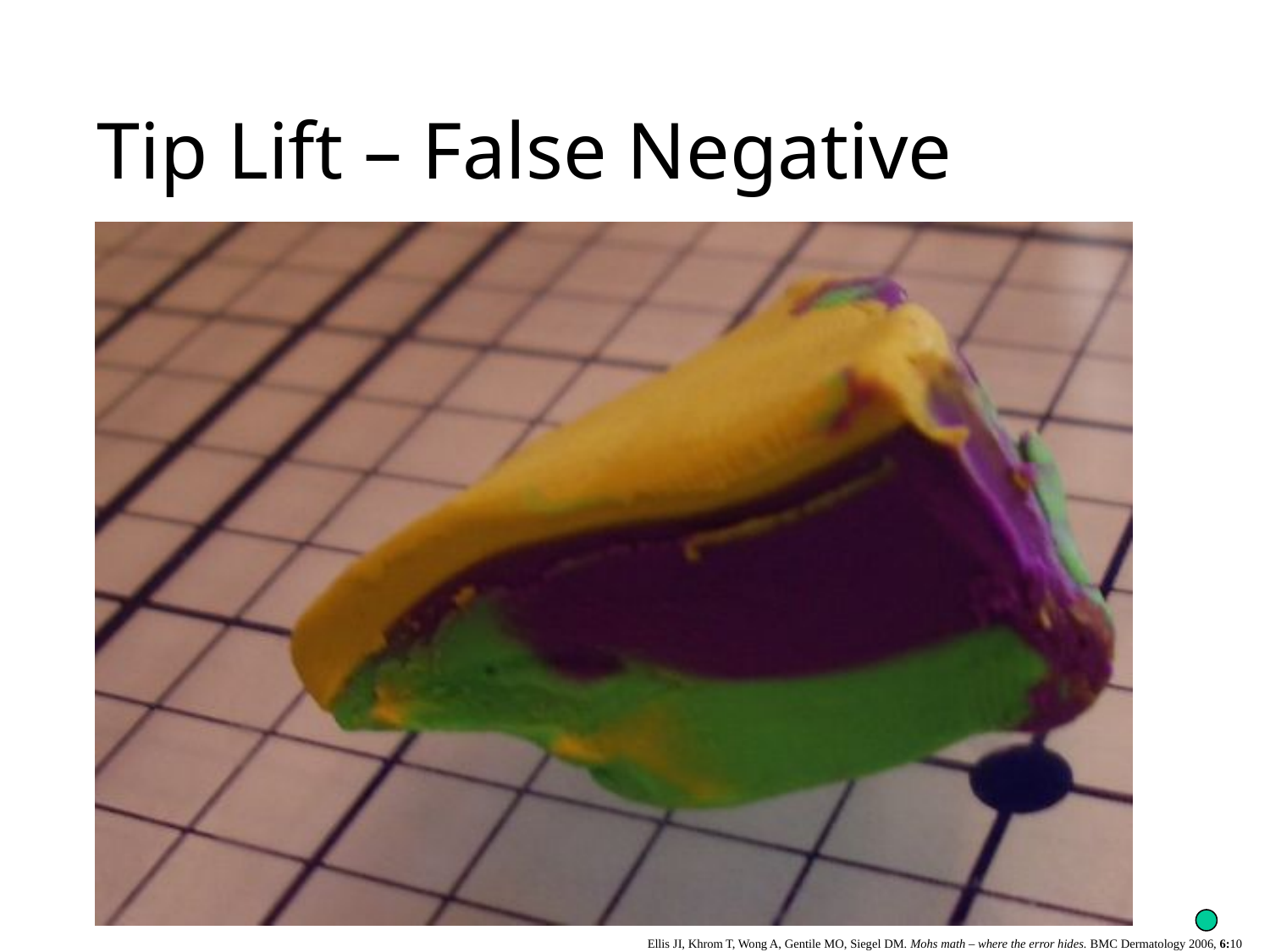

Tip Lift – False Negative
Ellis JI, Khrom T, Wong A, Gentile MO, Siegel DM. Mohs math – where the error hides. BMC Dermatology 2006, 6:10

## Slide 9
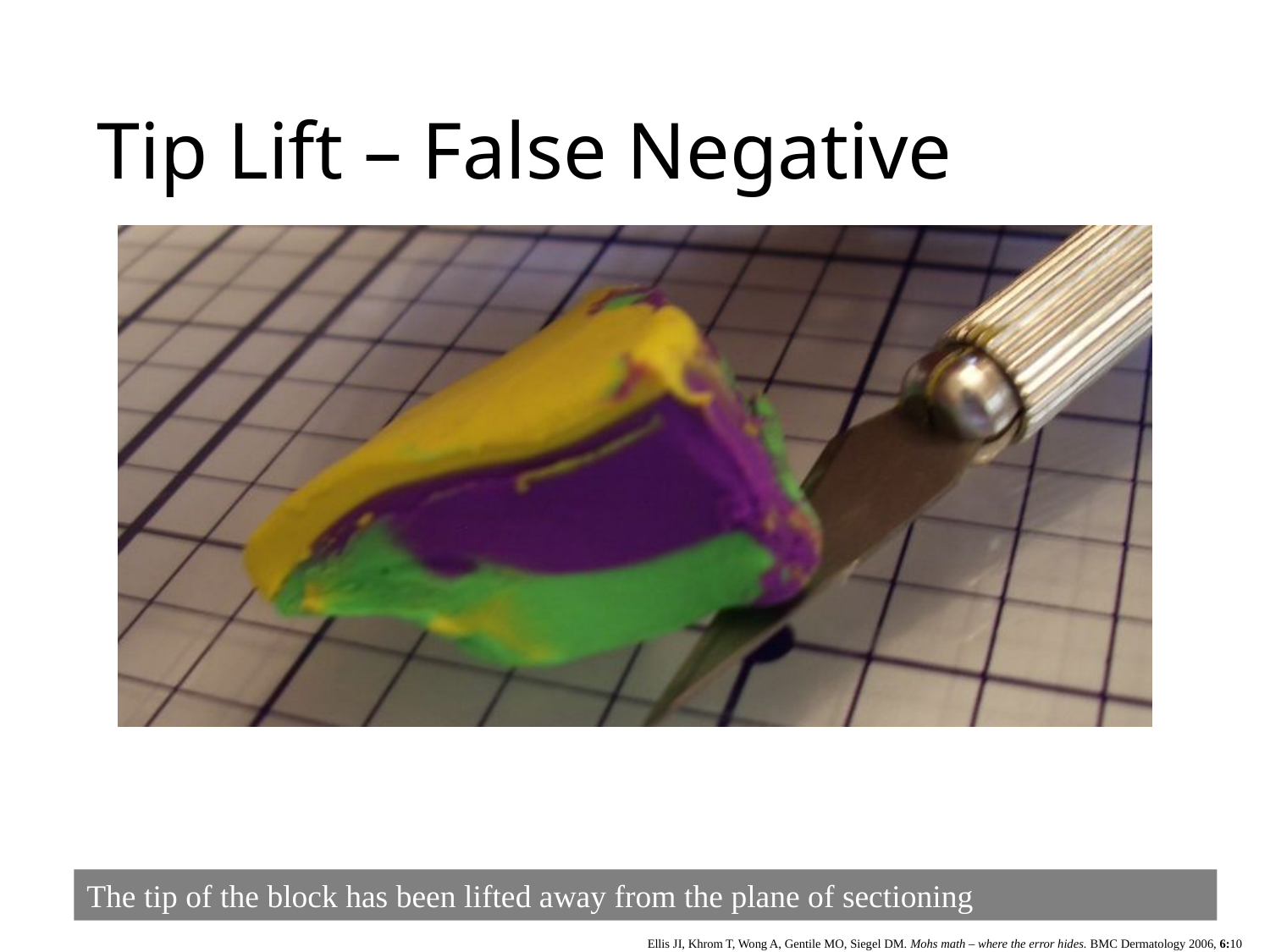

Tip Lift – False Negative
The tip of the block has been lifted away from the plane of sectioning
Ellis JI, Khrom T, Wong A, Gentile MO, Siegel DM. Mohs math – where the error hides. BMC Dermatology 2006, 6:10

## Slide 10
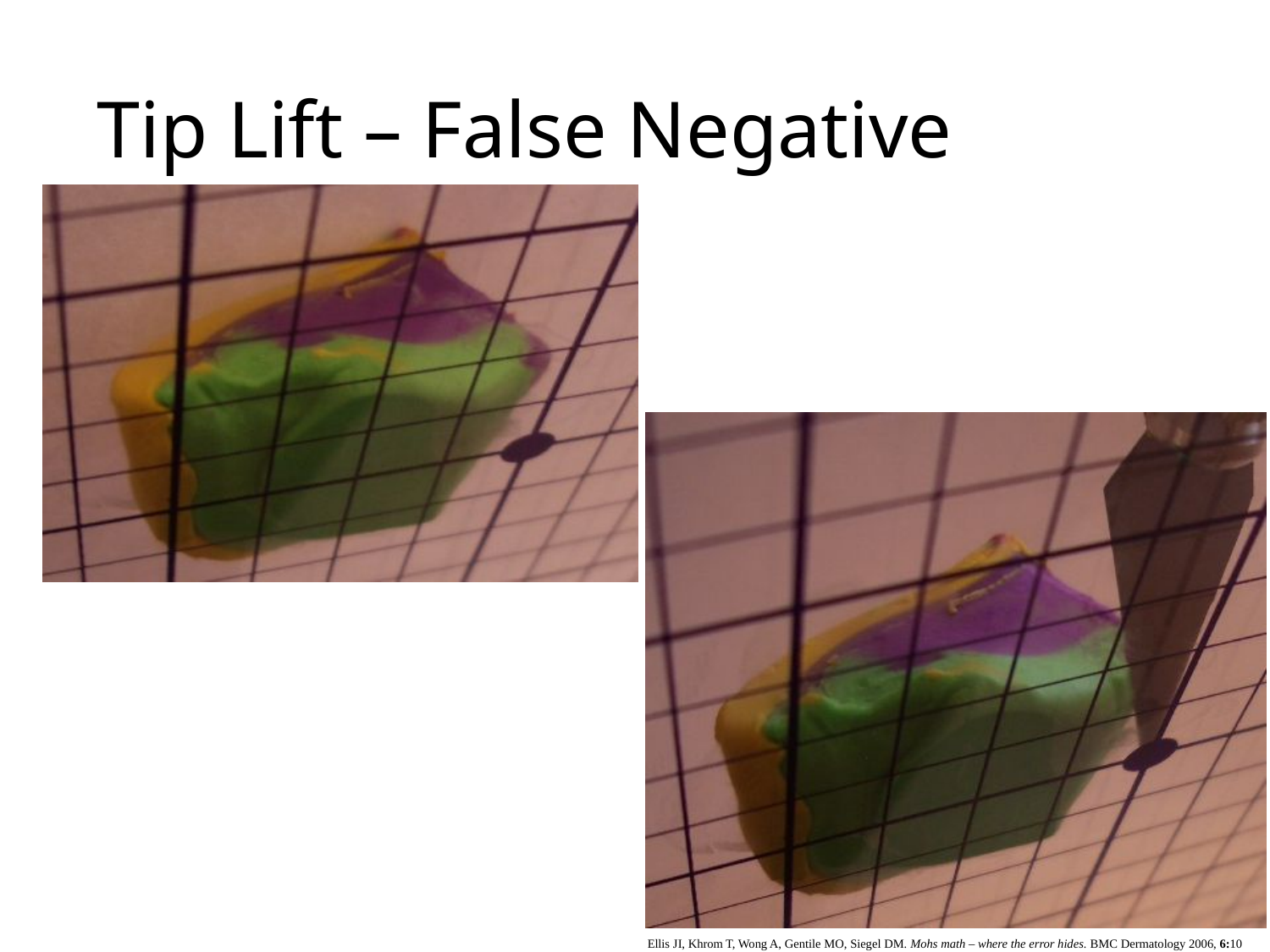

Tip Lift – False Negative
Ellis JI, Khrom T, Wong A, Gentile MO, Siegel DM. Mohs math – where the error hides. BMC Dermatology 2006, 6:10
