# Supplementary material for: Mohs math – where the error hides
Source: BMC Dermatol. 2006 Dec 6;6:10. doi: 10.1186/1471-5945-6-10 (PMC1769395; doi:10.1186/1471-5945-6-10)
Supplement: Additional File 5 — Thin section collapse error. Power point animation of a thin section collapse error [file 1471-5945-6-10-S5.ppt]

## Slide 1
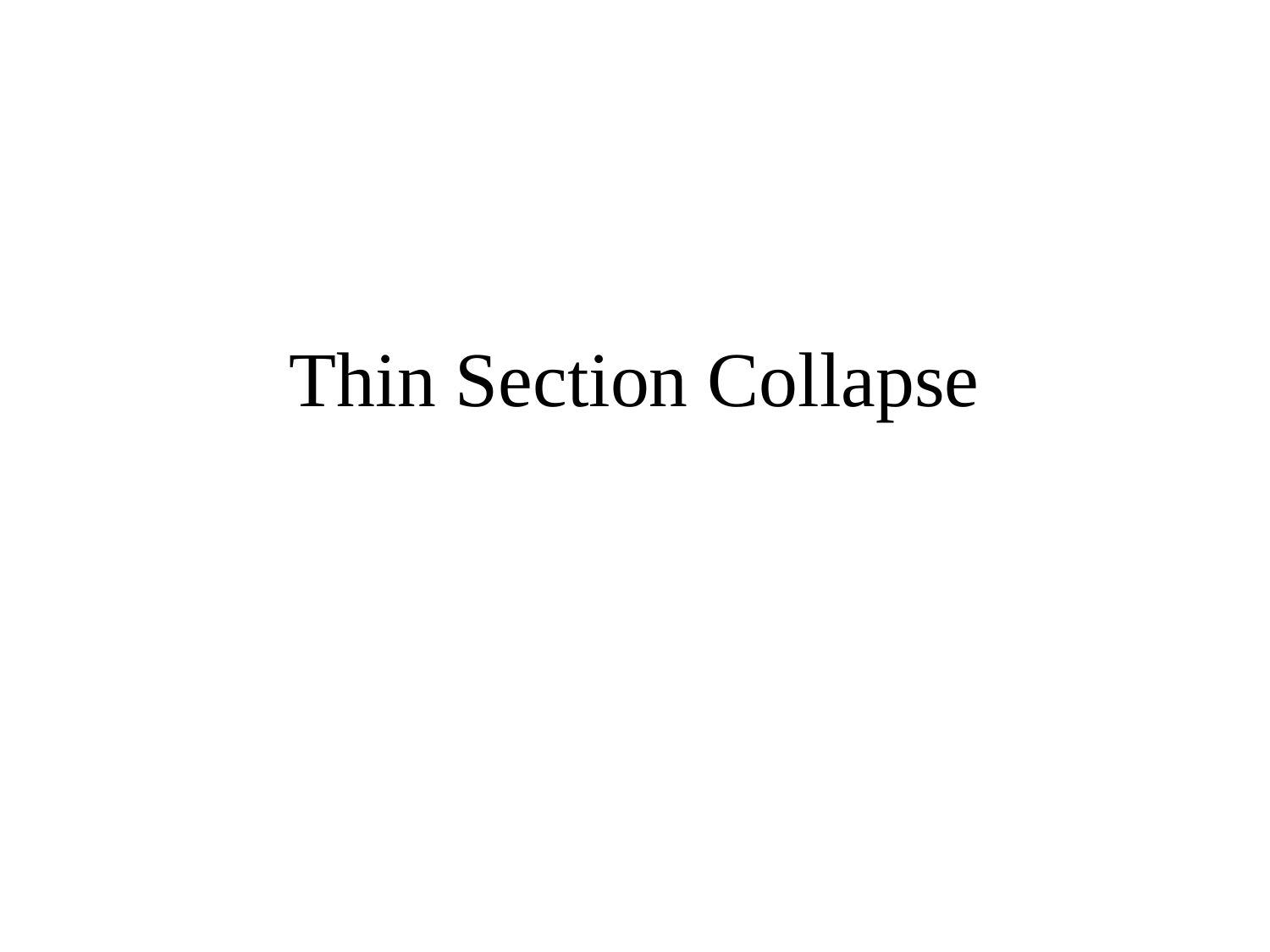

# Thin Section Collapse

## Slide 2
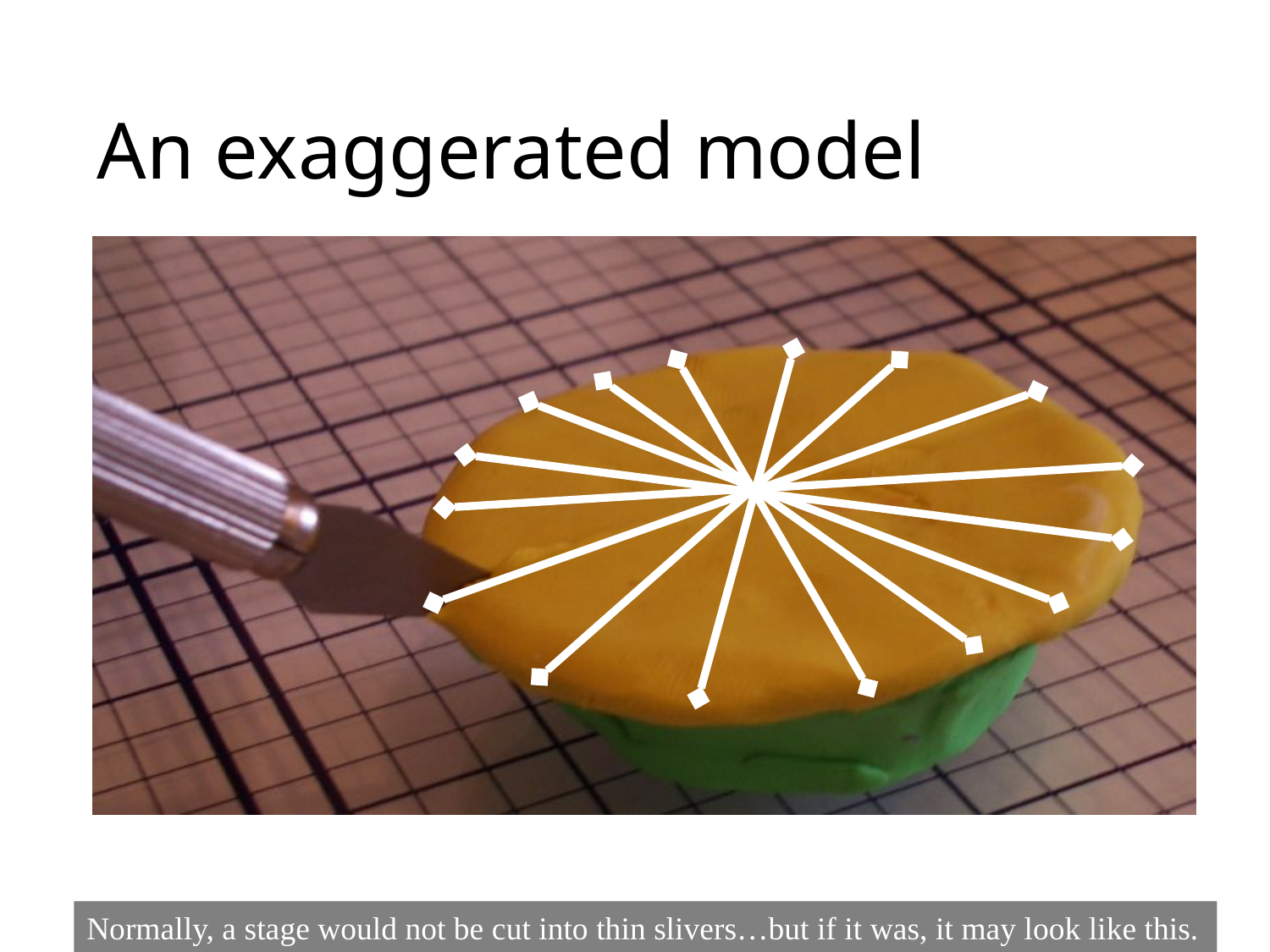

An exaggerated model
Normally, a stage would not be cut into thin slivers…but if it was, it may look like this.

## Slide 3
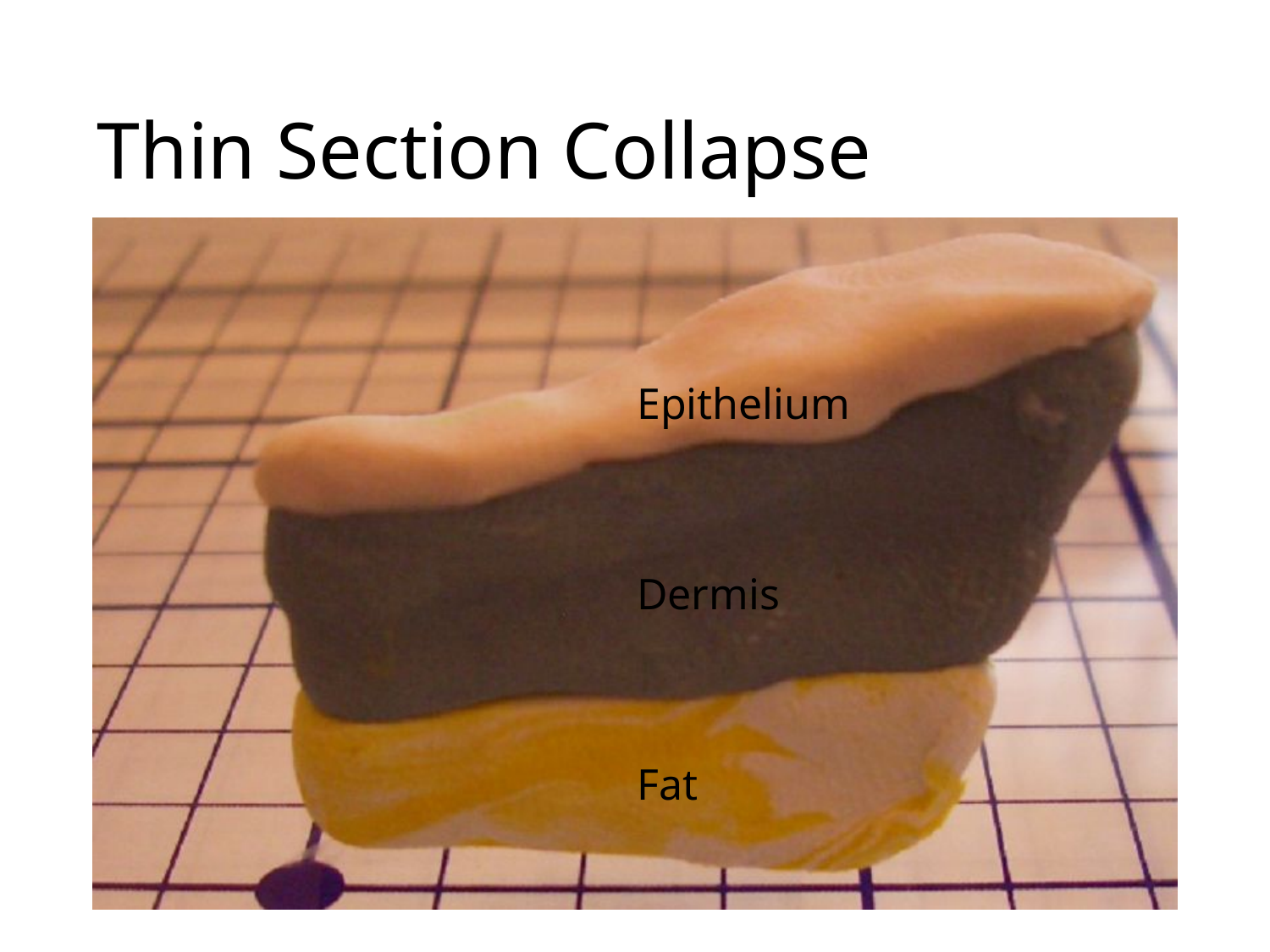

Thin Section Collapse
Epithelium
Dermis
Fat

## Slide 4
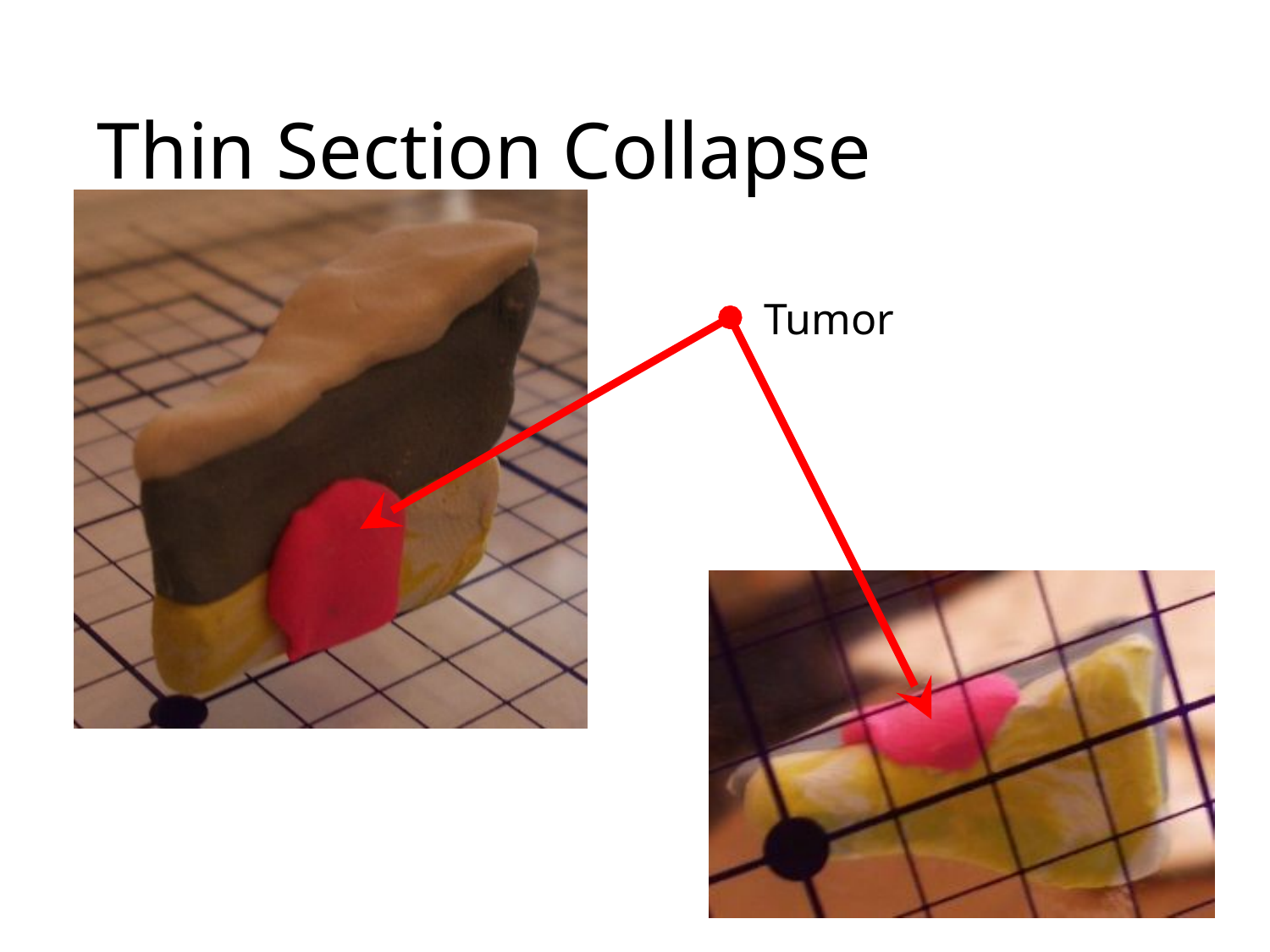

Thin Section Collapse
Tumor

## Slide 5
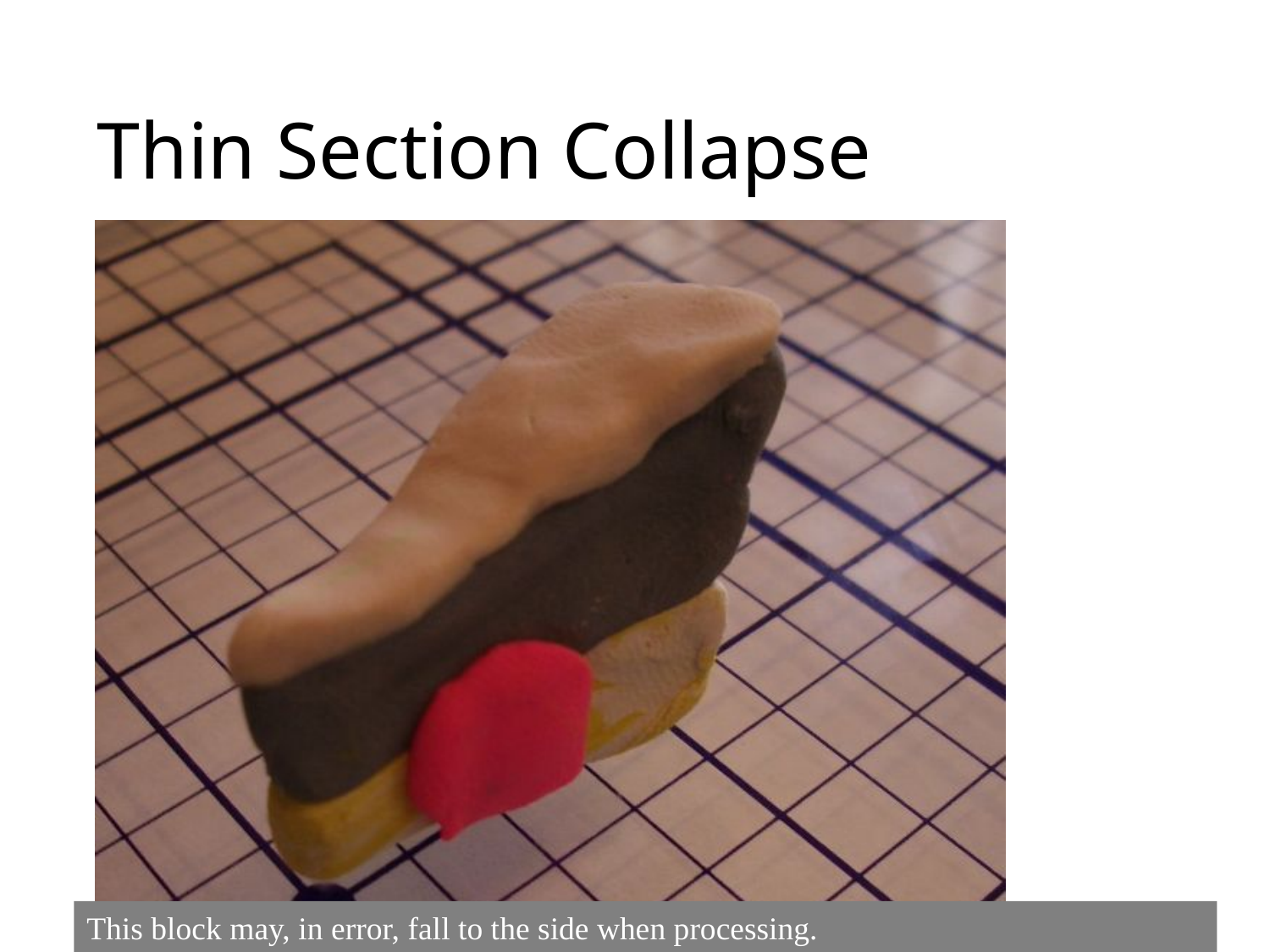

Thin Section Collapse
This block may, in error, fall to the side when processing.

## Slide 6
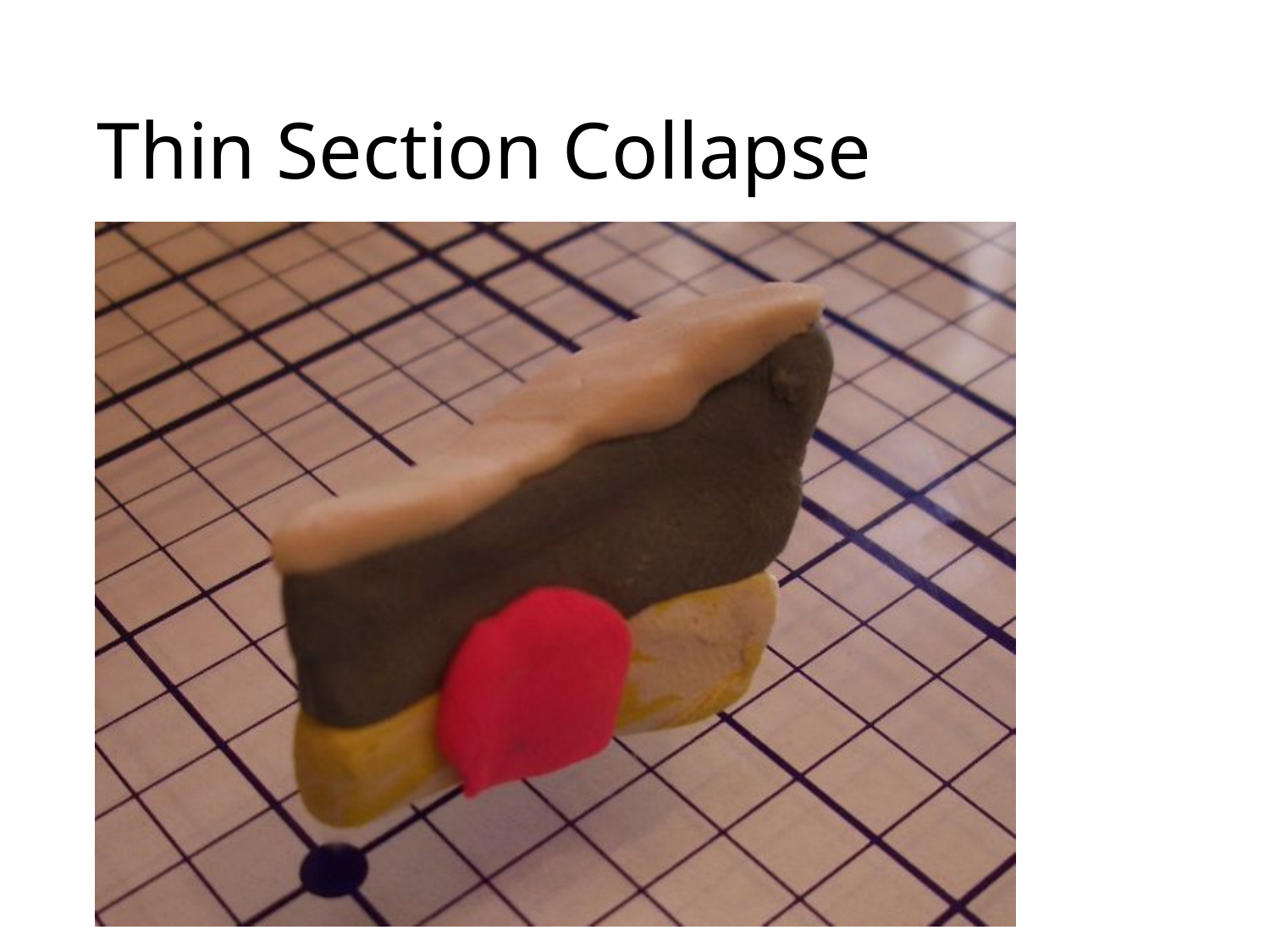

Thin Section Collapse

## Slide 7
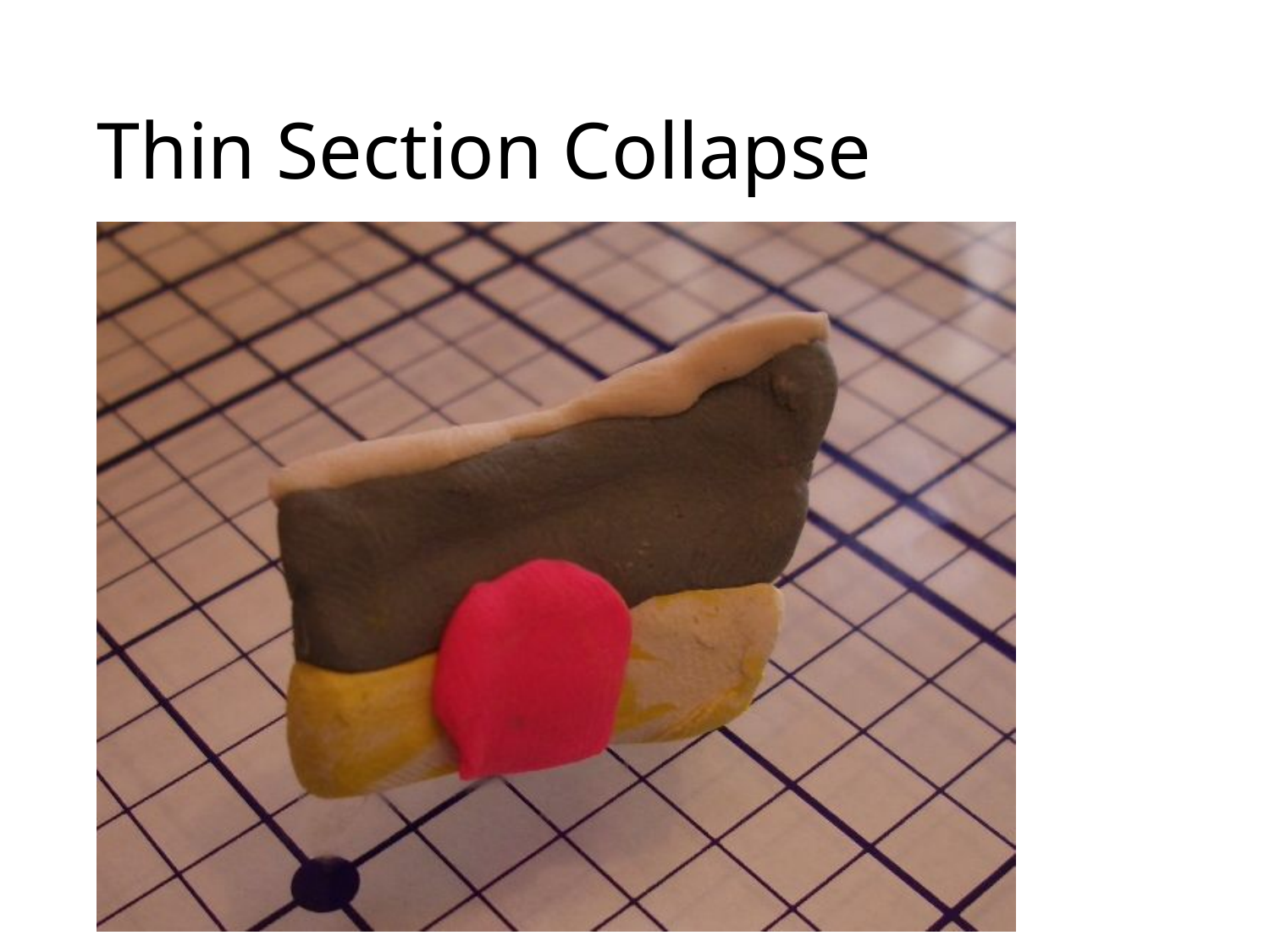

Thin Section Collapse

## Slide 8
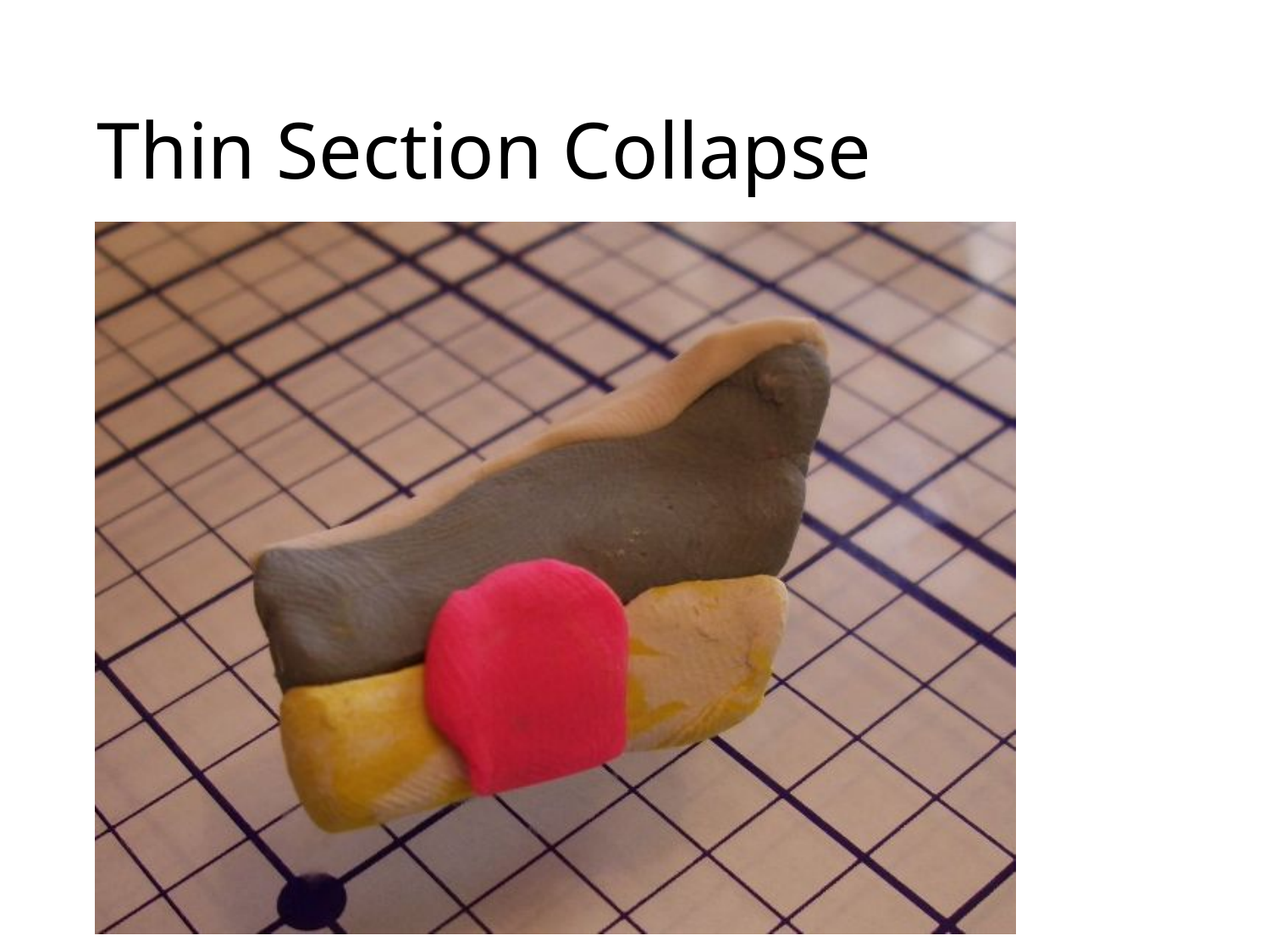

Thin Section Collapse

## Slide 9
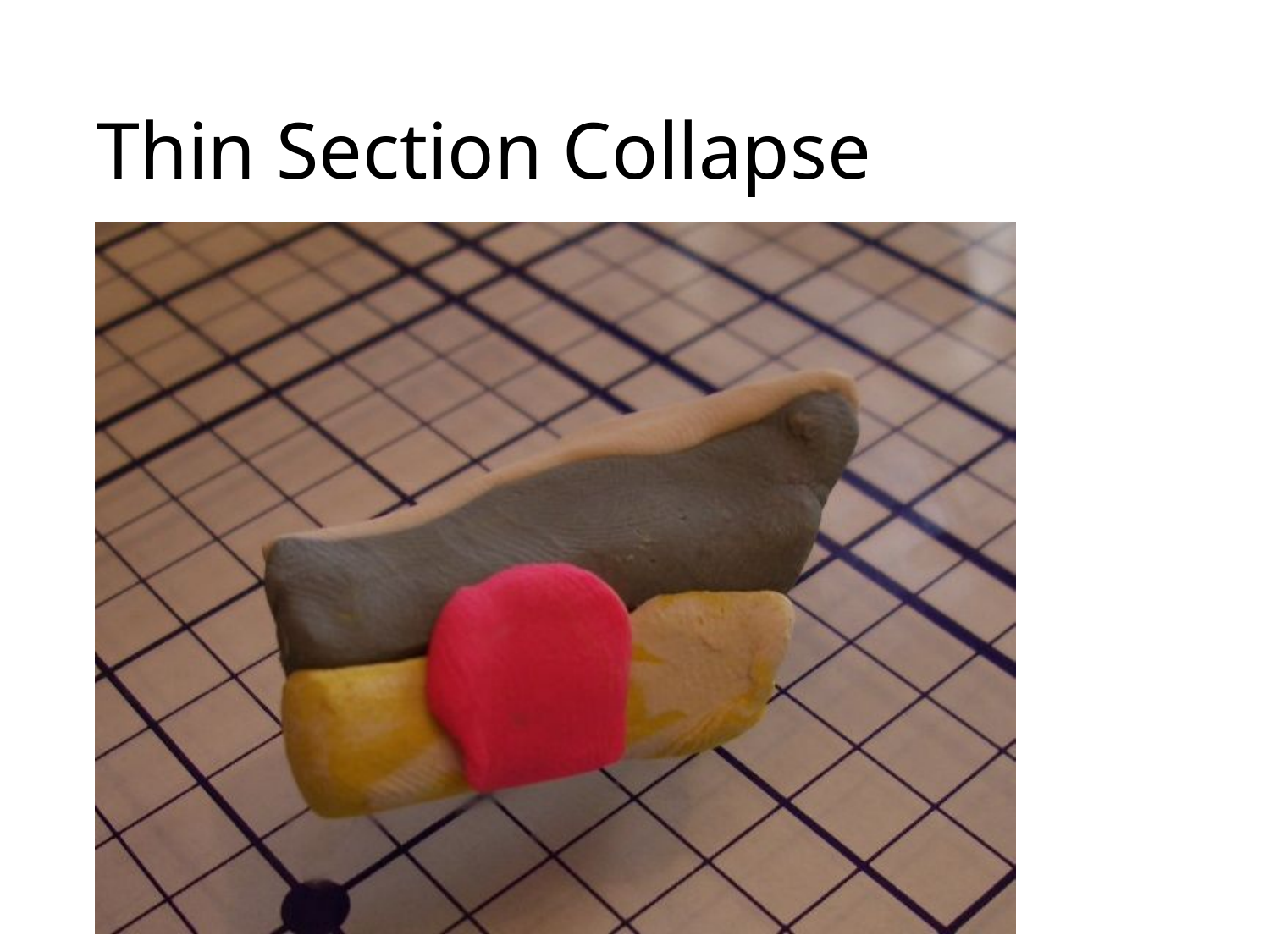

Thin Section Collapse

## Slide 10
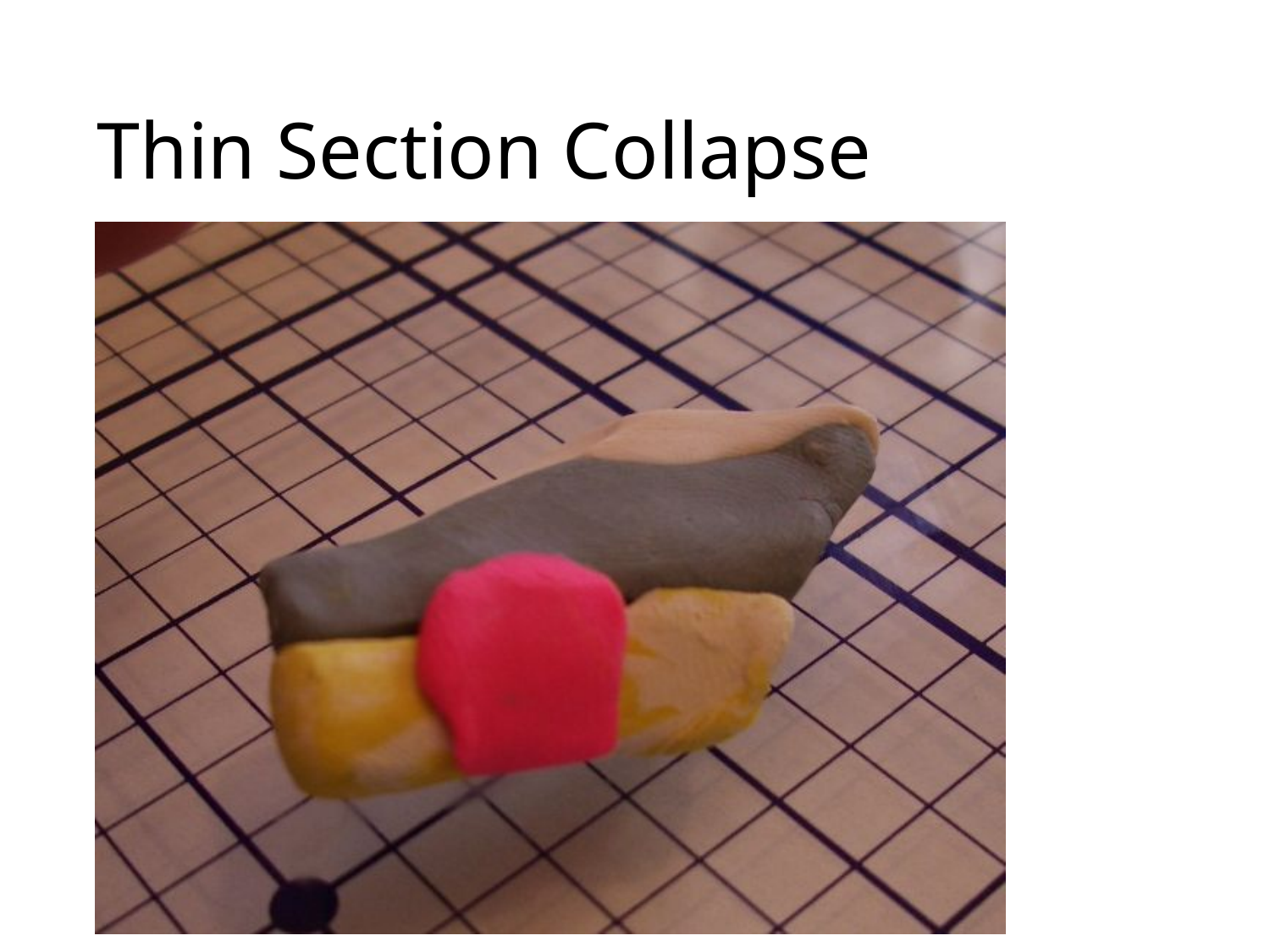

Thin Section Collapse

## Slide 11
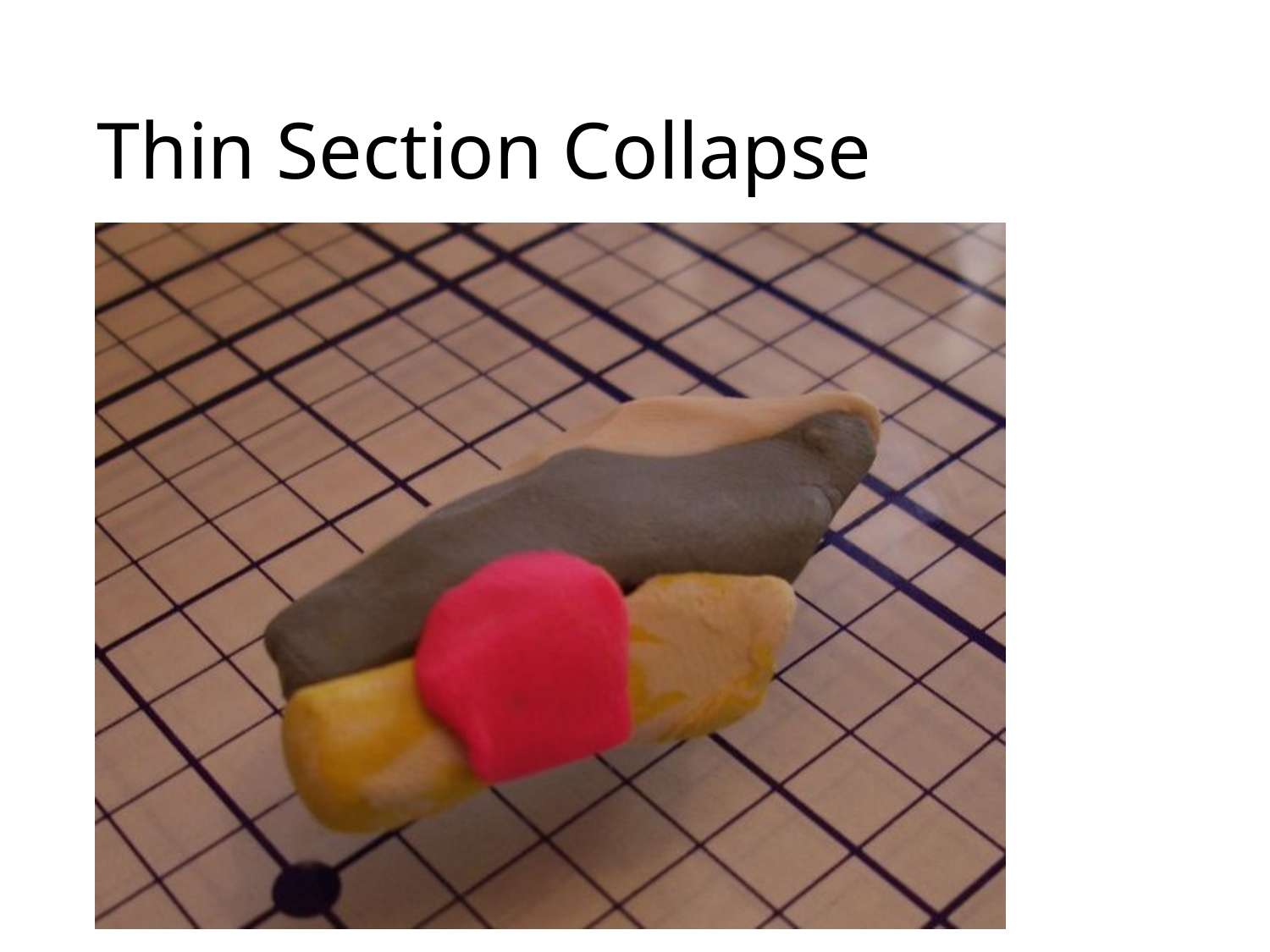

Thin Section Collapse

## Slide 12
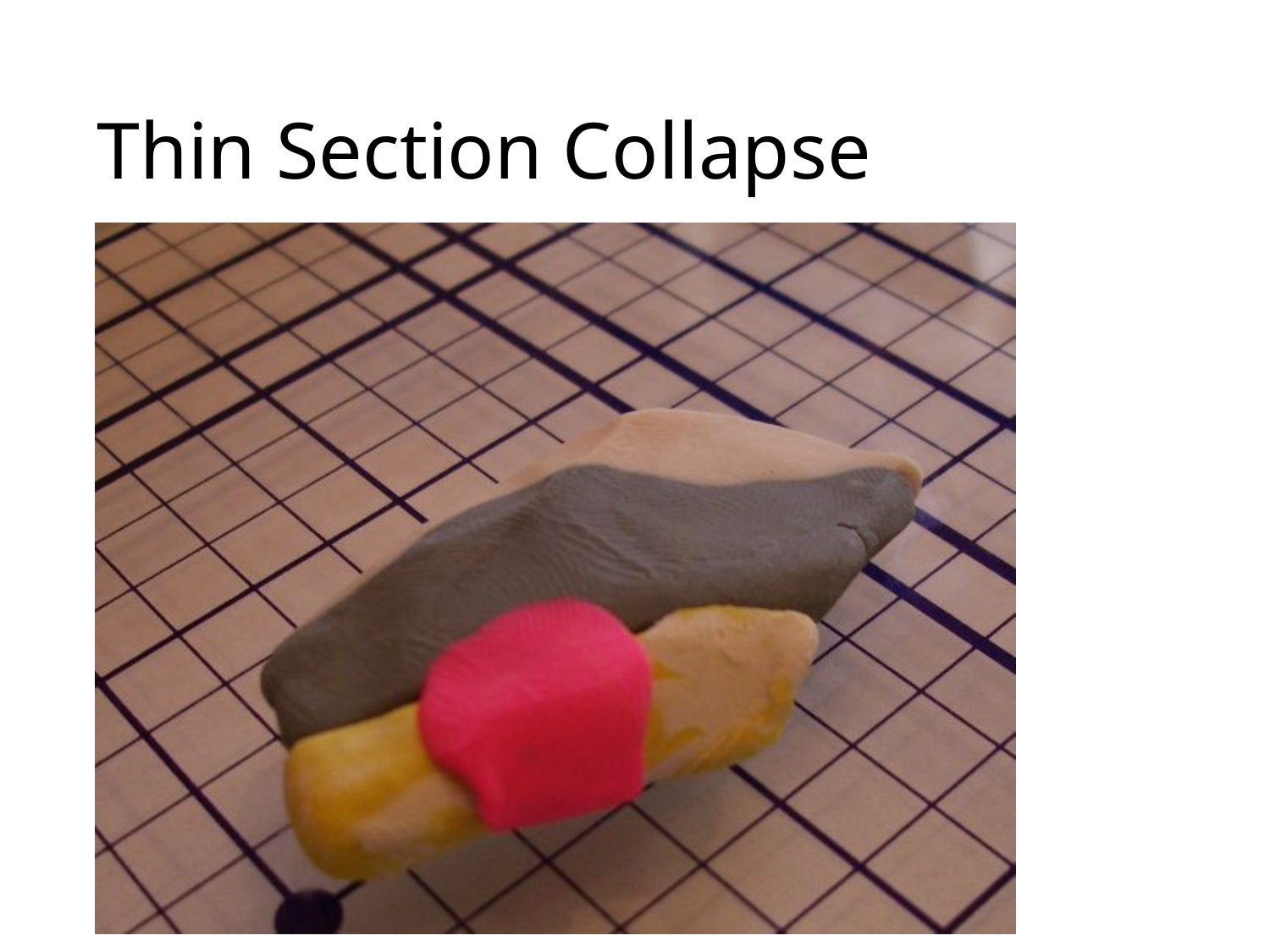

Thin Section Collapse

## Slide 13
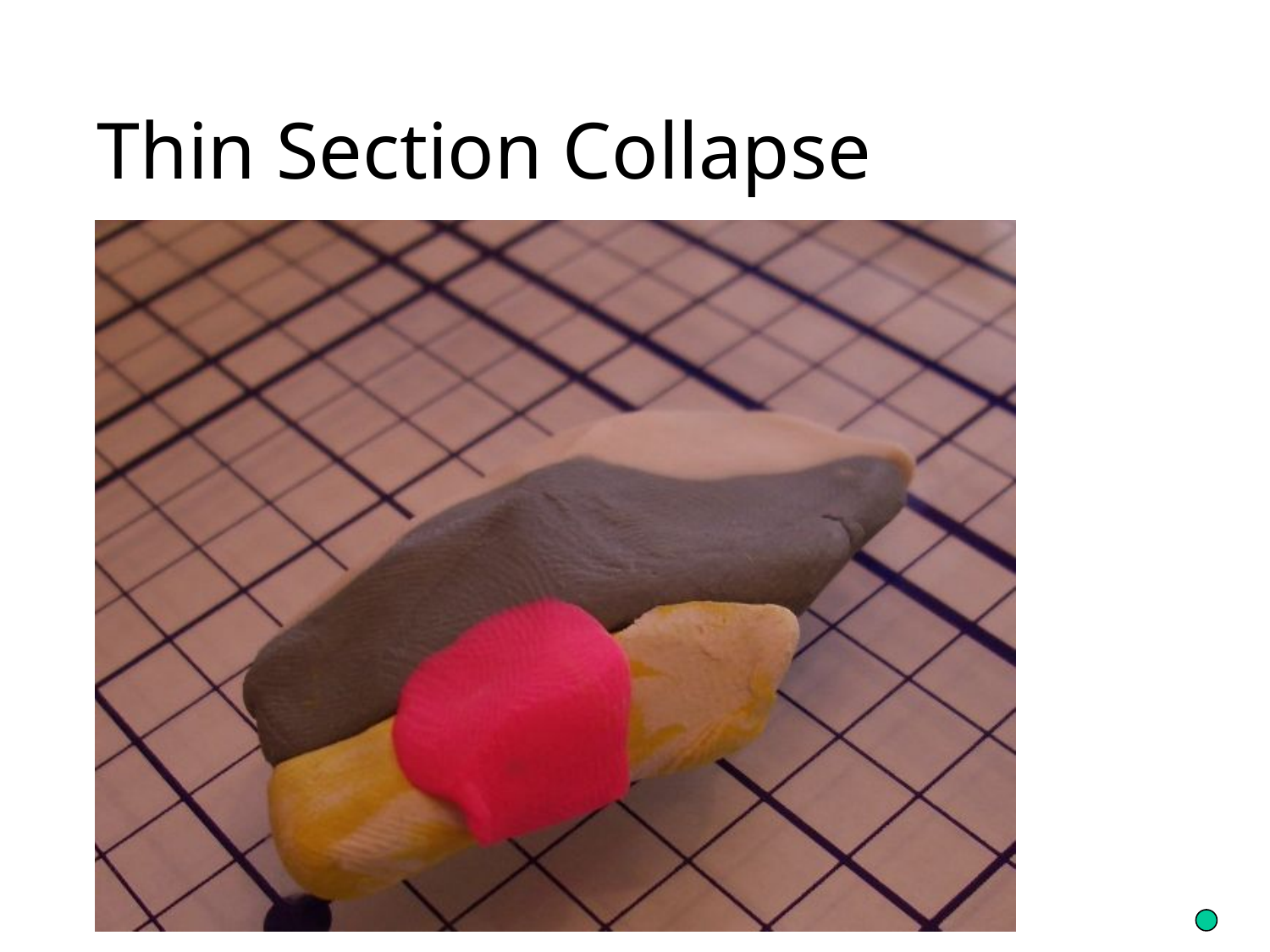

Thin Section Collapse

## Slide 14
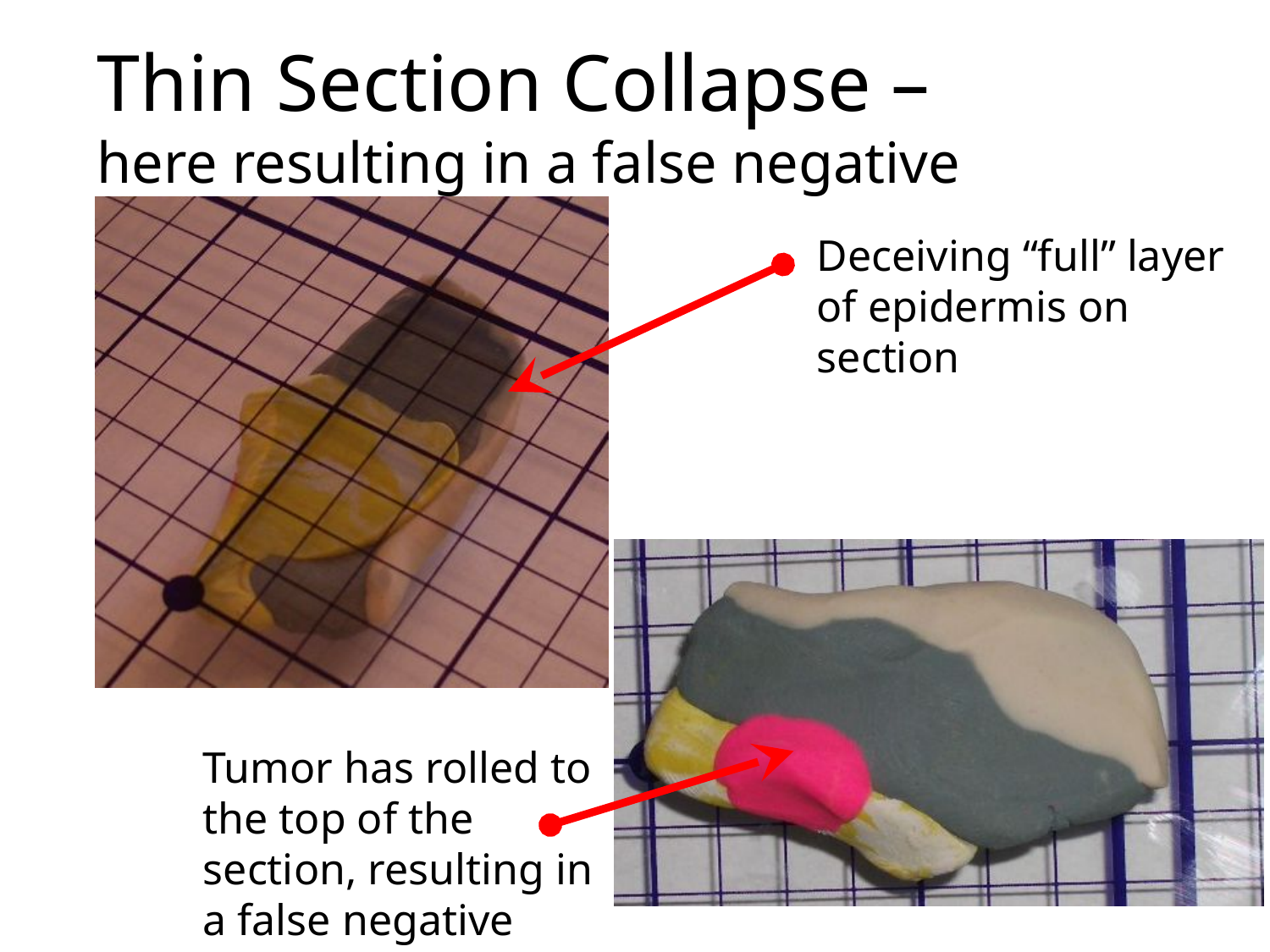

Thin Section Collapse –
here resulting in a false negative
Deceiving “full” layer of epidermis on section
Tumor has rolled to the top of the section, resulting in a false negative
